# FOXP2-positive diffuse large B-cell lymphomas exhibit a poor response to R-CHOP therapy and distinct biological signatures

**DOI:** 10.18632/oncotarget.9507

**Published:** 2016-05-20

**Authors:** Kah Keng Wong, Duncan M. Gascoyne, Elizabeth J. Soilleux, Linden Lyne, Hayley Spearman, Giovanna Roncador, Lars M. Pedersen, Michael B. Møller, Tina M. Green, Alison H. Banham

**Affiliations:** ^1^ Department of Immunology, School of Medical Sciences, Health Campus, Universiti Sains Malaysia, Kubang Kerian, Kelantan, Malaysia; ^2^ NDCLS, Radcliffe Department of Medicine, University of Oxford, John Radcliffe Hospital, Oxford, United Kingdom; ^3^ Monoclonal Antibody Unit, Centro Nacional de Investigaciones Oncológicas (CNIO), Madrid, Spain; ^4^ Department of Haematology, Roskilde Hospital, Roskilde, Denmark; ^5^ Department of Pathology, Odense University Hospital, Odense, Denmark

**Keywords:** diffuse large B-cell lymphoma, FOXP2, survival

## Abstract

FOXP2 shares partially overlapping normal tissue expression and functionality with FOXP1; an established diffuse large B-cell lymphoma (DLBCL) oncogene and marker of poor prognosis. FOXP2 is expressed in the plasma cell malignancy multiple myeloma but has not been studied in DLBCL, where a poor prognosis activated B-cell (ABC)-like subtype display partially blocked plasma cell differentiation. FOXP2 protein expression was detected in ABC-DLBCL cell lines, and in primary DLBCL samples tumoral FOXP2 protein expression was detected in both germinal center B-cell-like (GCB) and non-GCB DLBCL. In biopsies from DLBCL patients treated with immunochemotherapy (R-CHOP), ≥ 20% nuclear tumoral FOXP2-positivity (*n* = 24/158) correlated with significantly inferior overall survival (OS: *P* = 0.0017) and progression-free survival (PFS: *P* = 0.0096). This remained significant in multivariate analysis against either the international prognostic index score or the non-GCB DLBCL phenotype (*P* < 0.05 for both OS and PFS). Expression of BLIMP1, a marker of plasmacytic differentiation that is commonly inactivated in ABC-DLBCL, did not correlate with patient outcome or FOXP2 expression in this series. Increased frequency of FOXP2 expression significantly correlated with FOXP1-positivity (*P* = 0.0187), and FOXP1 co-immunoprecipitated FOXP2 from ABC-DLBCL cells indicating that these proteins can co-localize in a multi-protein complex. FOXP2-positive DLBCL had reduced expression of HIP1R (*P* = 0.0348), which is directly repressed by FOXP1, and exhibited distinct patterns of gene expression. Specifically in ABC-DLBCL these were associated with lower expression of immune response and T-cell receptor signaling pathways. Further studies are warranted to investigate the potential functional cooperativity between FOXP1 and FOXP2 in repressing immune responses during the pathogenesis of high-risk DLBCL.

## INTRODUCTION

Diffuse large B-cell lymphoma (DLBCL) is the most common subtype of non-Hodgkin's lymphoma and displays considerable heterogeneity in its genetics, clinical features and biology. Early attempts to identify DLBCL subtypes, such as the Kiel classification, distinguished categories based on tumor cell morphology as being either centroblastic or immunoblastic, the latter being associated with inferior outcome and sometimes showing plasmablastic or plasmacytoid features [1–4]. Subsequent gene expression profiling (GEP) also identified DLBCL subtypes with distinct cell-of-origin (COO) profiles, DLBCL with a germinal center B-cell-like signature (GCB-DLBCL) having a better clinical outcome than those with an activated B-cell-like (ABC-DLBCL) phenotype [5]. However, ABC-DLBCL is not a homogeneous category and reflects a spectrum of plasmablastic B-cell differentiation towards a terminally differentiated plasma cell phenotype. Indeed partial plasma cell differentiation within ABC-DLBCL has been proposed as a mechanism for loss of major histocompatibility complex class II expression in DLBCL [6], which correlates with significantly reduced survival [7, 8]. The aggressive clinical behavior of large B-cell lymphomas with plasmablastic differentiation presents a particular therapeutic challenge [9], highlighting the importance of improving our understanding of their underlying disease biology to identify new therapeutic opportunities.

The transcription factor B lymphocyte-induced maturation protein 1 (BLIMP1)/PR domain containing 1 with zinc finger domain (PRDM1) promotes the terminal differentiation of germinal center (GC) B cells into plasma cells [10–12]. In the B-cell lineage it is expressed specifically in a subset of GC centrocytes with plasmacytoid markers and in plasma cells [13, 14]. BLIMP1 acts primarily as a transcriptional repressor to extinguish the mature B-cell expression program [15], including the expression of GCB-DLBCL-associated genes such as *LMO2* and *HGAL* [16]. *BLIMP1* is specifically inactivated by structural alterations in the ABC-DLBCL subtype (24%). Many more non-GCB DLBCL tumors (77%) lack BLIMP1 protein expression, indicating that a block in post-GC cell differentiation could contribute to ABC-DLBCL pathogenesis [17]. Chromosome translocations driving expression of the BCL6 transcription factor were subsequently identified as an additional mechanism enabling transcriptional repression of *BLIMP1* in ABC-DLBCL [18]. Studies of mouse models with inactivated *Blimp1* have confirmed its function as a DLBCL tumor suppressor with a causal role in the pathogenesis of ABC-DLBCL [18, 19].

Forkhead box proteins are an evolutionarily conserved family of transcription factors with a wide range of critical biological functions and disease associations, including cellular differentiation [20]. FOXP1 has been identified as an ABC-DLBCL marker [15], whose expression correlated with poor clinical outcome in both CHOP [21, 22] and R-CHOP [23, 24] treated DLBCL patients. FOXP1 has been included in multiple immunohistochemical DLBCL subtyping algorithms aiming to distinguish DLBCL based on their COO phenotype [25–28]. In DLBCL, FOXP1 has been reported to promote B-cell proliferation [29], regulate genes involved in the germinal center reaction [30], repress the transcription of proapoptotic genes and cooperate with NF- κB to promote B-cell survival [31, 32], to potentiate WNT signaling [33], and to repress immune response signatures and MHC class II genes [32, 34]. While FOXP1 protein expression is differentially expressed in normal B cells, it is absent from most normal and malignant plasma cells [35]. More recently FOXP1 has been shown to suppress plasma cell differentiation and thus may also functionally contribute to the block of terminal B-cell differentiation in DLBCL [36].

The FOXP family (FOXP1-4) is somewhat atypical in having a zinc finger and leucine zipper domain enabling both homo- and hetero-dimer formation [37]. Partially overlapping expression patterns and phenotypes, particularly of FOXP1 and FOXP2 in neurodevelopment and cognitive disorders [38] and in the lung [39–41], have indicated that these molecules have both shared and distinct biological functions. Furthermore, specific combinations of FOXP1/2/4 dimers are able to differentially fine-tune the expression of individual genes involved in the WNT and Notch pathways [42], which are both implicated in DLBCL pathogenesis. Existing data suggest that FOXP1 and FOXP2 generally show reciprocal patterns of expression during terminal B-cell differentiation and in B-cell malignancies. FOXP2 being absent in normal B cells and most B-cell lymphoma cell lines while being expressed in a subpopulation of normal plasma cells and in plasma cell dyscrasias, such as monoclonal gammopathy of undetermined significance (MGUS) and myeloma [43].

As DLBCL represents a spectrum of plasmablastic differentiation and a block in this process is causally involved in disease pathogenesis, we were interested to observe strong FOXP1 and FOXP2 co-expression in the ABC-DLBCL cell line OCI-Ly10 [43]. This, and the expression of FOXP2 in MGUS and myeloma, raised the possibility that FOXP2, like FOXP1, might also be involved in DLBCL pathogenesis. Furthermore, if these transcription factors were co-expressed in DLBCL tumor cells, there might be a physical and functional FOXP1-FOXP2 interaction. Here we present the first study of FOXP2 expression in primary DLBCL, its relationship with COO subtypes, FOXP1, other molecules involved in B-cell differentiation and clinicopathological data. The poor clinical outcome associated with FOXP2 expression in DLBCL together with its co-expression and co-immunoprecipitation with FOXP1 indicates that further studies are warranted to understand their potential cooperativity and contribution to DLBCL pathogenesis.

## RESULTS

### FOXP2 expression in DLBCL cell lines

**Figure 1:**
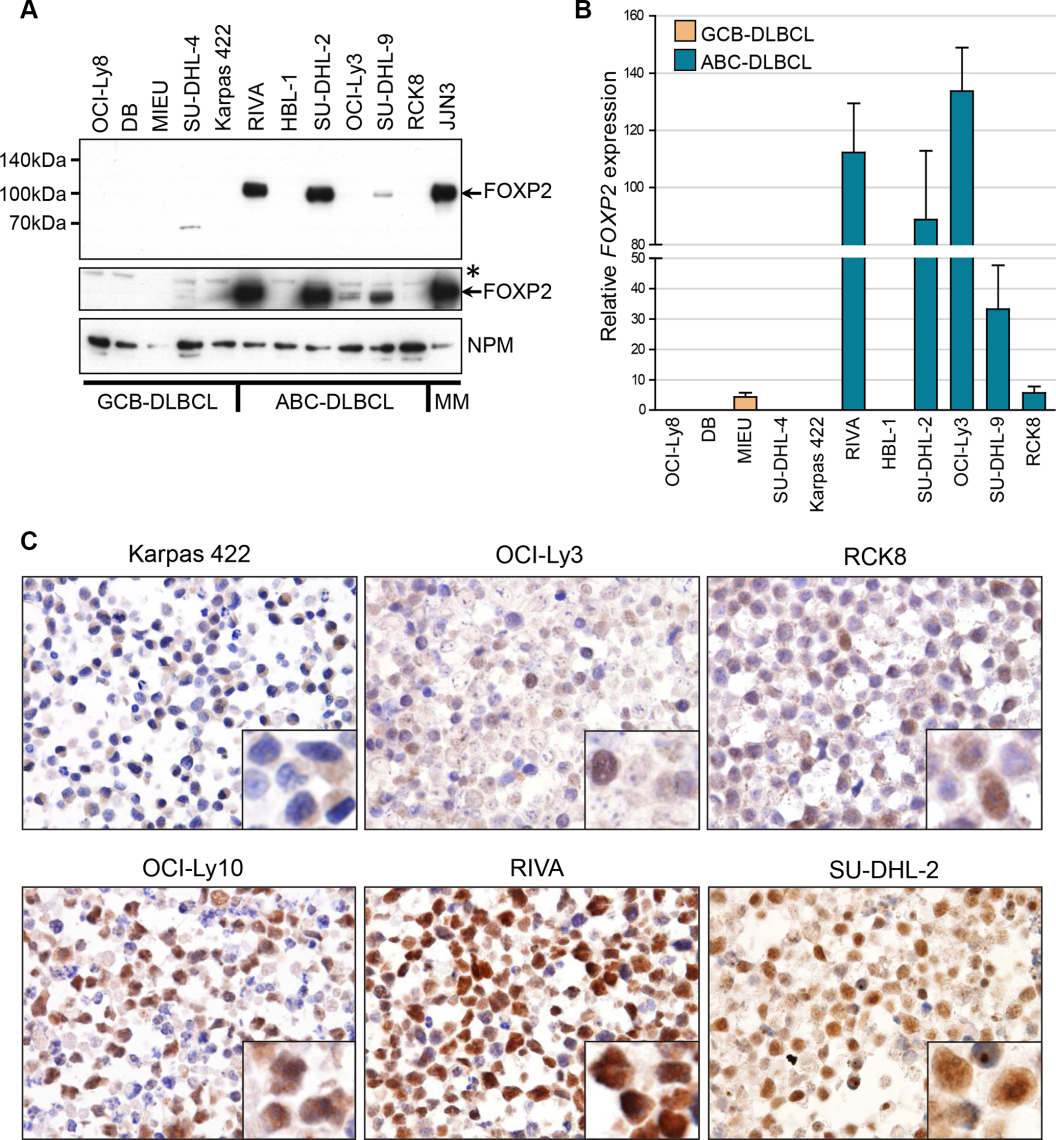
FOXP2 is expressed in ABC-DLBCL cell lines (**A**) Western blotting of nuclear lysates from GCB-DLBCL, ABC-DLBCL and myeloma cell lines with anti-FOXP2 (clone 73A/8) and anti-NPM (clone NA24) antibodies. An overexposed image (middle panel) shows very weak FOXP2 expression in OCI-Ly3 and just detectable expression in RCK8. *Indicates an upper band that is thought to be non-specific. MIEU remained negative in other experiments with more abundant sample loading. (**B**) qRT-PCR for *FOXP2* transcripts in GCB- and ABC-DLBCL cell lines. *FOXP2* expression was normalized according to the expression of 18S rRNA housekeeping gene and expressed relative to JJN3 (set to 100%). (**C**) Immunohistochemical labeling of FOXP2 nuclear expression in DLBCL cell lines: showing negative (Karpas 422), weak (OCI-Ly3, RCK8) and strongly positive (OCI-Ly10, RIVA, SU-DHL-2) examples. Higher power insets are shown in the bottom right corners of each panel.

Our prior study of FOXP2 expression in cell lines derived from lymphoid malignancies detected expression primarily in multiple myeloma and Hodgkin's lymphoma [43]. However, the ABC-DLBCL cell line OCI-Ly10 also showed comparable FOXP2 protein and transcript levels to those in myeloma and Hodgkin's lymphoma lines [43]. Western blotting and qRT-PCR (Figure 1A–1B) across an extended panel of eleven DLBCL cell lines identified the ABC-DLBCL cell lines RIVA and SU-DHL-2 as also being strongly FOXP2-positive, with levels comparable to that in the myeloma cell line JJN3. Some nuclear FOXP2 protein expression was also detectable in SU-DHL-9 and much weaker expression in the cell lines OCI-Ly3 and RCK8 (the latter only when blots were overexposed), all of which are ABC-DLBCL. OCI-Ly3 exhibited comparable transcript levels to the cell lines with abundant FOXP2 protein expression, suggesting the differential detection of FOXP2 isoforms (*e.g.* FOXP2 proteins lacking the N-terminal FOXP2-73A/8 antibody epitope may be expressed) or that posttranscriptional mechanisms may restrict FOXP2 protein expression in this cell line. Thus 3/7 ABC-DLBCL cell lines strongly expressed FOXP2 (OCI-Ly10, RIVA and SU-DHL-2), while a further 3/7 were weakly FOXP2-positive (SU-DHL-9, OCI-Ly3 and RCK8). Immunohistochemistry confirmed predominantly nuclear FOXP2 protein expression and a minority of positive nuclei in RCK8 and OCI-Ly3 was consistent with weak expression detectable by blotting (Figure 1C). None of five GCB-DLBCL cell lines exhibited detectable FOXP2 nuclear protein expression, although MIEU did express *FOXP2* transcripts (Figure 1B).

### Primary DLBCL patients with FOXP2-positive tumors have a poor clinical outcome

**Figure 2:**
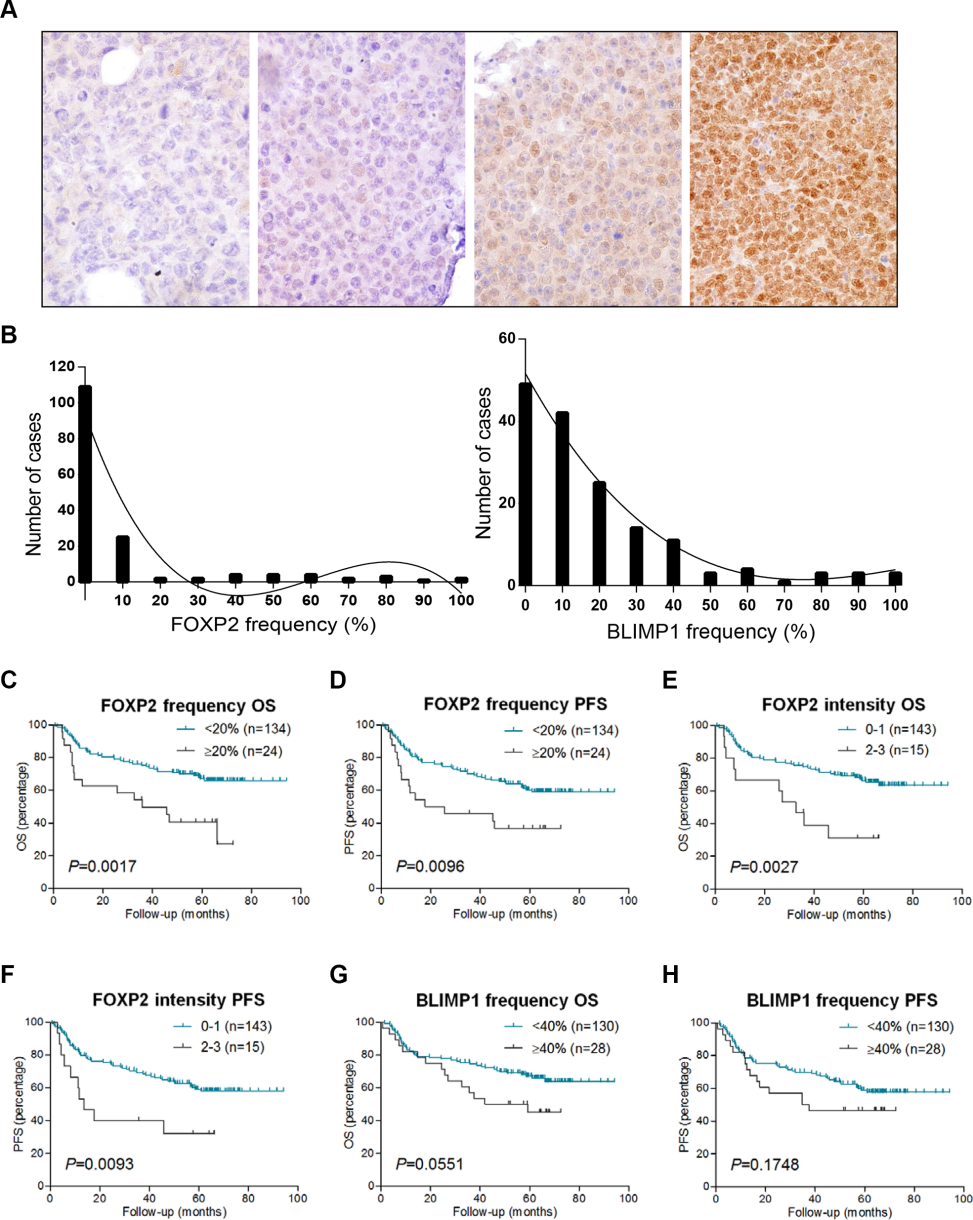
FOXP2 expression confers inferior survival in R-CHOP-treated DLBCL cases (**A**) Immunohistochemical labeling of FOXP2 expression in primary DLBCL cases with variable intensity and frequency of expression. Left to right these were scored as negative, intensity 1/frequency 55%, intensity 2/frequency 80%, intensity 3/frequency 100%. (**B**) Frequency distribution of FOXP2 (left panel) and BLIMP1 (right panel) at each 10% cut-off level in DLBCL cases (*n* = 158). (**C**–**H**) Overall survival (OS) and progression free survival (PFS) of DLBCL cases (*n* = 158) according to FOXP2 frequency at 20% cut-off (C–D) or intensity (E–F), and BLIMP1 frequency at 40% cut-off (G–H).

Given the expression of FOXP2 in myeloma [43] and in the majority of ABC-DLBCL cell lines, we investigated whether it might be a potential marker of plasma cell differentiation in DLBCL. BLIMP1 expression was also analyzed in the same series to determine whether FOXP2 had any relationship with this established marker of plasma cell differentiation. We analyzed both the frequency and intensity of tumoral nuclear FOXP2 and BLIMP1 protein expression in 158 primary DLBCL using immunohistochemistry. DLBCL tumors exhibiting variable intensity and frequency of FOXP2 expression are illustrated in Figure 2A. The frequency of expression of both proteins exhibited a non-Gaussian distribution: FOXP2 (*P* < 0.0001); BLIMP1 (*P* = 0.0029) (Figure 2B).

Nuclear FOXP2 expression was detectable in 49/158 (31%) of the DLBCL patients (Figure 2), the median frequency being 0% and the mean frequency being 10%. No frequency threshold for FOXP2 expression had been previously defined in DLBCL, hence we tested the clinical relevance of 10% thresholds and selected ≥ 20% positivity, which identified 24 FOXP2-positive patients (15%). DLBCL patients with ≥ 20% nuclear FOXP2-positivity exhibited significantly inferior overall survival (OS: *P* = 0.0017) and progression free survival (PFS: *P* = 0.0096) (Figure 2C–2D). The qualitative intensity of FOXP2 expression (score 0–1 versus 2–3) also identified a subgroup of DLBCL patients (*n* = 15) with poor clinical outcome (OS, *P* = 0.0027; PFS, *P* = 0.0093) (Figure 2E–2F).

The median frequency of BLIMP1 protein expression in this DLBCL series was 10% and the mean was 19%. A variety of BLIMP1 thresholds (*e.g.* 10–30%) have been used for correlation with clinical outcome. Here we selected a ≥ 40% cut-off, as there was a trend towards an association with inferior OS (*P* = 0.0551, Figure 2G) that was not observed at lower thresholds. No association with PFS was observed at ≥ 40% cut-off (*P* = 0.1748, Figure 2H), and the qualitative intensity of BLIMP1 expression had no clinical relevance in this series (data not shown).

### FOXP2 is a risk factor independent of either IPI or DLBCL COO in multivariate analyses

Frequency-defined FOXP2 and BLIMP1 categories were independent of patients' clinical characteristics such as age, sex, performance status, the number of extranodal sites and their International Prognostic Index (IPI) risk group (Table 1). In multivariate analyses (Table 2), DLBCL patients with ≥ 20% FOXP2 expression exhibited significantly inferior outcome independent of either a high IPI score (OS: *P* = 0.0119; PFS: *P* = 0.0451) or non-GCB DLBCL subtype according to Hans (OS: *P* = 0.0047; PFS: *P* = 0.0186), Choi (OS: *P* = 0.0059; PFS: *P* = 0.0258) and Visco-Young (OS: *P* = 0.0098; PFS: *P* = 0.0367) algorithms.

**Table 1:** Clinical and hematologic characteristics of DLBCL patients stratified according to FOXP2 and BLIMP1 expression

Characteristics	All Cases (*n* = 158)	FOXP2 < 20% (*n* = 134)	FOXP2 ≥ 20% (*n* = 24)	*P*-value	BLIMP1 < 40% (*n* = 130)	BLIMP1 ≥ 40% (*n* = 28)	*P*-value
**Age (years)**							
Median	67	67	72	0.4519	67	69	0.4930
Range	20–91	20–91	58–87		20–91	44–90	
**Sex**							
Female (%)	70	60 (38)	10 (6)	0.7773	57 (36)	13 (8)	0.8034
Male (%)	88	74 (47)	14 (9)		73 (46)	15 (9)	
**Stage**							
I–II (%)	86	74 (47)	12 (8)	0.6360	73 (46)	13 (8)	0.3488
III–IV (%)	72	60 (38)	12 (8)		57 (36)	15 (9)	
**Performance status**							
0–1 (%)	136	117 (74)	19 (12)	0.2884	113 (72)	23 (15)	0.5076
≥ 2 (%)	22	17 (11)	5 (3)		17 (11)	5 (3)	
**LDH**							
< ULN (%)	94	82 (52)	12 (8)	0.3037	80 (51)	14 (9)	0.2592
≥ ULN (%)	64	52 (33)	12 (8)		50 (32)	14 (9)	
**Extranodal sites**							
0–1 (%)	133	113 (72)	20 (13)	0.9025	110 (70)	23 (15)	0.7447
≥ 2 (%)	25	21 (13)	4 (3)		20 (13)	5 (3)	
**IPI**							
0–2 (%)	111	98 (62)	13 (8)	0.0612	93 (59)	18 (11)	0.4463
3–5 (%)	47	36 (23)	11 (7)		37 (23)	10 (6)	
**COO (Hans)**							
GCB (%)	89	78 (49)	11 (7)	0.2603	80 (51)	9 (6)	**0.0044**
Non-GCB (%)	69	56 (35)	13 (8)		50 (32)	19 (12)	
COO (Choi; *n* = 155)*							
GCB (%)	95	83 (54)	12 (8)	0.2167	84 (54)	11 (7)	**0.0159**
Non-GCB (%)	60	48 (31)	12 (8)		44 (28)	16 (10)	
**COO (Visco- Young; *n* = 157)†**							
GCB (%)	98	87 (55)	11 (7)	0.0683	85 (54)	13 (8)	0.0924
Non-GCB (%)	79	46 (29)	13 (8)		45 (29)	14 (9)	

* Three cases (of 158) could not be classified for COO according to Choi algorithm;

† One case (of 158) could not be classified for COO according to Visco-Young algorithm; ULN: Upper limit of normal.

**Table 2:** Multivariate analysis for OS and PFS in DLBCL patients treated with R-CHOP

Risk Factor	OS			PFS		
	95% CI	Hazard Ratio	*P*-value	95% CI	Hazard Ratio	*P*-value
IPI ≥ 3	1.67–4.73	2.81	**0.0001**	1.82–4.83	2.97	**< 0.0001**
FOXP2 ≥ 20%	1.18–3.87	2.14	**0.0119**	1.01–3.23	1.81	**0.0451**
Non-GCB phenotype (Hans algorithm)	0.99–2.79	1.66	0.0539	0.98–2.55	1.58	0.0616
FOXP2 ≥ 20%	1.30–4.22	2.34	**0.0047**	1.12–3.56	2.00	**0.0186**
Non-GCB phenotype (Choi algorithm)	1.05–2.94	1.75	**0.0323**	1.06–2.76	1.71	**0.0292**
FOXP2 ≥ 20%	1.27–4.15	2.30	**0.0059**	1.08–3.45	1.93	**0.0258**
Non-GCB phenotype (Visco-Young algorithm)	1.16–3.31	1.96	**0.0118**	1.19–3.14	1.93	**0.0079**
FOXP2 ≥ 20%	1.21–4.00	2.20	**0.0098**	1.04–3.34	1.86	**0.0367**

### FOXP2 expression does not correlate with DLBCL COO subtype and identifies high-risk GCB and non-GCB DLBCL patients

**Figure 3:**
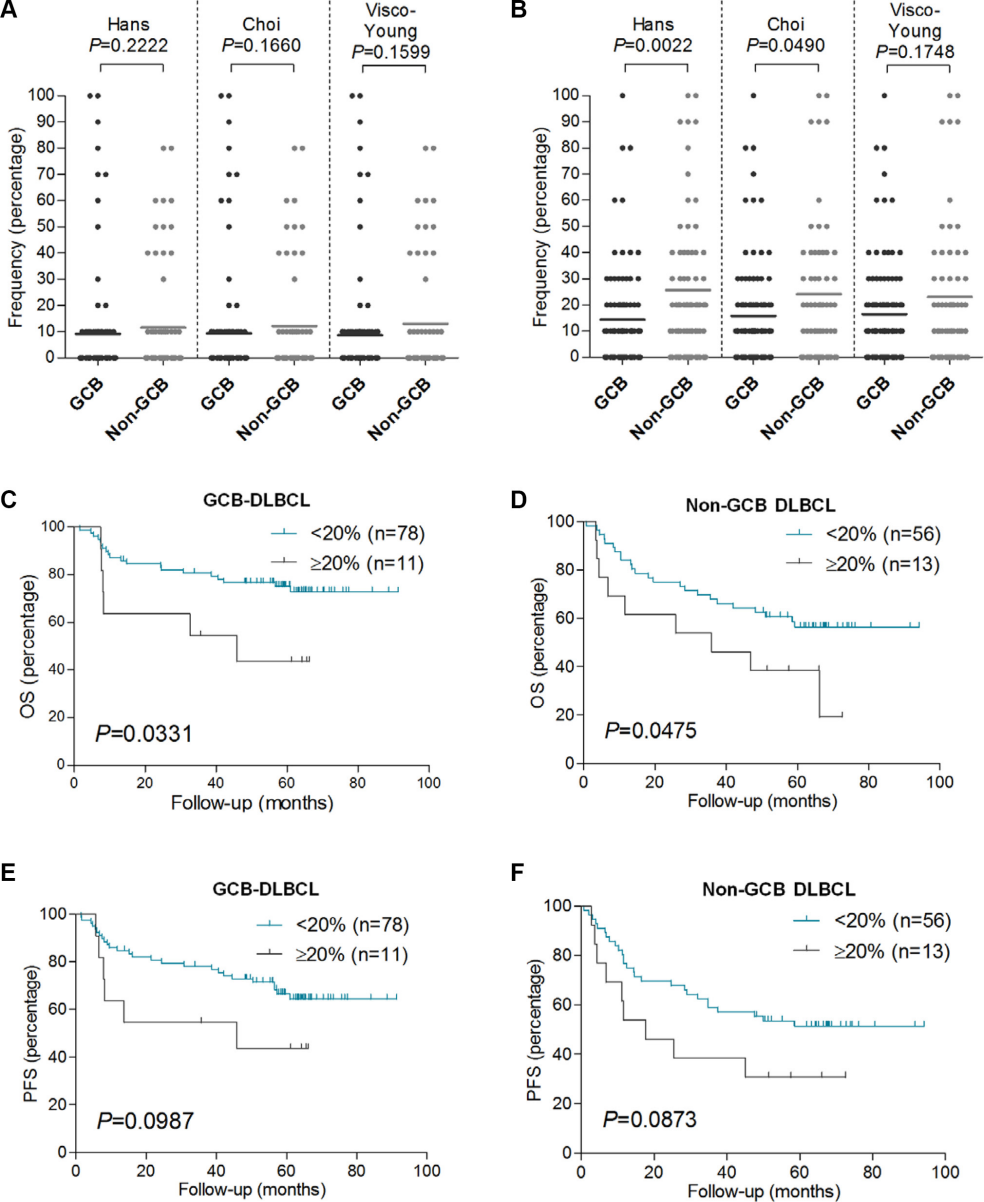
FOXP2 expression is not associated with non-GCB DLBCL primary cases, and it confers worse OS in GCB or non-GCB DLBCL subtypes (**A**–**B**) Relationship of FOXP2 (A) or BLIMP1 (B) protein expression frequency with GCB or non-GCB DLBCL subtype according to Hans (*n* = 158), Choi (*n* = 155) or Visco-Young (*n* = 157) immunohistochemical algorithms. (**C**–**F**) OS (C–D) and PFS (E–F) in GCB-DLBCL (*n* = 89) or non-GCB DLBCL (*n* = 69) according to FOXP2 20% cut-off. The DLBCL cases were stratified according to Hans algorithm.

GCB or non-GCB COO subtyping had already been demonstrated to define clinically relevant subgroups in this DLBCL series [23]. The frequency of FOXP2 protein expression exhibited no relationship with DLBCL COO subtype, defined using three (Hans, Choi and Visco-Young) immunohistochemical algorithms (Table 1, Figure 3A). In contrast, non-GCB DLBCL defined by two of the three COO algorithms exhibited elevated BLIMP1 expression, using either a 40% cut-off (Table 1: Hans *P* = 0.0044; Choi *P* = 0.0159) or 10% frequency increments (Figure 3B: Hans *P* = 0.0022; Choi *P* = 0.0490). DLBCL subtypes defined using the Visco-Young algorithm showed no relationship with BLIMP1 expression (Table 1, Figure 3B). The FOXP2 or BLIMP1 intensity scores exhibited no relationship with DLBCL COO subtype (data not shown).

Increased frequency of FOXP2 expression identified patients with significantly inferior OS in both the GCB (*P* = 0.0331) and non-GCB (*P* = 0.0475) subgroups of DLBCL patients (Figure 3C–3D). There was also a trend towards inferior PFS in both the GCB (*P* = 0.0987) and non-GCB (*P* = 0.0873) DLBCL subgroups that did not reach statistical significance (Figure 3E–3F). FOXP2 expression was not clinically relevant in low (0–2) versus high (3–5) risk IPI subgroups of DLBCL patients (data not shown).

### FOXP1 and FOXP2 are co-expressed in high-risk DLBCL and can be co-immunoprecipitated in ABC-DLBCL cells

While FOXP2 expression did not correlate with DLBCL COO subtype, this did not exclude the possibility of there being relationships between FOXP2 and individual molecules involved in B-cell differentiation. These potential relationships were investigated by comparing both categorical (defined by threshold of expression) and continuous variables (frequency in 10% increments) (Tables 3 and 4, respectively). FOXP2-positivity (≥ 20%) correlated with reduced expression (≤ 10%) of the direct FOXP1 target HIP1R (*P* = 0.0348) and increased expression of MUM1/IRF4 (*P* = 0.0075). MUM1/IRF4 was the only marker that correlated with FOXP2 as both a categorical and continuous variable (*P* = 0.0476). There was no correlation between FOXP2 and BLIMP1 as either continuous (*P* = 0.1727) or categorical (*P* = 0.3820) variables.

**Table 3:** Correlation between FOXP2 and other markers as categorical variables

Marker & cut-off value		FOXP2		*P*-value
		≥ 20%	≥ 20%	
**CD10**	< 30%	73	14	0.7542
	≥ 30%	60	10	
**BCL6**	< 30%	16	4	0.5128
	≥ 30%	117	20	
**GCET1**	< 60%	78	18	0.1303
	≥ 60%	55	6	
**HIP1R**	≤ 10%	33	11	**0.0348**
	> 10%	100	13	
**MUM1/ IRF4**	< 30%	67	5	**0.0075**
	≥ 30%	66	19	
**FOXP1**	< 70%	62	5	**0.0187**
	≥ 70%	71	19	
**BLIMP1**	< 40%	112	18	0.3820
	≥ 40%	22	6	
**HLA-DRA**	< 90%	38	8	0.6213
	≥ 90%	96	16	

**Table 4:** Correlation between FOXP2 and other markers as continuous variables

Marker	FOXP2	
	Pearson correlation	*P*-value
**CD10**	−0.0326	0.6854
**BCL6**	−0.0878	0.2745
**GCET1**	−0.1815	**0.0229**
**HIP1R**	−0.1083	0.1768
**MUM1/IRF4**	0.1584	**0.0476**
**FOXP1**	0.1259	0.1162
**BLIMP1**	0.1090	0.1727
**HLA-DRA**	–0.0616	0.4420

**Figure 4:**
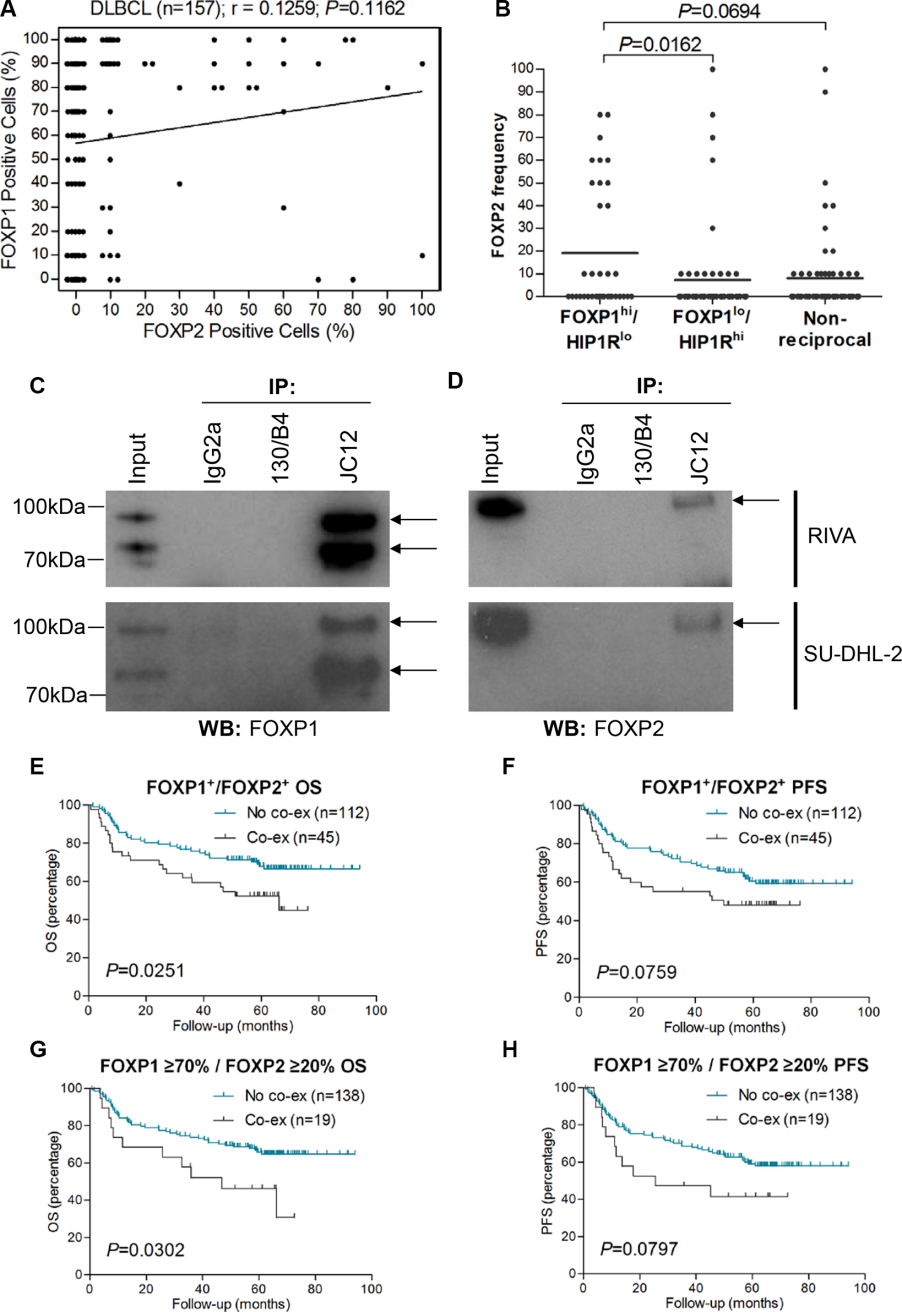
FOXP2 is co-immunoprecipitated by FOXP1 and their co-expression confers worse survival in R-CHOP-treated DLBCL cases (**A**) Correlation of FOXP1 and FOXP2 expression frequency in DLBCL (*n* = 157). (**B**) Relationship of FOXP2 frequency with FOXP1^hi^/HIP1R^lo^ (*n* = 36), FOXP1^lo^/HIP1R^hi^ (*n* = 58) or non-reciprocal (*n* = 62) DLBCL cases. (**C**–**D**) Co-immunoprecipitation of endogenous FOXP1 in RIVA and SU-DHL-2 cell lysates with anti-FOXP1 JC12 antibody or with non-specific IgG2a isotype and 130/B4 (“in house” anti-NFIL3) control antibodies. Input indicates non-immunoprecipitated cell lysates. JC12 blotting; top arrow indicates FOXP1_FL_, bottom arrow indicates FOXP1_s_ (C). Co-immunoprecipitated FOXP2 was detected by Western blotting the JC12 immunoprecipitated proteins with the FOXP2-73A/8 antibody (D).

No significant correlation was identified between the frequency of FOXP2 expression and that of its family member FOXP1 as continuous variables (Figure 4A, Table 4). However, of the 24 DLBCL patients with ≥ 20% FOXP2 expression, only 5 exhibited < 70% FOXP1 expression and a significant enrichment of FOXP2-positive DLBCL (≥ 20% FOXP2 positivity) within the FOXP1-positive category (≥ 70% FOXP1) was observed (*P* = 0.0187; Table 3). The frequency of FOXP2 expression was also higher in DLBCL with a FOXP1^hi^HIP1R^lo^ phenotype (DLBCL with FOXP1 short isoforms potentially capable of transcriptionally repressing its direct target gene *HIP1R*) than those with FOXP1^lo^/HIP1R^hi^ expression (*P* = 0.0162: Figure 4B). Patients with a FOXP1^hi^HIP1R^lo^ phenotype were previously reported in both GCB and non-GCB subtypes and exhibited significantly inferior survival in this series [23].

Twelve tumors displayed ≥ 50% frequency of expression of both transcription factors, indicating that FOXP1 and FOXP2 can be co-expressed at the single-cell level in a subset of primary DLBCL. Immunoprecipitation of FOXP1 from the ABC-DLBCL cell lines RIVA and SU-DHL-2, that have robust expression of both FOXP1 and FOXP2, co-immunoprecipitated FOXP2 (Figure 4C–4D). These data demonstrate that FOXP1 and FOXP2 exist in a multi-protein complex and could potentially heterodimerize in ABC-DLBCL cells. The 45 DLBCL tumors with any frequency of FOXP1 and FOXP2 co-expression exhibited significantly inferior OS (*P* = 0.0251) and a trend towards inferior PFS (*P* = 0.0759) (Figure 4E–4F). Nineteen patients exhibited ≥ 70% FOXP1 and ≥ 20% FOXP2 expression. These also exhibited significantly inferior OS (*P* = 0.0302) and a trend towards inferior PFS (*P* = 0.0797) when compared to those patients (*n* = 138*)* that lacked FOXP1/FOXP2 co-expression (Figure 4G–4H).

### DLBCL tumors with FOXP2 protein expression have distinct gene expression profiles

**Figure 5:**
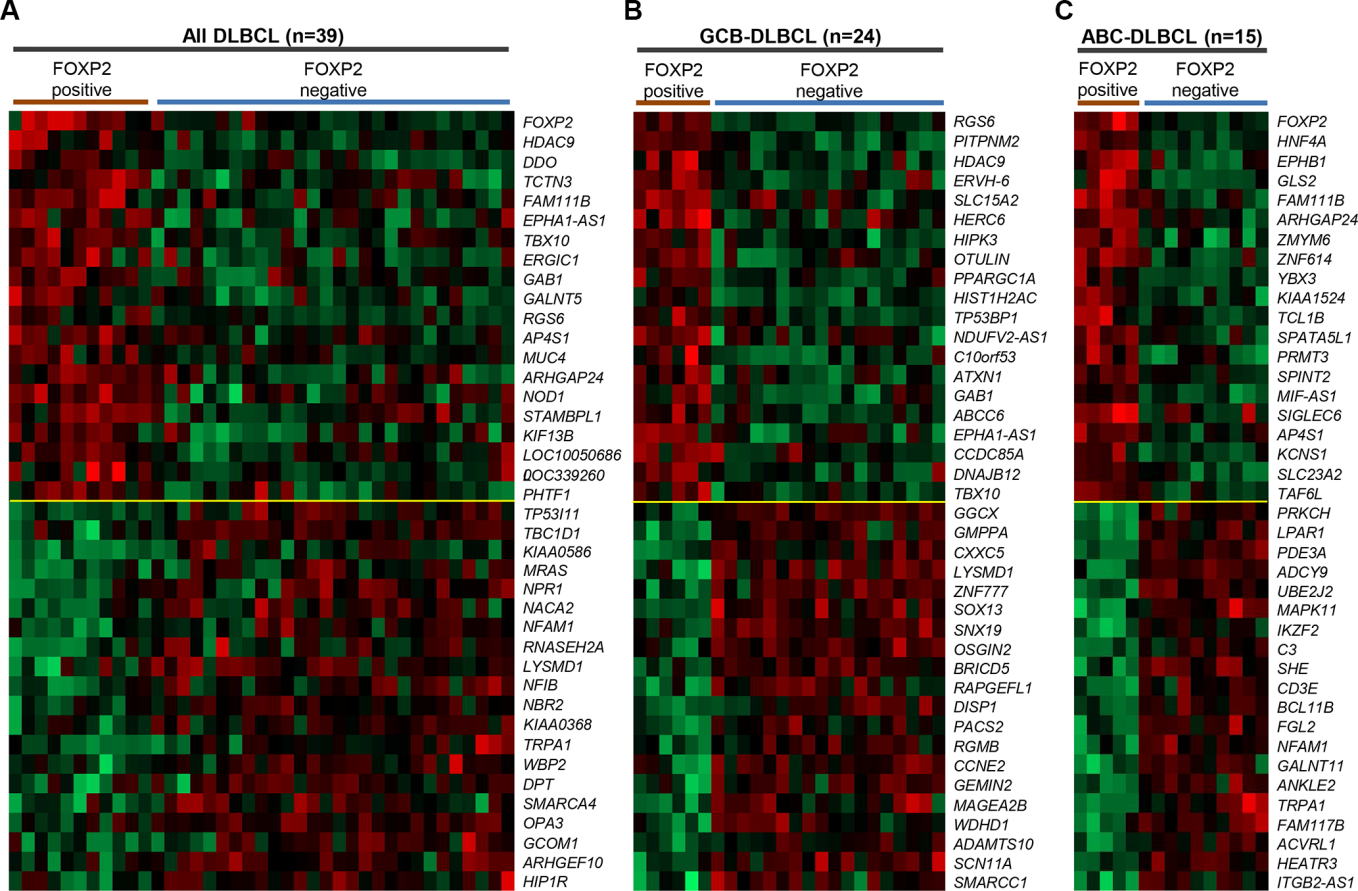
Genes most highly-correlated with FOXP2 protein expression in primary DLBCL cases (**A**–**C**) Top 20 genes positively- or inversely-correlated with FOXP2 protein positive expression in all DLBCL cases (*n* = 39; A), GCB-DLBCL (*n* = 24; B) or ABC-DLBCL (*n* = 15; C) subtype. The cases were stratified according to FOXP2 protein positive (*i.e.* absolute positivity without regarding frequency cut-off) or negative (*i.e.* 0% frequency). FOXP2 protein was positive in six (of 24) GCB-DLBCL and five (of 15) ABC–DLBCL cases.

To investigate the molecular mechanisms by which FOXP2 expression might contribute to aggressive disease and thus a poor clinical outcome, we compared the gene expression profiles of DLBCL patients with (*n* = 11) and without (*n* = 28) FOXP2 protein expression using an existing gene expression dataset (GSE31312) that was available for a subset of patients from this cohort (Figure 5A; Table S1). This analysis identified distinct genes whose up- or down-regulation distinguished FOXP2-positive versus -negative DLBCL. Upregulated genes included *FOXP2* itself, identifying the microarray probe (1555516_at) that most significantly correlated with FOXP2 protein expression in these samples (*P* < 0.0001). One of the most downregulated genes, *HIP1R*, is a known directly FOXP1 repressed gene [23] and HIP1R protein exhibited an inverse relationship with FOXP2 in the current study. Subdividing patients into microarray-defined GCB-DLBCL or ABC-DLBCL subgroups showed different genes associated with FOXP2-positivity (Figure 5B–5C; Table S1), with no overlap in the individual top 20 up- and down-regulated genes between GCB-DLBCL and ABC-DLBCL subgroups.

**Figure 6:**
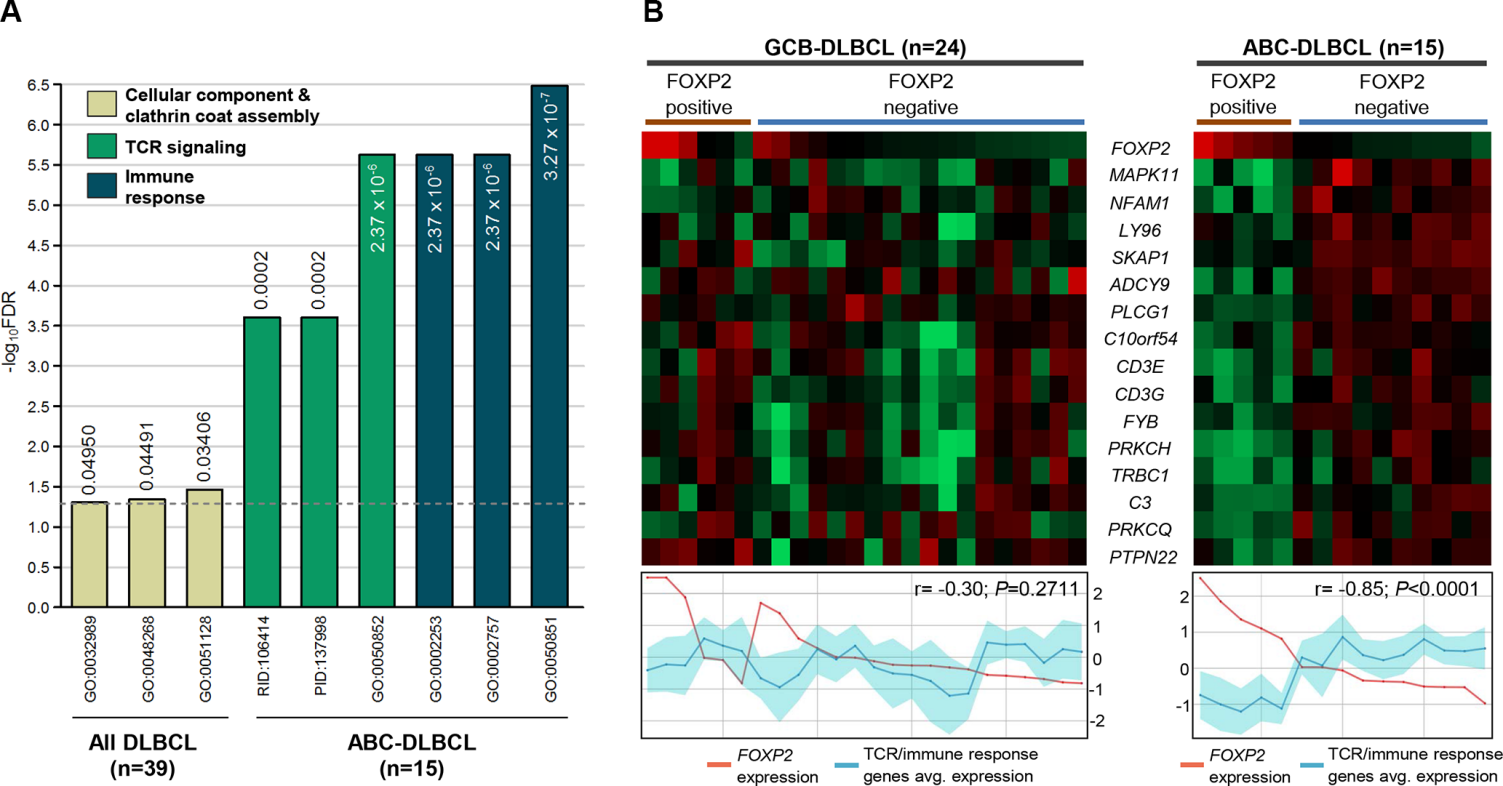
TCR signaling and immune response signatures were significantly reduced in FOXP2-positive ABC-DLBCL cases (**A**) Gene ontology and pathway signatures suppressed in FOXP2-positive DLBCL cases compared with their FOXP2-negative counterparts. All DLBCL cases (*n* = 39) or ABC-DLBCL (*n* = 15) molecular subtype were analyzed separately. The bar graph was constructed on a negative log_10_ scale with higher -log_10_ value denoting more significant FDR value. The linear FDR values were shown next to or within each bar, and the horizontal dotted line denotes linear FDR threshold of 0.05. GO: Gene Ontology; PID: Pathway Interaction Database; RID: Reactome ID. (**B**) Heatmap visualization of genes involved in TCR and immune response signatures compared with *FOXP2* microarray transcript expression (probe: 1555516_at) in FOXP2 protein positive or negative GCB-DLBCL (*n* = 24) or ABC-DLBCL (*n* = 15) cases. Line graphs comparing *FOXP2* transcript expression with the averaged expression values of TCR/immune response genes (the region shaded in turquoise represents the standard deviation of the averaged values) were plotted corresponding to each case present on the heatmap.

Gene ontology (GO) analysis was performed to determine the biological pathways associated with FOXP2 protein expression in DLBCL (Figure 6A, Table S2). Analysis of the top 100 genes from all 39 DLBCL patients identified three significantly differentially represented GO terms inversely-associated with the presence of FOXP2 expression: regulation of cellular component organization, clathrin coat assembly and cellular component morphogenesis, with the *DAB2* and *SNAP91* genes being common to all three pathways. Although not represented in these pathways, HIP1R is already known to be functionally involved in clathrin assembly [44]. There were no statistically significant GO terms that distinguished FOXP2-positive versus FOXP2-negative GCB-DLBCL cases, and no GO terms from the upregulated gene lists (*i.e.* positively-associated with FOXP2 expression) that distinguished FOXP2-positive versus FOXP2-negative DLBCL or ABC-DLBCL cases.

Analysis of genes negatively-associated with FOXP2 protein expression in ABC-DLBCL cases identified distinct GO terms and pathways (Figure 6A; Table S2). The biological processes most frequently identified were those associated with T-cell receptor (TCR) signaling, immune responses and cell activation. Individual genes involved in TCR signaling and immune responses were downregulated in ABC-DLBCL with FOXP2 protein expression and showed a significantly inverse relationship with *FOXP2* transcript expression (*P* < 0.0001; Figure 6B). In contrast, in GBC-DLBCL the same genes showed no correlation with either FOXP2 protein or transcript expression, consistent with the absence of immune response and TCR-signaling GO pathways distinguishing FOXP2-positive versus -negative GCB-DLBCL.

## DISCUSSION

There are a number of studies implicating FOXP2 in human malignancy. The first, from our laboratory, described the high frequency expression of FOXP2 in malignant plasma cells in multiple myeloma [43]. Among prostate cancers both loss and strong expression of FOXP2 was reported. Strong FOXP2 expression was associated with increased early risk of PSA (prostate-specific antigen) recurrence in ERG (ets-related gene) fusion-negative tumors [45]. In breast cancer a network of microRNAs (particularly miR-199a), whose expression is deregulated by mesenchymal stem/stromal cells, repress FOXP2 expression. FOXP2 silencing in breast cancer promoted cancer stem cells and tumor metastasis, while elevated miR-199a and reduced FOXP2 expression correlated with a significantly inferior outcome for patients [46]. Downregulation of FOXP2 also correlated with poor survival in hepatocellular carcinoma patients and enhanced cell invasiveness, reducing the expression of E-cadherin and increasing that of vimentin [47]. There are parallels in the central nervous system where Foxp2 and Foxp4 repress N-cadherin to regulate the integrity and cytoarchitecture of neuroepithelial progenitors [48]. Thus like many transcription factors including FOXP1, FOXP2 may function as either an oncogene or tumor suppressor depending on cellular context.

The firmly established importance of FOXP1 in the pathogenesis of high-risk DLBCL and its partially overlapping functions with FOXP2 in normal tissues promoted us to investigate the expression patterns and clinical relevance of FOXP2 expression in DLBCL. Due to our interest in FOXP1 as a future therapeutic target in DLBCL we also aimed to determine whether FOXP2 might function independently of FOXP1 or whether these transcriptional factors might be co-expressed and exhibit potential functional cooperativity.

Analysis of DLBCL cell lines initially suggested that FOXP2 expression was restricted to ABC-DLBCL, with only two of six cell lines studied here (RIVA and SU-DHL-2) and another (OCI-Ly10) from a previous study [43], exhibiting levels of nuclear protein expression comparable to the JJN3 myeloma cell line. However, in primary DLBCL tumors similar frequency and intensity of FOXP2 protein expression was identified in both DLBCL COO subtypes, independently of whether the COO subtypes were defined by immunohistochemical algorithms or gene expression profiling. Despite common mechanisms inactivating BLIMP1 expression in ABC-DLBCL, a higher frequency expression of this transcription factor was still observed in non-GCB DLBCL in this series. The lack of relationship between full length FOXP2 protein expression and either an ABC/non-GCB DLBCL subtype, or BLIMP1 expression, suggests that its expression in DLBCL is unlikely to reflect purely a plasmablastic tumor phenotype.

Here we show that FOXP2 is expressed in a subset of primary DLBCL tumors. Increased frequency of expression (≥ 20% nuclear positivity) or moderate to strong intensity FOXP2 protein expression both significantly correlated with poor OS and PFS in DLBCL patients treated with R-CHOP. FOXP2 expression identified patients with significantly inferior OS in both the GCB and non-GCB DLBCL subgroups and in multivariate analyses FOXP2 was a high-risk factor independently of both IPI and COO subtype. These data identify a novel role for FOXP2 in the pathogenesis of a subset of DLBCL.

There is currently little information regarding the biological roles of FOXP2 in normal B-cell differentiation or during the pathogenesis of hematological malignancies. FOXP2-positivity in DLBCL was associated with a distinct gene expression signature and GO pathways associated with regulation of cellular component organization, clathrin coat assembly and cellular component morphogenesis. Interestingly, the *DAB2* gene, which is common to all three GO pathways, has been reported to be a putative tumor suppressor whose hypermethylation could contribute to activation of Wnt signaling in myeloma [49]. Analysis of the genetic heterogeneity in DLBCL using exome and whole genome sequencing has identified mutations in key biological pathways, including Wnt signaling [50]. Furthermore, FOXP1 has also been demonstrated to promote Wnt pathway signaling and sensitivity to Wnt inhibitors in DLBCL [33].

In FOXP2-positive ABC-DLBCL the downregulation of many biological processes associated with TCR signaling, immune responses and cell activation was particularly evident. These signatures were absent from FOXP2-positive GCB-DLBCL tumors. This suggests that FOXP2 may have overlapping or complimentary functions with FOXP1 in ABC-DLBCL, as FOXP1 silencing in ABC-DLBCL elevated immune response signatures and major histocompatibility class II expression [32, 34]. Supporting evidence for some common functional relationships between FOXP1 and FOXP2 includes expression of the directly FOXP1 regulated gene *HIP1R* [23], which exhibited an inverse relationship with FOXP2 expression in DLBCL at both the transcript and protein level.

While FOXP2 is co-expressed with FOXP1 in DLBCL, these two transcription factors generally show reciprocal patterns of protein expression in normal and malignant B cells. This mirrors findings in the CNS where only a partial overlap of FOXP1/FOXP2 expression is seen [51]. Thus it is possible that in some patients FOXP1/FOXP2 dimers may regulate gene expression while in others FOXP1 or FOXP2 homodimers (or heterodimers with FOXP4) may regulate distinctly different patterns of gene expression. Further variables are provided by the expression of smaller FOXP1 isoforms in DLBCL [52] and our lack of knowledge regarding FOXP2 isoforms in this malignancy. In view of the importance of a blockade of plasma cell development in DLBCL, it is tempting to speculate that FOXP1 and FOXP2 expression patterns may define a particular and possibly transient stage of plasmablastic B-cell differentiation. Further studies are needed to identify patterns of FOXP2 isoform expression in DLBCL, define its contribution to DLBCL pathogenesis and potential functional cooperativity with FOXP1, and thus elucidate the mechanisms by which FOXP2 expression contributes to poor outcome across DLBCL COO subtypes.

## MATERIALS AND METHODS

### Patient samples and cell lines

Reactive tonsils were provided by the Oxford Radcliffe Biobank (Oxford, UK). DLBCL tissue microarrays (TMAs) have been described previously, and comprise duplicate 1.0 mm cores from a series of Danish patients who had been uniformly treated with R-CHOP with curative intent (*n* = 158) [53]. The clinical characteristics for all patients distributed according to either FOXP2 or BLIMP1 expression are provided in Table 1. Informed consent was obtained from all patients in accordance with the Declaration of Helsinki, and local ethical approval was obtained from the Oxfordshire Research Ethics Committee South Central-Oxford B (CO2.162). DLBCL cell lines were sourced as described previously [43, 52] and are regularly immunophenotyped and shown to be mycoplasma free. Cells were maintained in RPMI1640 media (Life Technologies, Paisley, UK) supplemented with 10% fetal calf serum, 2 mM glutamine at 37^°^C and 5% CO_2_.

### *FOXP2* quantitative reverse-transcription polymerase chain reaction (qRT-PCR)

Total RNA extracted from DLBCL cell lines using the RNeasy kit (Qiagen, Crawley, UK) was reverse transcribed using random primers (Promega, Southampton, UK) and Superscript III reverse transcriptase (Life Technologies). Real-time PCR analysis was performed using a Chromo 4 continuous fluorescence detector (MJ Research, Waltham, MA, USA). Express qPCR supermix (Life Technologies) and a *FOXP2* TaqMan pre-verified probe (Hs00362817_m1; Applied Biosystems, Warrington, UK) were used to amplify *FOXP2* transcripts. Relative gene expression was normalized according to the expression of *18S* rRNA (4319413E; Applied Biosystems) using the formula 2^−ΔΔC(t)^.

### FOXP2 Western blotting

Nuclear extracts from DLBCL cell lines were prepared using commercial reagents (Affymetrix, High Wycombe, UK). Proteins were resolved by gradient SDS-PAGE (NuPAGE, Life Technologies) and transferred to an Amersham^™^ Protran Premium 0.45 μm nitrocellulose membrane (GE Healthcare, Chalfont St Giles, UK). The membrane was blocked for 1 h with 5% fat-free milk powder in PBS, before incubation overnight with primary antibody, FOXP2-73A/8 antibody or JC12 (both used at a 1/30 dilution of ‘in house’ hybridoma supernatant). After washing, the membrane was incubated with 1/5000 dilution of horseradish peroxidase conjugated secondary antibody (Dako, Ely, UK). Labeling was detected using enhanced chemiluminescence reagent (GE Healthcare). Blots were re-probed with nucleophosmin (NA24, undiluted ‘in house’ hybridoma supernatant) to confirm adequate sample loading.

### Immunohistochemistry (IHC)

IHC labeling of the DLBCL TMAs for FOXP1, HIP1R, HLA-DRA and COO markers has been described previously [23, 34]. For the FOXP2 and BLIMP1 staining, paraffin-embedded slides were dewaxed prior to antigen retrieval in 50 mM Tris/2 mM EDTA (pH 9). FOXP2 staining was performed by incubation overnight with a 1/1000 dilution of the ‘in house’ FOXP2-73A/8 hybridoma supernatant at 4^°^C. Staining for BLIMP1 was performed for 30 minutes at room temperature using a 1/40 dilution of hybridoma supernatant from the mouse monoclonal anti-BLIMP1 clone ROS195G (Dr. Giovanna Roncador, CNIO, Madrid, Spain). Detection was performed with the NovoLink polymer system according the manufacturer's instructions (Novocastra, Leica Microsystems, Milton Keynes, UK) and detected using an EnVision kit according to the manufacturer's instructions (DakoCytomation, Denmark). Tonsil tissue was used as a positive control for immunolabeling and to confirm BLIMP1 and FOXP2 labeling patterns were consistent with those previously reported. In particular, the absence of FOXP2 expression in normal lymphoid cells confirmed the lack of cross-reactivity with FOXP1.

FOXP2 and BLIMP1 nuclear expression were independently scored by A.H.B/L.L and E.J.S, who were blinded to patient outcome and clinical characteristics. The non-nuclear 73A/8 staining observed in a small number of cases was not scored. A qualitative score was generated using a score of 3 for the strongest intensity of staining with 2 = moderate, 1 = weak and 0 = no labeling; when heterogeneity of intensity was observed within a core the stronger expression was scored, as long as this was present in more than a small minority of cells. A quantitative score was generated for tumor cell positivity in 10% increments. Discrepant scoring was resolved by joint review using a multi-headed microscope. Differences in intensity and frequency between duplicate cores were averaged and rounded up to the nearest intensity score or 10% frequency.

### Co-immunoprecipitation

RIVA cell lysate (100 μg), prepared using RIPA buffer, was incubated separately with 20 μl of anti-FOXP1 (JC12, hybridoma supernatant), 20 μl of anti-NFIL3 (130/B4, “in house” hybridoma supernatant), or 2 μg of non-specific mouse IgG2a isotype (Dako, Ely, UK) antibody at 4^°^C for 2 h on a rotating wheel. 50 μl of μM ACS Protein G MicroBeads (Miltenyi Biotec, Bergisch Gladbach, Germany) was added into each sample and rotated for a further 1 h at 4^°^C. Each lysate was then loaded onto a microcolumn (Miltenyi Biotec) and washed four times with 200 μl RIPA buffer. After one wash with low-salt wash buffer (50 mM NaCl, 20 mM Tris-HCl [pH 7.5], 2 mM EDTA, 1 × PIC, 1xPhos-STOP), 50 μl of pre-heated (95^°^C) 1 × SDS gel loading buffer was added and the eluate was collected in a fresh collection tube. The eluates were analyzed by Western blotting using anti-FOXP1 (JC12) or anti-FOXP2 (73A/8) antibodies as described above. A sample of the original RIVA lysate was included as the ‘input’.

### Microarray data analysis

A total of 39 DLBCL cases derived from the TMA series (*n* = 158) studied here were previously gene expression profiled (GSE31312) [28]. Differentially expressed genes (DEGs), analyzed by using the GEO2R platform available on Gene Expression Omnibus and *P* ≤ 0.01 threshold used to determine significance, were obtained by comparing FOXP2 protein-positive versus FOXP2 protein-negative samples separately within three groups of cases: (1) All DLBCL cases (*n* = 39); (2) GCB-DLBCL (*n* = 24); (3) ABC-DLBCL (*n* = 15). This resulted in two lists of DEGs for each group of cases comprising of up- or downregulated genes in FOXP2-positive versus FOXP2-negative DLBCL (Table S1). The 100 genes exhibiting the most significant difference in expression within each list were used for Gene Ontology (GO) and pathway enrichment analyses using the ToppGene Suite (https://toppgene.cchmc.org/) (Table S2).

### Statistical analysis

Categorical and continuous variables were compared using chi-square and Mann-Whitney *U* tests, respectively, and Pearson correlation was used to determine association between two variables (GraphPad Prism v6.05; La Jolla, CA, USA). Survival analyses were conducted using the Kaplan-Meier method and compared using the log-rank test. Multivariate analysis was performed by comparing IPI ≥ 3 or non-GCB DLBCL subtype group of cases versus the cohort with FOXP2 ≥ 20% expression (SPSS Statistics v22; Chicago, IL, USA). A two-sided *P*-value of <0.05 determined statistical significance in all analyses.

## SUPPLEMENTARY MATERIAL TABLES


