# Supplementary material for: FOXP2-positive diffuse large B-cell lymphomas exhibit a poor response to R-CHOP therapy and distinct biological signatures
Source: Oncotarget. 2016 May 20;7(33):52940–56. doi: 10.18632/oncotarget.9507 (PMC5288160; doi:10.18632/oncotarget.9507)
Supplement: Supplementary file 1 [file oncotarget-07-52940-s001.pdf]

FOXP2-positive diffuse large B-cell lymphomas exhibit a poor response to R-CHOP therapy and distinct biological signatures

Supplementary Materials

Supplementary Table S1: List of differentially expressed genes (DEGs) in FOXP2-positive versus FOXP2-negative samples within three subgroups of cases, all DLBCL cases without regarding subtype (*n* = 39), GCB-DLBCL (*n* = 24) or ABC-DLBCL (*n* = 15)

Upregulated genes (*P* < 0.01) in FOXP2-positive DLCBL cases (irrespective of COO; *n* = 39)

Probe ID	*P*-value	Log (FC)	Gene symbol	Gene name
1555516_at	7.92E–06	0.143	*FOXP2*	forkhead box P2
1552760_at	0.0002	0.148	*HDAC9*	histone deacetylase 9
222134_at	0.0002	0.108	*DDO*	D-aspartate oxidase
212121_at	0.0002	0.166	*TCTN3*	tectonic family member 3
1557128_at	0.0003	0.165	*FAM111B*	family with sequence similarity 111, member B
1562249_at	0.0003	0.18	*EPHA1-AS1*	EPHA1 antisense RNA 1
207689_at	0.0003	0.0926	*TBX10*	T-box 10
224024_at	0.0003	0.114	*ERGIC1*	endoplasmic reticulum-golgi intermediate compartment (ERGIC) 1
214987_at	0.0003	0.144	*GAB1*	GRB2-associated binding protein 1
232110_at	0.0004	0.117	*GALNT5*	polypeptide N-acetylgalactosaminyltransferase 5
211448_s_at	0.0004	0.0813	*RGS6*	regulator of G-protein signaling 6
235647_at	0.0005	0.115	*AP4S1*	adaptor-related protein complex 4, sigma 1 subunit
235055_x_at	0.0005	0.109	*MUC4*	mucin 4, cell surface associated
230803_s_at	0.0005	0.14	*ARHGAP24*	Rho GTPase activating protein 24
221073_s_at	0.0006	0.127	*NOD1*	nucleotide-binding oligomerization domain containing 1
231891_at	0.0006	0.105	*STAMBPL1*	STAM binding protein-like 1
202962_at	0.0006	0.156	*KIF13B*	kinesin family member 13B
238874_at	0.0007	0.1	*LOC100506860*	uncharacterized LOC100506860
1560099_at	0.0007	0.109	*LOC339260*	uncharacterized LOC339260
210191_s_at	0.0007	0.136	*PHTF1*	putative homeodomain transcription factor 1
206648_at	0.0008	0.12	*ZNF571*	zinc finger protein 571
222576_s_at	0.0008	0.104	*AGO1*	argonaute RISC catalytic component 1
227987_at	0.0009	0.0827	*VPS13A*	vacuolar protein sorting 13 homolog A (S. cerevisiae)
207052_at	0.0009	0.0997	*HAVCR1*	hepatitis A virus cellular receptor 1
219141_s_at	0.0010	0.109	*AMBRA1*	autophagy/beclin-1 regulator 1
1552924_a_at	0.0010	0.118	*PITPNM2*	phosphatidylinositol transfer protein, membrane-associated 2
219237_s_at	0.0011	0.0894	*DNAJB14*	DnaJ (Hsp40) homolog, subfamily B, member 14
210260_s_at	0.0012	0.126	*TNFAIP8*	tumor necrosis factor, alpha-induced protein 8
222264_at	0.0012	0.156	*HNRNPUL2*	heterogeneous nuclear ribonucleoprotein U-like 2
1568864_at	0.0013	0.119	*MZF1-AS1*	MZF1 antisense RNA 1
238115_at	0.0013	0.0728	*DNAJC18*	DnaJ (Hsp40) homolog, subfamily C, member 18
230720_at	0.0014	0.0749	*RNF182*	ring finger protein 182
221036_s_at	0.0014	0.119	*APH1B*	APH1B gamma secretase subunit
1570255_s_at	0.0014	0.0936	*ANKRD20A8P*	ankyrin repeat domain 20 family, member A8, pseudogene
231871_at	0.0015	0.106	*GPR180*	G protein-coupled receptor 180
233952_s_at	0.0015	0.105	*ZBTB21*	zinc finger and BTB domain containing 21
230796_at	0.0015	0.0879	*LINC01191*	long intergenic non-protein coding RNA 1191

206281_at	0.0016	0.0716	*ADCYAP1*	adenylate cyclase activating polypeptide 1 (pituitary)
1569780_at	0.0016	0.082	*LOC101928700*	uncharacterized LOC101928700
232163_at	0.0016	0.0879	*WDR19*	WD repeat domain 19
219635_at	0.0017	0.121	*ZNF606*	zinc finger protein 606
226685_at	0.0017	0.0898	*SNTB2*	syntrophin, beta 2 (dystrophin-associated protein A1, 59kDa, basic component 2)
225273_at	0.0018	0.121	*WWC3*	WWC family member 3
226769_at	0.0019	0.124	*FIBIN*	fin bud initiation factor homolog (zebrafish)
226092_at	0.0019	0.11	*MPP5*	membrane protein, palmitoylated 5 (MAGUK p55 subfamily member 5)
207366_at	0.0019	0.0955	*KCNS1*	potassium voltage-gated channel, delayed-rectifier, subfamily S, member 1
1555370_a_at	0.0020	0.0773	*CAMTA1*	calmodulin binding transcription activator 1
206386_at	0.0020	0.0801	*SERPINA7*	serpin peptidase inhibitor, clade A (alpha-1 antiproteinase, antitrypsin), member 7
230425_at	0.0020	0.166	*EPHB1*	EPH receptor B1
1570020_at	0.0022	0.0987	*AACSP1*	acetoacetyl-CoA synthetase pseudogene 1
229082_at	0.0022	0.0859	*CCDC125*	coiled-coil domain containing 125
217389_s_at	0.0024	0.156	*ATF5*	activating transcription factor 5
1559227_s_at	0.0024	0.121	*VHL*	von Hippel-Lindau tumor suppressor, E3 ubiquitin protein ligase
242676_at	0.0024	0.122	*NDUFV2-AS1*	NDUFV2 antisense RNA 1
213073_at	0.0024	0.0715	*ZFYVE26*	zinc finger, FYVE domain containing 26
210685_s_at	0.0024	0.121	*UBE4B*	ubiquitination factor E4B
1556389_at	0.0025	0.136	*CNPY3*	canopy FGF signaling regulator 3
1562460_at	0.0025	0.139	*CNDP2*	CNDP dipeptidase 2 (metallopeptidase M20 family)
229812_at	0.0026	0.0851	*USP48*	ubiquitin specific peptidase 48
226385_s_at	0.0026	0.075	*MALSU1*	mitochondrial assembly of ribosomal large subunit 1
221030_s_at	0.0027	0.124	*ARHGAP24*	Rho GTPase activating protein 24
1564707_x_at	0.0028	0.0831	*GLS2*	glutaminase 2 (liver, mitochondrial)
1552450_a_at	0.0029	0.0907	*DNAJC5G*	DnaJ (Hsp40) homolog, subfamily C, member 5 gamma
204983_s_at	0.0030	0.0634	*GPC4*	glypican 4
214478_at	0.0030	0.0627	*SPP2*	secreted phosphoprotein 2, 24kDa
1552485_at	0.0030	0.131	*LACTB*	lactamase, beta
213362_at	0.0030	0.0758	*PTPRD*	protein tyrosine phosphatase, receptor type, D
225107_at	0.0030	0.105	*HNRNPA2B1*	heterogeneous nuclear ribonucleoprotein A2/B1
224846_at	0.0031	0.125	*SHKBP1*	SH3KBP1 binding protein 1
209692_at	0.0031	0.122	*EYA2*	EYA transcriptional coactivator and phosphatase 2
222773_s_at	0.0031	0.1	*GALNT12*	polypeptide N-acetylgalactosaminyltransferase 12
1555799_at	0.0031	0.158	*FCRL5*	Fc receptor-like 5
206445_s_at	0.0032	0.117	*PRMT1*	protein arginine methyltransferase 1
227988_s_at	0.0033	0.114	*VPS13A*	vacuolar protein sorting 13 homolog A (S. cerevisiae)
218098_at	0.0033	0.0821	*ARFGEF2*	ADP-ribosylation factor guanine nucleotide-exchange factor 2 (brefeldin A-inhibited)
225761_at	0.0034	0.0919	*PAPD4*	PAP associated domain containing 4
224406_s_at	0.0034	0.111	*FCRL5*	Fc receptor-like 5
205228_at	0.0036	0.0694	*RBMS2*	RNA binding motif, single stranded interacting protein 2
207009_at	0.0036	0.0894	*PHOX2B*	paired-like homeobox 2b
238862_at	0.0036	0.0771	*MFSD4*	major facilitator superfamily domain containing 4
238948_at	0.0038	0.0806	*TM9SF1*	transmembrane 9 superfamily member 1
210104_at	0.0040	0.0871	*MED6*	mediator complex subunit 6
217594_at	0.0040	0.0895	*ZCCHC11*	zinc finger, CCHC domain containing 11
210458_s_at	0.0040	0.131	*TANK*	TRAF family member-associated NFKB activator
223422_s_at	0.0041	0.129	*ARHGAP24*	Rho GTPase activating protein 24
230398_at	0.0042	0.077	*TNS4*	tensin 4

239143_x_at	0.0043	0.0862	*RNF138*	ring finger protein 138, E3 ubiquitin protein ligase
208595_s_at	0.0043	0.0936	*MBD1*	methyl-CpG binding domain protein 1
219574_at	0.0043	0.104	*MARCH1*	membrane-associated ring finger (C3HC4) 1, E3 ubiquitin protein ligase
213638_at	0.0043	0.125	*PHACTR1*	phosphatase and actin regulator 1
235526_at	0.0043	0.0978	*SOX6*	SRY (sex determining region Y)-box 6
226630_at	0.0044	0.0758	*MIS18BP1*	MIS18 binding protein 1
235791_x_at	0.0044	0.111	*CHD1*	chromodomain helicase DNA binding protein 1
215891_s_at	0.0045	0.122	*GM2A*	GM2 ganglioside activator
205548_s_at	0.0045	0.0954	*BTG3*	BTG family, member 3
203142_s_at	0.0045	0.0931	*AP3B1*	adaptor-related protein complex 3, beta 1 subunit
1570625_at	0.0045	0.0815	*TCEB3*	transcription elongation factor B (SIII), polypeptide 3 (110kDa, elongin A)
204246_s_at	0.0046	0.0813	*DCTN3*	dynactin 3 (p22)
1556821_x_at	0.0046	0.112	*DLEU2*	deleted in lymphocytic leukemia 2 (non-protein coding)
205052_at	0.0047	0.0957	*AUH*	AU RNA binding protein/enoyl-CoA hydratase
211949_s_at	0.0047	0.0861	*NOLC1*	nucleolar and coiled-body phosphoprotein 1
203621_at	0.0048	0.0504	*NDUFB5*	NADH dehydrogenase (ubiquinone) 1 beta subcomplex, 5, 16kDa
227595_at	0.0048	0.113	*ZMYM6*	zinc finger, MYM-type 6
241833_at	0.0048	0.0818	*PEX5L*	peroxisomal biogenesis factor 5-like
1553266_at	0.0048	0.132	*CNOT6L*	CCR4-NOT transcription complex, subunit 6-like
212691_at	0.0049	0.102	*NUP188*	nucleoporin 188kDa
1561137_s_at	0.0049	0.052	*GYPE*	glycophorin E (MNS blood group)
209609_s_at	0.0050	0.0838	*MRPL9*	mitochondrial ribosomal protein L9
242711_x_at	0.0051	0.0952	*FANCM*	Fanconi anemia, complementation group M
1557615_a_at	0.0051	0.0791	*SLIT1*	slit homolog 1 (Drosophila)
1561877_at	0.0053	0.0716	*LOC100996624*	uncharacterized LOC100996624
220173_at	0.0054	0.106	*CCDC176*	coiled-coil domain containing 176
218563_at	0.0054	0.102	*NDUFA3*	NADH dehydrogenase (ubiquinone) 1 alpha subcomplex, 3, 9kDa
213021_at	0.0054	0.0702	*GOSR1*	golgi SNAP receptor complex member 1
207112_s_at	0.0054	0.113	*GAB1*	GRB2-associated binding protein 1
232422_at	0.0054	0.0887	*GGACT*	gamma-glutamylamine cyclotransferase
229723_at	0.0055	0.117	*TAGAP*	T-cell activation RhoGTPase activating protein
218587_s_at	0.0056	0.0729	*POGLUT1*	protein O-glucosyltransferase 1
221304_at	0.0057	0.0706	*UGT1A7*	UDP glucuronosyltransferase 1 family, polypeptide A7
33646_g_at	0.0058	0.119	*GM2A*	GM2 ganglioside activator
220078_at	0.0058	0.101	*USP48*	ubiquitin specific peptidase 48
225419_at	0.0058	0.0813	*MPLKIP*	M-phase specific PLK1 interacting protein
240413_at	0.0061	0.14	*PYHIN1*	pyrin and HIN domain family, member 1
227431_at	0.0061	0.0859	*APTR*	Alu-mediated CDKN1A/p21 transcriptional regulator (non-protein coding)
1552648_a_at	0.0062	0.095	*TNFRSF10A*	tumor necrosis factor receptor superfamily, member 10a
213016_at	0.0062	0.0983	*BBX*	bobby sox homolog (Drosophila)
225639_at	0.0063	0.0689	*SKAP2*	src kinase associated phosphoprotein 2
207752_x_at	0.0063	0.115	*PRB1*	proline-rich protein BstNI subfamily 1
238684_at	0.0064	0.109	*SETDB2*	SET domain, bifurcated 2
230524_at	0.0065	0.086	*MPI*	mannose phosphate isomerase
1561518_at	0.0066	0.0791	*LOC283914*	uncharacterized LOC283914
221869_at	0.0066	0.0794	*ZNF512B*	zinc finger protein 512B
1554973_a_at	0.0066	0.0732	*ZBTB26*	zinc finger and BTB domain containing 26
212127_at	0.0067	0.0913	*RANGAP1*	Ran GTPase activating protein 1
223599_at	0.0067	0.103	*TRIM6*	tripartite motif containing 6
235621_at	0.0067	0.0906	*FAHD2A*	fumarylacetoacetate hydrolase domain containing 2A
205063_at	0.0067	0.102	*GEMIN2*	gem (nuclear organelle) associated protein 2
222830_at	0.0068	0.0772	*GRHL1*	grainyhead-like 1 (Drosophila)

1557248_at	0.0068	0.103	*ZNF587*	zinc finger protein 587
206206_at	0.0069	0.0933	*CD180*	CD180 molecule
210282_at	0.0069	0.114	*ZMYM2*	zinc finger, MYM-type 2
225097_at	0.0069	0.098	*HIPK2*	homeodomain interacting protein kinase 2
225109_at	0.0070	0.0757	*OGFOD1*	2-oxoglutarate and iron-dependent oxygenase domain containing 1
222890_at	0.0070	0.149	*CCDC113*	coiled-coil domain containing 113
1552515_at	0.0070	0.12	*HIPK1*	homeodomain interacting protein kinase 1
1554329_x_at	0.0071	0.0889	*STXBP4*	syntaxin binding protein 4
1556873_at	0.0071	0.116	*MEX3C*	mex-3 RNA binding family member C
1564467_at	0.0072	0.0695	*FAM161A*	family with sequence similarity 161, member A
203803_at	0.0072	0.0769	*PCYOX1*	prenylcysteine oxidase 1
1557954_at	0.0072	0.0862	*TXLNG*	taxilin gamma
210961_s_at	0.0073	0.0865	*ADRA1D*	adrenoceptor alpha 1D
1556204_a_at	0.0073	0.123	*ZNF814*	zinc finger protein 814
211428_at	0.0074	0.114	*SERPINA1*	serpin peptidase inhibitor, clade A (alpha-1 antiproteinase, antitrypsin), member 1
206307_s_at	0.0074	0.119	*FOXD1*	forkhead box D1
221370_at	0.0074	0.08	*ZNF717*	zinc finger protein 717
213320_at	0.0075	0.111	*PRMT3*	protein arginine methyltransferase 3
227176_at	0.0076	0.117	*SLC2A13*	solute carrier family 2 (facilitated glucose transporter), member 13
1553037_a_at	0.0076	0.0717	*SYN2*	synapsin II
234980_at	0.0077	0.1	*TMEM56*	transmembrane protein 56
206519_x_at	0.0077	0.155	*SIGLEC6*	sialic acid binding Ig-like lectin 6
208237_x_at	0.0077	0.0851	*ADAM22*	ADAM metallopeptidase domain 22
221986_s_at	0.0077	0.106	*KLHL24*	kelch-like family member 24
231520_at	0.0078	0.0913	*SLC35F3*	solute carrier family 35, member F3
204315_s_at	0.0078	0.114	*GTSE1*	G-2 and S-phase expressed 1
212129_at	0.0078	0.0609	*NIPA2*	non imprinted in Prader-Willi/Angelman syndrome 2
224062_x_at	0.0079	0.0786	*KLK4*	kallikrein-related peptidase 4
206708_at	0.0079	0.0828	*FOXN2*	forkhead box N2
217684_at	0.0080	0.123	*TYMS*	thymidylate synthetase
220623_s_at	0.0080	0.107	*TSGA10*	testis specific, 10
201710_at	0.0081	0.1	*MYBL2*	v-myb avian myeloblastosis viral oncogene homolog-like 2
231423_s_at	0.0081	0.0817	*ANKRD16*	ankyrin repeat domain 16
1562309_s_at	0.0081	0.164	*PHF21B*	PHD finger protein 21B
217489_s_at	0.0082	0.0823	*IL6R*	interleukin 6 receptor
203053_at	0.0082	0.0611	*BCAS2*	breast carcinoma amplified sequence 2
229364_at	0.0083	0.0816	*LOC646870*	centrosomal protein 57kDa pseudogene
229433_at	0.0083	0.0912	*RBM26*	RNA binding motif protein 26
235177_at	0.0083	0.0867	*METTL21A*	methyltransferase like 21A
1570394_at	0.0084	0.105	*XRN1*	5′-3′ exoribonuclease 1
208098_at	0.0085	0.0648	*OR12D3*	olfactory receptor, family 12, subfamily D, member 3
228274_at	0.0085	0.075	*SDSL*	serine dehydratase-like
211213_at	0.0086	0.08	*ORC5*	origin recognition complex, subunit 5
226708_at	0.0087	0.061	*NAPRT*	nicotinate phosphoribosyltransferase
226719_at	0.0088	0.0996	*DERL2*	derlin 2
202146_at	0.0088	0.0723	*IFRD1*	interferon-related developmental regulator 1
224192_at	0.0088	0.191	*FCRL2*	Fc receptor-like 2
233864_s_at	0.0089	0.0836	*VPS35*	vacuolar protein sorting 35 homolog (S. cerevisiae)
1567287_at	0.0090	0.0719	*CLDND1*	claudin domain containing 1
218570_at	0.0090	0.0946	*PTPMT1*	protein tyrosine phosphatase, mitochondrial 1
233666_at	0.0091	0.113	*TMEM106B*	transmembrane protein 106B
213304_at	0.0091	0.0819	*FAM179B*	family with sequence similarity 179, member B

1564494_s_at	0.0091	0.0554	*P4HB*	prolyl 4-hydroxylase, beta polypeptide
1566101_at	0.0092	0.0953	*TTLL5*	tubulin tyrosine ligase-like family, member 5
213319_s_at	0.0092	0.0737	*YBX3*	Y box binding protein 3
217979_at	0.0093	0.0866	*TSPAN13*	tetraspanin 13
223431_at	0.0093	0.0782	*BLOC1S4*	biogenesis of lysosomal organelles complex-1, subunit 4, cappuccino
233644_at	0.0094	0.102	*KATNAL2*	katanin p60 subunit A-like 2
1569246_a_at	0.0094	0.0693	*C8orf74*	chromosome 8 open reading frame 74
225042_s_at	0.0095	0.0616	*CSRNP2*	cysteine-serine-rich nuclear protein 2
224707_at	0.0096	0.0651	*CYSTM1*	cysteine-rich transmembrane module containing 1
231647_s_at	0.0096	0.128	*FCRL5*	Fc receptor-like 5
218953_s_at	0.0096	0.0863	*PCYOX1L*	prenylcysteine oxidase 1 like
207219_at	0.0096	0.106	*ZFP69B*	ZFP69 zinc finger protein B
212061_at	0.0096	0.0607	*U2SURP*	U2 snRNP-associated SURP domain containing
239052_at	0.0097	0.0506	*HNRNPD*	heterogeneous nuclear ribonucleoprotein D (AU-rich element RNA binding protein 1, 37kDa)
222579_at	0.0097	0.0848	*UBA5*	ubiquitin-like modifier activating enzyme 5
220042_x_at	0.0098	0.0954	*HIVEP3*	human immunodeficiency virus type I enhancer binding protein 3
1562976_at	0.0098	0.0736	*LOC100289070*	hypothetical protein LOC100289070
217245_at	0.0098	0.063	*DIAPH2-AS1*	DIAPH2 antisense RNA 1
242821_at	0.0098	0.0775	*CCDC171*	coiled-coil domain containing 171
222357_at	0.0098	0.0993	*ZBTB20*	zinc finger and BTB domain containing 20
201625_s_at	0.0098	0.0797	*INSIG1*	insulin induced gene 1
234237_s_at	0.0099	0.0845	*AMBRA1*	autophagy/beclin-1 regulator 1
243278_at	0.0099	0.0766	*FOXP2*	forkhead box P2
219195_at	0.0099	0.0821	*PPARGC1A*	peroxisome proliferator-activated receptor gamma, coactivator 1 alpha
233489_at	0.0099	0.0747	*TMEM43*	transmembrane protein 43
242540_at	0.0099	0.113	*DNHD1*	dynein heavy chain domain 1

Downregulated genes (*P* < 0.01) in FOXP2-positive DLCBL cases (irrespective of COO; *n* = 39)

Probe ID	*P*-value	Log (FC)	Gene symbol	Gene name
216919_at	6.81E-05	−0.113	*TP53I11*	tumor protein p53 inducible protein 11
1569566_at	0.0001	−0.113	*TBC1D1*	TBC1 (tre-2/USP6, BUB2, cdc16) domain family, member 1
37232_at	0.0003	−0.0956	*KIAA0586*	KIAA0586
225185_at	0.0003	−0.138	*MRAS*	muscle RAS oncogene homolog
32625_at	0.0003	−0.142	*NPR1*	natriuretic peptide receptor 1
222224_at	0.0003	−0.101	*NACA2*	nascent polypeptide-associated complex alpha subunit 2
243099_at	0.0004	−0.111	*NFAM1*	NFAT activating protein with ITAM motif 1
203022_at	0.0005	−0.129	*RNASEH2A*	ribonuclease H2, subunit A
232283_at	0.0005	−0.122	*LYSMD1*	LysM, putative peptidoglycan-binding, domain containing 1
230291_s_at	0.0005	−0.145	*NFIB*	nuclear factor I/B
1553992_s_at	0.0006	−0.0822	*NBR2*	neighbor of BRCA1 gene 2 (non-protein coding)
212428_at	0.0007	−0.0966	*KIAA0368*	KIAA0368
208349_at	0.0007	−0.128	*TRPA1*	transient receptor potential cation channel, subfamily A, member 1
209117_at	0.0008	−0.0919	*WBP2*	WW domain binding protein 2
213068_at	0.0008	−0.216	*DPT*	dermatopontin
217656_at	0.0010	−0.115	*SMARCA4*	SWI/SNF related, matrix associated, actin dependent regulator of chromatin, subfamily a, member 4
229624_at	0.0010	−0.0646	*OPA3*	optic atrophy 3 (autosomal recessive, with chorea and spastic paraplegia)
228568_at	0.0011	−0.109	*GCOM1*	GRINL1A complex locus 1
216620_s_at	0.0011	−0.106	*ARHGEF10*	Rho guanine nucleotide exchange factor (GEF) 10
38340_at	0.0011	−0.0997	*HIP1R*	huntingtin interacting protein 1 related
1555780_a_at	0.0011	−0.0878	*RHEB*	Ras homolog enriched in brain
222759_at	0.0012	−0.105	*SUV420H1*	suppressor of variegation 4-20 homolog 1 (Drosophila)
213409_s_at	0.0012	−0.0941	*RHEB*	Ras homolog enriched in brain

1553990_at	0.0012	−0.0643	*BRICD5*	BRICHOS domain containing 5
234312_s_at	0.0013	−0.0941	*ACSS2*	acyl-CoA synthetase short-chain family member 2
233936_s_at	0.0014	−0.119	*GGNBP2*	gametogenetin binding protein 2
201278_at	0.0014	−0.0978	*DAB2*	Dab, mitogen-responsive phosphoprotein, homolog 2 (Drosophila)
206401_s_at	0.0014	−0.102	*MAPT*	microtubule-associated protein tau
235471_at	0.0015	−0.0974	*VSTM4*	V-set and transmembrane domain containing 4
218900_at	0.0015	−0.093	*CNNM4*	cyclin and CBS domain divalent metal cation transport mediator 4
219289_at	0.0016	−0.105	*HEATR3*	HEAT repeat containing 3
216428_x_at	0.0016	−0.148	*KIR3DX1*	killer cell immunoglobulin-like receptor, three domains, X1
212453_at	0.0017	−0.113	*KIAA1279*	KIAA1279
204894_s_at	0.0017	−0.119	*AOC3*	amine oxidase, copper containing 3
229846_s_at	0.0017	−0.0637	*MAPKAP1*	mitogen-activated protein kinase associated protein 1
202359_s_at	0.0017	−0.0883	*SNX19*	sorting nexin 19
227393_at	0.0018	−0.103	*ANO9*	anoctamin 9
230341_x_at	0.0018	−0.119	*ADAMTS10*	ADAM metallopeptidase with thrombospondin type 1 motif, 10
222101_s_at	0.0019	−0.121	*DCHS1*	dachsous cadherin-related 1
44822_s_at	0.0019	−0.147	*MIER2*	mesoderm induction early response 1, family member 2
229734_at	0.0020	−0.102	*MIR4697HG*	MIR4697 host gene (non-protein coding)
242984_at	0.0021	−0.0991	*MKLN1*	muskelin 1, intracellular mediator containing kelch motifs
244519_at	0.0021	−0.123	*ASXL1*	additional sex combs like transcriptional regulator 1
223975_at	0.0021	−0.0829	*TRIM51*	tripartite motif-containing 51
236555_at	0.0022	−0.108	*TRAF3IP2-AS1*	TRAF3IP2 antisense RNA 1
223150_s_at	0.0023	−0.0884	*PTPN23*	protein tyrosine phosphatase, non-receptor type 23
243809_at	0.0023	−0.0957	*HELQ*	helicase, POLQ-like
221406_s_at	0.0024	−0.0848	*SAPCD1*	suppressor APC domain containing 1
209070_s_at	0.0024	−0.152	*RGS5*	regulator of G-protein signaling 5
210130_s_at	0.0026	−0.0809	*TM7SF2*	transmembrane 7 superfamily member 2
218070_s_at	0.0027	−0.107	*GMPPA*	GDP-mannose pyrophosphorylase A
228501_at	0.0027	−0.0839	*GALNT15*	polypeptide N-acetylgalactosaminyltransferase 15
238483_at	0.0027	−0.132	*SSBP2*	single-stranded DNA binding protein 2
225720_at	0.0027	−0.153	*SYNPO2*	synaptopodin 2
240002_at	0.0029	−0.0883	*FAM93B*	family with sequence similarity 93, member B
201559_s_at	0.0030	−0.113	*CLIC4*	chloride intracellular channel 4
241405_at	0.0030	−0.101	*TOB1-AS1*	TOB1 antisense RNA 1
204953_at	0.0031	−0.0902	*SNAP91*	synaptosomal-associated protein, 91kDa
228412_at	0.0031	−0.107	*LOC643072*	uncharacterized LOC643072
1552532_a_at	0.0031	−0.115	*ATP6V1C2*	ATPase, H+ transporting, lysosomal 42kDa, V1 subunit C2
213713_s_at	0.0033	−0.103	*GLB1L2*	galactosidase, beta 1-like 2
207217_s_at	0.0034	−0.088	*NOX1*	NADPH oxidase 1
41553_at	0.0034	−0.096	*OSGIN2*	oxidative stress induced growth inhibitor family member 2
211190_x_at	0.0034	−0.121	*CD84*	CD84 molecule
222692_s_at	0.0035	−0.151	*FNDC3B*	fibronectin type III domain containing 3B
213032_at	0.0035	−0.087	*NFIB*	nuclear factor I/B
1553079_at	0.0036	−0.079	*TRIM40*	tripartite motif containing 40
230175_s_at	0.0036	−0.114	*DCBLD2*	discoidin, CUB and LCCL domain containing 2
224390_s_at	0.0036	−0.0974	*RGS8*	regulator of G-protein signaling 8
210080_x_at	0.0037	−0.0779	*CELA3A*	chymotrypsin-like elastase family, member 3A
227488_at	0.0037	−0.0801	*MIR503*	microRNA 503
240873_x_at	0.0038	−0.104	*DAB2*	Dab, mitogen-responsive phosphoprotein, homolog 2 (Drosophila)
215873_x_at	0.0039	−0.0636	*ABCC10*	ATP-binding cassette, sub-family C (CFTR/MRP), member 10
204321_at	0.0039	−0.129	*NEO1*	neogenin 1
215588_x_at	0.0040	−0.0786	*RIOK3*	RIO kinase 3

228240_at	0.0040	−0.0829	*AGAP1*	ArfGAP with GTPase domain, ankyrin repeat and PH domain 1
221928_at	0.0042	−0.0946	*ACACB*	acetyl-CoA carboxylase beta
203540_at	0.0042	−0.0675	*GFAP*	glial fibrillary acidic protein
219897_at	0.0042	−0.104	*RNF122*	ring finger protein 122
238802_at	0.0042	−0.146	*TYSND1*	trypsin domain containing 1
1554172_a_at	0.0043	−0.0972	*ZMYM3*	zinc finger, MYM-type 3
210053_at	0.0043	−0.115	*TAF5*	TAF5 RNA polymerase II, TATA box binding protein (TBP)-associated factor, 100kDa
238006_at	0.0043	−0.0998	*SIN3A*	SIN3 transcription regulator family member A
231880_at	0.0045	−0.109	*STRIP2*	striatin interacting protein 2
1558914_at	0.0045	−0.108	*DESI2*	desumoylating isopeptidase 2
1554063_at	0.0046	−0.0973	*C8orf76*	chromosome 8 open reading frame 76
205325_at	0.0047	−0.0798	*PHYHIP*	phytanoyl-CoA 2-hydroxylase interacting protein
226984_at	0.0048	−0.0989	*FGD5*	FYVE, RhoGEF and PH domain containing 5
1569739_at	0.0049	−0.152	*LOC221946*	uncharacterized LOC221946
214728_x_at	0.0049	−0.111	*SMARCA4*	SWI/SNF related, matrix associated, actin dependent regulator of chromatin, subfamily a, member 4
1569588_x_at	0.0051	−0.0778	*PIK3C2A*	phosphatidylinositol-4-phosphate 3-kinase, catalytic subunit type 2 alpha
1569777_a_at	0.0051	−0.0792	*ZPLD1*	zona pellucida-like domain containing 1
204497_at	0.0052	−0.0889	*ADCY9*	adenylate cyclase 9
235248_at	0.0053	−0.0781	*BTBD9*	BTB (POZ) domain containing 9
218062_x_at	0.0053	−0.12	*CDC42EP4*	CDC42 effector protein (Rho GTPase binding) 4
220977_x_at	0.0053	−0.0829	*EPB41L5*	erythrocyte membrane protein band 4.1 like 5
224776_at	0.0054	−0.081	*AGPAT6*	1-acylglycerol-3-phosphate O-acyltransferase 6
202967_at	0.0054	−0.0979	*GSTA4*	glutathione S-transferase alpha 4
211945_s_at	0.0054	−0.0496	*ITGB1*	integrin, beta 1 (fibronectin receptor, beta polypeptide, antigen CD29 includes MDF2, MSK12)
223564_s_at	0.0054	−0.0865	*GNB1L*	guanine nucleotide binding protein (G protein), beta polypeptide 1-like
211641_x_at	0.0056	−0.103	*IGHV3-48*	immunoglobulin heavy variable 3-48
226304_at	0.0056	−0.155	*HSPB6*	heat shock protein, alpha-crystallin-related, B6
215073_s_at	0.0056	−0.0975	*NR2F2*	nuclear receptor subfamily 2, group F, member 2
219156_at	0.0057	−0.107	*SYNJ2BP*	synaptojanin 2 binding protein
242469_at	0.0057	−0.0762	*COLCA2*	colorectal cancer associated 2
202500_at	0.0059	−0.0813	*DNAJB2*	DnaJ (Hsp40) homolog, subfamily B, member 2
205707_at	0.0059	−0.112	*IL17RA*	interleukin 17 receptor A
209887_at	0.0061	−0.0867	*SMAD6*	SMAD family member 6
225650_at	0.0061	−0.0806	*SAMD1*	sterile alpha motif domain containing 1
211499_s_at	0.0062	−0.132	*MAPK11*	mitogen-activated protein kinase 11
213792_s_at	0.0062	−0.156	*INSR*	insulin receptor
211981_at	0.0062	−0.119	*COL4A1*	collagen, type IV, alpha 1
1564203_at	0.0063	−0.0791	*LOC147004*	uncharacterized LOC147004
204141_at	0.0063	−0.11	*TUBB2A*	tubulin, beta 2A class IIa
214378_at	0.0063	−0.0723	*TFPI*	tissue factor pathway inhibitor (lipoprotein-associated coagulation inhibitor)
228109_at	0.0064	−0.113	*RASGRF2*	Ras protein-specific guanine nucleotide-releasing factor 2
210678_s_at	0.0064	−0.0763	*AGPAT2*	1-acylglycerol-3-phosphate O-acyltransferase 2
208794_s_at	0.0064	−0.108	*SMARCA4*	SWI/SNF related, matrix associated, actin dependent regulator of chromatin, subfamily a, member 4
200852_x_at	0.0064	−0.0744	*GNB2*	guanine nucleotide binding protein (G protein), beta polypeptide 2
210033_s_at	0.0065	−0.0599	*SPAG6*	sperm associated antigen 6
1560001_at	0.0065	−0.105	*LOC100131581*	uncharacterized LOC100131581
34408_at	0.0065	−0.107	*RTN2*	reticulon 2
212305_s_at	0.0065	−0.113	*MIA3*	melanoma inhibitory activity family, member 3
223601_at	0.0065	−0.0994	*OLFM2*	olfactomedin 2
209736_at	0.0067	−0.0939	*SOX13*	SRY (sex determining region Y)-box 13

207554_x_at	0.0068	−0.0604	*TBXA2R*	thromboxane A2 receptor
226359_at	0.0068	−0.0804	*GTPBP1*	GTP binding protein 1
226940_at	0.0068	−0.0784	*FAM69B*	family with sequence similarity 69, member B
209500_x_at	0.0069	−0.106	*TNFSF12-TNFSF13*	TNFSF12-TNFSF13 readthrough
236340_at	0.0070	−0.0708	*LINC01006*	long intergenic non-protein coding RNA 1006
223435_s_at	0.0070	−0.107	*PCDHA1*	protocadherin alpha 1
212466_at	0.0070	−0.0741	*SPRED2*	sprouty-related, EVH1 domain containing 2
216994_s_at	0.0071	−0.0773	*RUNX2*	runt-related transcription factor 2
235036_at	0.0071	−0.0724	*LIX1L*	Lix1 homolog (chicken) like
1564868_a_at	0.0071	−0.102	*FAM117B*	family with sequence similarity 117, member B
204498_s_at	0.0071	−0.14	*ADCY9*	adenylate cyclase 9
1555131_a_at	0.0072	−0.0649	*PER3*	period circadian clock 3
225422_at	0.0073	−0.077	*CDC26*	cell division cycle 26
223546_x_at	0.0073	−0.0507	*LUC7L*	LUC7-like (S. cerevisiae)
241045_at	0.0075	−0.105	*KDM8*	lysine (K)-specific demethylase 8
208388_at	0.0075	−0.0865	*NR2E3*	nuclear receptor subfamily 2, group E, member 3
1566093_at	0.0076	−0.104	*ARHGEF12*	Rho guanine nucleotide exchange factor (GEF) 12
227329_at	0.0076	−0.0615	*ZBTB46*	zinc finger and BTB domain containing 46
205929_at	0.0077	−0.0641	*GPA33*	glycoprotein A33 (transmembrane)
210750_s_at	0.0078	−0.0901	*DLGAP1*	discs, large (Drosophila) homolog-associated protein 1
215508_at	0.0079	−0.11	*BUB1*	BUB1 mitotic checkpoint serine/threonine kinase
45526_g_at	0.0079	−0.0658	*NAA60*	N(alpha)-acetyltransferase 60, NatF catalytic subunit
1560707_at	0.0079	−0.0655	*LOC283856*	uncharacterized LOC283856
218975_at	0.0080	−0.13	*COL5A3*	collagen, type V, alpha 3
227870_at	0.0080	−0.107	*IGDCC4*	immunoglobulin superfamily, DCC subclass, member 4
241353_s_at	0.0081	−0.0899	*LOC100507507*	uncharacterized LOC100507507
218931_at	0.0082	−0.0853	*RAB17*	RAB17, member RAS oncogene family
235431_s_at	0.0082	−0.112	*PELI3*	pellino E3 ubiquitin protein ligase family member 3
1554983_at	0.0083	−0.0563	*LINC00317*	long intergenic non-protein coding RNA 317
1556472_s_at	0.0083	−0.123	*SCML4*	sex comb on midleg-like 4 (Drosophila)
244834_at	0.0084	−0.11	*C1orf134*	chromosome 1 open reading frame 134
220254_at	0.0085	−0.0705	*LRP12*	low density lipoprotein receptor-related protein 12
224516_s_at	0.0086	−0.0925	*CXXC5*	CXXC finger protein 5
207386_at	0.0086	−0.0819	*CYP7B1*	cytochrome P450, family 7, subfamily B, polypeptide 1
212627_s_at	0.0087	−0.0979	*EXOSC7*	exosome component 7
218173_s_at	0.0088	−0.0996	*WHSC1L1*	Wolf-Hirschhorn syndrome candidate 1-like 1
219337_at	0.0088	−0.0905	*C1orf159*	chromosome 1 open reading frame 159
243292_at	0.0089	−0.0812	*FAM132A*	family with sequence similarity 132, member A
229936_at	0.0089	−0.0739	*GFRA3*	GDNF family receptor alpha 3
232724_at	0.0090	−0.122	*MS4A6A*	membrane-spanning 4-domains, subfamily A, member 6A
214843_s_at	0.0091	−0.0777	*USP33*	ubiquitin specific peptidase 33
48612_at	0.0092	−0.0796	*N4BP1*	NEDD4 binding protein 1
211650_x_at	0.0093	−0.15	*IGHV1-69*	immunoglobulin heavy variable 1-69
219309_at	0.0093	−0.076	*C22orf46*	chromosome 22 open reading frame 46
212645_x_at	0.0093	−0.0588	*BRE*	brain and reproductive organ-expressed (TNFRSF1A modulator)
227400_at	0.0093	−0.0976	*NFIX*	nuclear factor I/X (CCAAT-binding transcription factor)
215216_at	0.0094	−0.0955	*VPS16*	vacuolar protein sorting 16 homolog (S. cerevisiae)
228291_s_at	0.0095	−0.0491	*KIZ*	kizuna centrosomal protein
222037_at	0.0095	−0.0933	*MCM4*	minichromosome maintenance complex component 4
211610_at	0.0095	−0.0738	*KLF6*	Kruppel-like factor 6
203626_s_at	0.0096	−0.0879	*SKP2*	S-phase kinase-associated protein 2, E3 ubiquitin protein ligase

207643_s_at	0.0097	−0.0687	*TNFRSF1A*	tumor necrosis factor receptor superfamily, member 1A
225329_at	0.0098	−0.0585	*FAM195B*	family with sequence similarity 195, member B
1555071_at	0.0098	−0.0615	*TLL1*	tolloid-like 1
1563620_at	0.0098	−0.0672	*BTRC*	beta-transducin repeat containing E3 ubiquitin protein ligase
200645_at	0.0098	−0.0592	*GABARAP*	GABA(A) receptor-associated protein
1561085_at	0.0098	−0.0625	*LOC153910*	uncharacterized LOC153910
226369_at	0.0099	−0.0592	*LINC01089*	long intergenic non-protein coding RNA 1089
221646_s_at	0.0099	−0.153	*ZDHHC11*	zinc finger, DHHC-type containing 11

Upregulated genes ($P < 0.01$) in FOXP2-positive GCB-DLCBL cases ($n = 24$)

Probe ID	*P*-value	Log (FC)	Gene symbol	Gene name
211448_s_at	3.37E-06	0.1432	*RGS6*	regulator of G-protein signaling 6
1552924_a_at	3.56E-06	0.1947	*PITPNM2*	phosphatidylinositol transfer protein, membrane-associated 2
1552760_at	0.0001	0.2111	*HDAC9*	histone deacetylase 9
1555742_at	0.0002	0.1579	*ERVH-6*	endogenous retrovirus group H, member 6
205317_s_at	0.0002	0.1754	*SLC15A2*	solute carrier family 15 (oligopeptide transporter), member 2
1562785_at	0.0003	0.1449	*HERC6*	HECT and RLD domain containing E3 ubiquitin protein ligase family member 6
213697_at	0.0004	0.1390	*HIPK3*	homeodomain interacting protein kinase 3
240834_at	0.0004	0.1639	*OTULIN*	OTU deubiquitinase with linear linkage specificity
219195_at	0.0004	0.1405	*PPARGC1A*	peroxisome proliferator-activated receptor gamma, coactivator 1 alpha
215071_s_at	0.0004	0.1575	*HIST1H2AC*	histone cluster 1, H2ac
1569097_at	0.0006	0.1391	*TP53BP1*	tumor protein p53 binding protein 1
242676_at	0.0006	0.1824	*NDUFV2-AS1*	NDUFV2 antisense RNA 1
239998_at	0.0007	0.1460	*C10orf53*	chromosome 10 open reading frame 53
203231_s_at	0.0007	0.1934	*ATXN1*	ataxin 1
207112_s_at	0.0007	0.1686	*GAB1*	GRB2-associated binding protein 1
215559_at	0.0008	0.1124	*ABCC6*	ATP-binding cassette, sub-family C (CFTR/MRP), member 6
1562249_at	0.0008	0.2162	*EPHA1-AS1*	EPHA1 antisense RNA 1
235228_at	0.0009	0.2504	*CCDC85A*	coiled-coil domain containing 85A
202865_at	0.0009	0.1210	*DNAJB12*	DnaJ (Hsp40) homolog, subfamily B, member 12
207689_at	0.0009	0.1158	*TBX10*	T-box 10
235369_at	0.0010	0.1972	*C14orf28*	chromosome 14 open reading frame 28
210458_s_at	0.0011	0.2016	*TANK*	TRAF family member-associated NFKB activator
224102_at	0.0011	0.1709	*P2RY12*	purinergic receptor P2Y, G-protein coupled, 12
225107_at	0.0012	0.1500	*HNRNPA2B1*	heterogeneous nuclear ribonucleoprotein A2/B1
232425_at	0.0013	0.1817	*SNX25*	sorting nexin 25
235055_x_at	0.0013	0.1287	*MUC4*	mucin 4, cell surface associated
220712_at	0.0014	0.1355	*C8orf60*	chromosome 8 open reading frame 60
211139_s_at	0.0015	0.1091	*NAB1*	NGFI-A binding protein 1 (EGR1 binding protein 1)
1565752_at	0.0016	0.2154	*FGD2*	FYVE, RhoGEF and PH domain containing 2
237565_at	0.0016	0.1566	*GCC2*	GRIP and coiled-coil domain containing 2
213497_at	0.0017	0.1335	*ABTB2*	ankyrin repeat and BTB (POZ) domain containing 2
219868_s_at	0.0018	0.0996	*ANKFY1*	ankyrin repeat and FYVE domain containing 1
219237_s_at	0.0019	0.1154	*DNAJB14*	DnaJ (Hsp40) homolog, subfamily B, member 14
224024_at	0.0020	0.1296	*ERGIC1*	endoplasmic reticulum-golgi intermediate compartment (ERGIC) 1
230242_at	0.0020	0.1417	*NFASC*	neurofascin
223563_at	0.0020	0.1186	*GNB1L*	guanine nucleotide binding protein (G protein), beta polypeptide 1-like
232360_at	0.0020	0.1094	*EHF*	ets homologous factor
222514_at	0.0020	0.1270	*RRAGC*	Ras-related GTP binding C
232110_at	0.0021	0.1455	*GALNT5*	polypeptide N-acetylgalactosaminyltransferase 5
206038_s_at	0.0021	0.1018	*NR2C2*	nuclear receptor subfamily 2, group C, member 2
224650_at	0.0021	0.1282	*MAL2*	mal, T-cell differentiation protein 2 (gene/pseudogene)

229604_at	0.0021	0.1112	*CMAHP*	cytidine monophospho-N-acetylneuraminic acid hydroxylase, pseudogene
1555257_a_at	0.0022	0.1110	*MYO3B*	myosin IIIB
202106_at	0.0022	0.1174	*GOLGA3*	golgin A3
1562238_at	0.0023	0.1590	*USPL1*	ubiquitin specific peptidase like 1
217187_at	0.0023	0.1307	*MUC5AC*	mucin 5AC, oligomeric mucus/gel-forming
205833_s_at	0.0023	0.1335	*PART1*	prostate androgen-regulated transcript 1 (non-protein coding)
1565754_x_at	0.0024	0.1659	*FGD2*	FYVE, RhoGEF and PH domain containing 2
205246_at	0.0024	0.1452	*PEX13*	peroxisomal biogenesis factor 13
236754_at	0.0024	0.1284	*PPP1R2*	protein phosphatase 1, regulatory (inhibitor) subunit 2
219861_at	0.0025	0.1218	*DNAJC17*	DnaJ (Hsp40) homolog, subfamily C, member 17
1562976_at	0.0025	0.1119	*LOC100289070*	hypothetical protein LOC100289070
206445_s_at	0.0026	0.1490	*PRMT1*	protein arginine methyltransferase 1
1569099_at	0.0026	0.2109	*LOC101929450*	uncharacterized LOC101929450
214123_s_at	0.0028	0.1406	*NOP14-AS1*	NOP14 antisense RNA 1
1566776_at	0.0028	0.1277	*DNAH1*	dynein, axonemal, heavy chain 1
222264_at	0.0029	0.2049	*HNRNPUL2*	heterogeneous nuclear ribonucleoprotein U-like 2
243438_at	0.0030	0.1029	*PDE7B*	phosphodiesterase 7B
201729_s_at	0.0033	0.1327	*KIAA0100*	KIAA0100
214478_at	0.0033	0.0900	*SPP2*	secreted phosphoprotein 2, 24kDa
241414_at	0.0034	0.1573	*ANKRD10*	ankyrin repeat domain 10
231836_at	0.0034	0.1407	*HKR1*	HKR1, GLI-Kruppel zinc finger family member
204315_s_at	0.0034	0.1736	*GTSE1*	G-2 and S-phase expressed 1
210260_s_at	0.0034	0.1446	*TNFAIP8*	tumor necrosis factor, alpha-induced protein 8
33646_g_at	0.0034	0.1684	*GM2A*	GM2 ganglioside activator
211949_s_at	0.0034	0.1222	*NOLC1*	nucleolar and coiled-body phosphoprotein 1
232674_at	0.0034	0.1093	*UCN2*	urocortin 2
1553493_a_at	0.0035	0.1322	*TDH*	L-threonine dehydrogenase (pseudogene)
212096_s_at	0.0035	0.1190	*MTUS1*	microtubule associated tumor suppressor 1
202717_s_at	0.0035	0.1463	*CDC16*	cell division cycle 16
232585_at	0.0035	0.1367	*LOC100128729///TLK2*	hypothetical LOC100128729///tousled-like kinase 2
220623_s_at	0.0035	0.1612	*TSGA10*	testis specific, 10
227355_at	0.0036	0.1261	*RBM26*	RNA binding motif protein 26
1562381_at	0.0036	0.1250	*HLA-F-AS1*	HLA-F antisense RNA 1
222038_s_at	0.0037	0.1434	*UTP18*	UTP18 small subunit (SSU) processome component homolog (yeast)
1561218_s_at	0.0037	0.1111	*LOC728099*	uncharacterized LOC728099
226329_s_at	0.0037	0.0878	*MITD1*	MIT, microtubule interacting and transport, domain containing 1
219141_s_at	0.0039	0.1308	*AMBRA1*	autophagy/beclin-1 regulator 1
217964_at	0.0039	0.1419	*TTC19*	tetratricopeptide repeat domain 19
218179_s_at	0.0040	0.1286	*TRAPPC11*	trafficking protein particle complex 11
207752_x_at	0.0041	0.1229	*PRB1*	proline-rich protein BstNI subfamily 1
207191_s_at	0.0041	0.1350	*ISLR*	immunoglobulin superfamily containing leucine-rich repeat
206847_s_at	0.0042	0.2171	*HOXA7*	homeobox A7
222127_s_at	0.0042	0.0818	*EXOC1*	exocyst complex component 1
214703_s_at	0.0042	0.0757	*MAN2B2*	mannosidase, alpha, class 2B, member 2
242056_at	0.0043	0.1277	*TRIM45*	tripartite motif containing 45
223648_s_at	0.0044	0.1146	*FGFRL1*	fibroblast growth factor receptor-like 1
213915_at	0.0044	0.1830	*NKG7*	natural killer cell granule protein 7
223431_at	0.0044	0.1169	*BLOC1S4*	biogenesis of lysosomal organelles complex-1, subunit 4, cappuccino
241437_s_at	0.0044	0.1377	*EP400NL*	EP400 N-terminal like
223438_s_at	0.0045	0.1134	*PPARA*	peroxisome proliferator-activated receptor alpha
210087_s_at	0.0045	0.1694	*MPZL1*	myelin protein zero-like 1
201419_at	0.0046	0.0991	*BAP1*	BRCA1 associated protein-1 (ubiquitin carboxy-terminal hydrolase)

235845_at	0.0046	0.0984	*SP5*	Sp5 transcription factor
206175_x_at	0.0047	0.1029	*ZNF222*	zinc finger protein 222
201384_s_at	0.0047	0.0915	*NBR1*	neighbor of BRCA1 gene 1
231321_s_at	0.0047	0.1143	*ACER3*	alkaline ceramidase 3
238470_at	0.0047	0.1222	*SYS1*	Sys1 golgi trafficking protein
235753_at	0.0048	0.1833	*HOXA7*	homeobox A7
209482_at	0.0048	0.0910	*POP7*	processing of precursor 7, ribonuclease P/MRP subunit (S. cerevisiae)
232306_at	0.0048	0.1179	*CDH26*	cadherin 26
216942_s_at	0.0048	0.1730	*CD58*	CD58 molecule
203713_s_at	0.0048	0.1625	*LLGL2*	lethal giant larvae homolog 2 (Drosophila)
37549_g_at	0.0048	0.1766	*BBS9*	Bardet-Biedl syndrome 9
226140_s_at	0.0049	0.1450	*OTUD1*	OTU deubiquitinase 1
221304_at	0.0050	0.1024	*UGT1A7*	UDP glucuronosyltransferase 1 family, polypeptide A7
233678_at	0.0051	0.1017	*TCF12*	transcription factor 12
212121_at	0.0051	0.1411	*TCTN3*	tectonic family member 3
40016_g_at	0.0051	0.1003	*MAST4*	microtubule associated serine/threonine kinase family member 4
204608_at	0.0051	0.1176	*ASL*	argininosuccinate lyase
206648_at	0.0051	0.1204	*ZNF571*	zinc finger protein 571
1565544_at	0.0052	0.1319	*RNF141*	ring finger protein 141
209508_x_at	0.0053	0.1357	*CFLAR*	CASP8 and FADD-like apoptosis regulator
242917_at	0.0053	0.0926	*RASGEF1A*	RasGEF domain family, member 1A
237478_at	0.0053	0.1086	*LOC399900*	uncharacterized LOC399900
213723_s_at	0.0055	0.1248	*IDUA*	iduronidase, alpha-L-
230720_at	0.0055	0.0876	*RNF182*	ring finger protein 182
239098_at	0.0055	0.1550	*KCNRG*	potassium channel regulator
211188_at	0.0056	0.1145	*CD84*	CD84 molecule
1555564_a_at	0.0056	0.1238	*CFI*	complement factor I
207485_x_at	0.0056	0.1028	*BTN3A1*	butyrophilin, subfamily 3, member A1
229444_at	0.0057	0.1260	*LOC100131607*	uncharacterized LOC100131607
227232_at	0.0060	0.1245	*EVL*	Enah/Vasp-like
1559812_at	0.0060	0.1163	*FAM53B-AS1*	FAM53B antisense RNA 1
241663_at	0.0062	0.0779	*TCAIM*	T cell activation inhibitor, mitochondrial
235791_x_at	0.0062	0.1362	*CHD1*	chromodomain helicase DNA binding protein 1
211876_x_at	0.0062	0.1034	*PCDHGA3*	protocadherin gamma subfamily A, 3
219927_at	0.0063	0.1440	*FCF1*	FCF1 rRNA-processing protein
204600_at	0.0064	0.1071	*EPHB3*	EPH receptor B3
210945_at	0.0064	0.0919	*COL4A6*	collagen, type IV, alpha 6
211076_x_at	0.0064	0.1059	*ATN1*	atrophin 1
221204_s_at	0.0065	0.1196	*CRTAC1*	cartilage acidic protein 1
217997_at	0.0067	0.1422	*PHLDA1*	pleckstrin homology-like domain, family A, member 1
242835_s_at	0.0068	0.1060	*LOC728730*	uncharacterized LOC728730
231891_at	0.0068	0.0959	*STAMBPL1*	STAM binding protein-like 1
1569176_at	0.0068	0.1116	*TMPRSS12*	transmembrane (C-terminal) protease, serine 12
221073_s_at	0.0069	0.1391	*NOD1*	nucleotide-binding oligomerization domain containing 1
226219_at	0.0069	0.0912	*ARHGAP30*	Rho GTPase activating protein 30
202546_at	0.0070	0.1071	*VAMP8*	vesicle-associated membrane protein 8
213406_at	0.0071	0.0767	*WSB1*	WD repeat and SOCS box containing 1
232654_s_at	0.0071	0.0879	*UGT1A6*	UDP glucuronosyltransferase 1 family, polypeptide A6
231027_at	0.0072	0.1243	*LOC730631*	hypothetical LOC730631
1553132_a_at	0.0072	0.2160	*TC2N*	tandem C2 domains, nuclear
218587_s_at	0.0073	0.0967	*POGLUT1*	protein O-glucosyltransferase 1
209692_at	0.0073	0.1508	*EYA2*	EYA transcriptional coactivator and phosphatase 2

221134_at	0.0073	0.1151	*ANGPT4*	angiopoietin 4
239043_at	0.0074	0.1067	*ZNF404*	zinc finger protein 404
205471_s_at	0.0075	0.1071	*DACH1*	dachshund family transcription factor 1
210961_s_at	0.0076	0.1094	*ADRA1D*	adrenoceptor alpha 1D
204194_at	0.0076	0.1279	*BACH1*	BTB and CNC homology 1, basic leucine zipper transcription factor 1
215891_s_at	0.0077	0.1491	*GM2A*	GM2 ganglioside activator
205657_at	0.0077	0.1360	*HAAO*	3-hydroxyanthranilate 3,4-dioxygenase
1557081_at	0.0077	0.0877	*RBM25*	RNA binding motif protein 25
1568905_at	0.0077	0.1018	*LINC01057*	long intergenic non-protein coding RNA 1057
217876_at	0.0078	0.1202	*GTF3C5*	general transcription factor IIIC, polypeptide 5, 63kDa
208948_s_at	0.0078	0.0908	*STAU1*	staufen double-stranded RNA binding protein 1
222890_at	0.0078	0.1696	*CCDC113*	coiled-coil domain containing 113
209370_s_at	0.0079	0.1191	*SH3BP2*	SH3-domain binding protein 2
229414_at	0.0081	0.1063	*PITPNC1*	phosphatidylinositol transfer protein, cytoplasmic 1
208181_at	0.0081	0.1274	*HIST1H4H*	histone cluster 1, H4h
239260_at	0.0081	0.1167	*CORIN*	corin, serine peptidase
205816_at	0.0082	0.1276	*ITGB8*	integrin, beta 8
220292_at	0.0083	0.1096	*ZSCAN32*	zinc finger and SCAN domain containing 32
202792_s_at	0.0083	0.1015	*PPP6R2*	protein phosphatase 6, regulatory subunit 2
232146_at	0.0083	0.1458	*NDUFC1*	NADH dehydrogenase (ubiquinone) 1, subcomplex unknown, 1, 6kDa
213362_at	0.0084	0.0853	*PTPRD*	protein tyrosine phosphatase, receptor type, D
211179_at	0.0084	0.1155	*RUNX1*	runt-related transcription factor 1
230081_at	0.0084	0.0932	*PLCXD3*	phosphatidylinositol-specific phospholipase C, X domain containing 3
206708_at	0.0084	0.1122	*FOXN2*	forkhead box N2
228803_at	0.0085	0.1149	*PMS1*	PMS1 postmeiotic segregation increased 1 (S. cerevisiae)
214881_s_at	0.0085	0.1120	*UBTF*	upstream binding transcription factor, RNA polymerase I
219574_at	0.0087	0.1324	*MARCH1*	membrane-associated ring finger (C3HC4) 1, E3 ubiquitin protein ligase
221036_s_at	0.0087	0.1269	*APH1B*	APH1B gamma secretase subunit
205349_at	0.0088	0.1195	*GNA15*	guanine nucleotide binding protein (G protein), alpha 15 (Gq class)
205186_at	0.0088	0.1048	*DNALI1*	dynein, axonemal, light intermediate chain 1
205422_s_at	0.0088	0.1666	*ITGBL1*	integrin, beta-like 1 (with EGF-like repeat domains)
232551_at	0.0089	0.1007	*SLC26A6*	solute carrier family 26 (anion exchanger), member 6
202182_at	0.0091	0.1563	*KAT2A*	K(lysine) acetyltransferase 2A
205916_at	0.0092	0.1581	*S100A7*	S100 calcium binding protein A7
1557822_at	0.0094	0.0819	*LOC401134*	uncharacterized LOC401134
52731_at	0.0094	0.1031	*AMBRA1*	autophagy/beclin-1 regulator 1
228476_at	0.0094	0.1657	*KIAA1407*	KIAA1407
179_at	0.0094	0.0967	*DTX2P1-UPK3BP1-PMS2P11*	DTX2P1-UPK3BP1-PMS2P11 readthrough transcribed pseudogene
1562103_at	0.0094	0.0940	*JAK1*	Janus kinase 1
208989_s_at	0.0094	0.1213	*KDM2A*	lysine (K)-specific demethylase 2A
236562_at	0.0096	0.1212	*ZNF439*	zinc finger protein 439
206176_at	0.0096	0.1327	*BMP6*	bone morphogenetic protein 6
223278_at	0.0097	0.1984	*GJB2*	gap junction protein, beta 2, 26kDa
201455_s_at	0.0097	0.1124	*NPEPPS*	aminopeptidase puromycin sensitive
219543_at	0.0098	0.0978	*PBLD*	phenazine biosynthesis-like protein domain containing
231216_at	0.0098	0.0986	*TMEM179*	transmembrane protein 179
227244_s_at	0.0098	0.1103	*SSU72*	SSU72 RNA polymerase II CTD phosphatase homolog (S. cerevisiae)
1560854_s_at	0.0099	0.0782	*ZNF107*	zinc finger protein 107
242922_at	0.0099	0.1426	*NOMO3*	NODAL modulator 3
222662_at	0.0099	0.1090	*PPP1R3B*	protein phosphatase 1, regulatory subunit 3B
1564160_at	0.0099	0.1090	*DTHD1*	death domain containing 1
217087_at	0.0099	0.0698	*C1orf68*	chromosome 1 open reading frame 68
1552419_s_at	0.0099	0.0972	*TTLL10*	tubulin tyrosine ligase-like family, member 10

Downregulated genes (*P* < 0.01) in FOXP2-positive GCB-DLCBL cases (*n* = 24)

Probe ID	*P*-value	Log (FC)	Gene symbol	Gene name
214005_at	2.05E–05	−0.1600	*GGCX*	gamma-glutamyl carboxylase
218070_s_at	2.46E–05	−0.1770	*GMPPA*	GDP-mannose pyrophosphorylase A
224516_s_at	3.82E–05	−0.1703	*CXXC5*	CXXC finger protein 5
232283_at	4.16E–05	−0.2062	*LYSMD1*	LysM, putative peptidoglycan-binding, domain containing 1
1553172_at	8.25E–05	−0.1666	*ZNF777*	zinc finger protein 777
209736_at	0.0001	−0.1609	*SOX13*	SRY (sex determining region Y)-box 13
202359_s_at	0.0002	−0.1381	*SNX19*	sorting nexin 19
41553_at	0.0003	−0.1445	*OSGIN2*	oxidative stress induced growth inhibitor family member 2
1553990_at	0.0003	−0.1041	*BRICD5*	BRICHOS domain containing 5
218657_at	0.0003	−0.1571	*RAPGEFL1*	Rap guanine nucleotide exchange factor (GEF)-like 1
235466_s_at	0.0005	−0.1446	*DISP1*	dispatched homolog 1 (Drosophila)
212778_at	0.0005	−0.1206	*PACS2*	phosphofurin acidic cluster sorting protein 2
227340_s_at	0.0005	−0.1586	*RGMB*	repulsive guidance molecule family member b
205034_at	0.0006	−0.1712	*CCNE2*	cyclin E2
211114_x_at	0.0006	−0.1433	*GEMIN2*	gem (nuclear organelle) associated protein 2
214603_at	0.0006	−0.1603	*MAGEA2B///MAGEA2*	melanoma antigen family A, 2B///melanoma antigen family A, 2
204727_at	0.0007	−0.1442	*WDHD1*	WD repeat and HMG-box DNA binding protein 1
230341_x_at	0.0007	−0.1448	*ADAMTS10*	ADAM metallopeptidase with thrombospondin type 1 motif, 10
224029_x_at	0.0007	−0.1304	*SCN11A*	sodium channel, voltage-gated, type XI, alpha subunit
201073_s_at	0.0007	−0.1671	*SMARCC1*	SWI/SNF related, matrix associated, actin dependent regulator of chromatin, subfamily c, member 1
232724_at	0.0008	−0.1827	*MS4A6A*	membrane-spanning 4-domains, subfamily A, member 6A
226661_at	0.0008	−0.1343	*CDCA2*	cell division cycle associated 2
237038_at	0.0009	−0.1176	*CXCL14*	chemokine (C-X-C motif) ligand 14
218408_at	0.0010	−0.1277	*TIMM10*	translocase of inner mitochondrial membrane 10 homolog (yeast)
241367_at	0.0012	−0.1417	*TEX19*	testis expressed 19
1555780_a_at	0.0012	−0.1210	*RHEB*	Ras homolog enriched in brain
203626_s_at	0.0013	−0.1456	*SKP2*	S-phase kinase-associated protein 2, E3 ubiquitin protein ligase
210006_at	0.0013	−0.1428	*ABHD14A*	abhydrolase domain containing 14A
1561540_at	0.0015	−0.1120	*LINC00343*	long intergenic non-protein coding RNA 343
239157_at	0.0015	−0.1329	*ZSCAN12P1*	zinc finger and SCAN domain containing 12 pseudogene 1
1560213_at	0.0015	−0.1309	*HCP5B*	HLA complex P5B (non-protein coding)
1558122_s_at	0.0015	−0.1648	*CSRNP3*	cysteine-serine-rich nuclear protein 3
208716_s_at	0.0015	−0.1411	*TMCO1*	transmembrane and coiled-coil domains 1
228069_at	0.0016	−0.1395	*MTFR2*	mitochondrial fission regulator 2
231271_x_at	0.0016	−0.1098	*NMRAL1*	NmrA-like family domain containing 1
222412_s_at	0.0016	−0.1329	*SSR3*	signal sequence receptor, gamma (translocon-associated protein gamma)
1552299_at	0.0017	−0.1549	*AK9*	adenylate kinase 9
1558914_at	0.0017	−0.1419	*DESI2*	desumoylating isopeptidase 2
203744_at	0.0018	−0.1293	*HMGB3*	high mobility group box 3
211734_s_at	0.0019	−0.0980	*FCER1A*	Fc fragment of IgE, high affinity I, receptor for; alpha polypeptide
231401_s_at	0.0019	−0.1378	*MLX*	MLX, MAX dimerization protein
242984_at	0.0019	−0.1348	*MKLN1*	muskelin 1, intracellular mediator containing kelch motifs
228189_at	0.0020	−0.1332	*BAG4*	BCL2-associated athanogene 4
1552532_a_at	0.0020	−0.1348	*ATP6V1C2*	ATPase, H+ transporting, lysosomal 42kDa, V1 subunit C2
219022_at	0.0021	−0.1663	*C12orf43*	chromosome 12 open reading frame 43

44822_s_at	0.0021	−0.1775	*MIER2*	mesoderm induction early response 1, family member 2
220558_x_at	0.0021	−0.1376	*TSPAN32*	tetraspanin 32
231562_at	0.0022	−0.1631	*APOC2*	apolipoprotein C-II
203072_at	0.0023	−0.1267	*MYO1E*	myosin IE
221990_at	0.0023	−0.1189	*PAX8*	paired box 8
217656_at	0.0023	−0.1345	*SMARCA4*	SWI/SNF related, matrix associated, actin dependent regulator of chromatin, subfamily a, member 4
201980_s_at	0.0024	−0.0994	*RSU1*	Ras suppressor protein 1
205033_s_at	0.0025	−0.1163	*DEFA1B///DEFA3///DEFA1*	defensin, alpha 1B///defensin, alpha 3, neutrophil-specific///defensin, alpha 1
228412_at	0.0025	−0.1390	*LOC643072*	uncharacterized LOC643072
206632_s_at	0.0025	−0.1972	*APOBEC3B*	apolipoprotein B mRNA editing enzyme, catalytic polypeptide-like 3B
212305_s_at	0.0026	−0.1591	*MIA3*	melanoma inhibitory activity family, member 3
217190_x_at	0.0027	−0.1171	*ESR1*	estrogen receptor 1
227154_at	0.0027	−0.1017	*IGSF21*	immunoglobin superfamily, member 21
216428_x_at	0.0027	−0.1719	*KIR3DX1*	killer cell immunoglobulin-like receptor, three domains, X1
37232_at	0.0031	−0.1070	*KIAA0586*	KIAA0586
216620_s_at	0.0031	−0.1196	*ARHGEF10*	Rho guanine nucleotide exchange factor (GEF) 10
230175_s_at	0.0032	−0.1510	*DCBLD2*	discoidin, CUB and LCCL domain containing 2
1554640_at	0.0033	−0.0880	*PALM2*	paralemmin 2
222735_at	0.0033	−0.1390	*TMEM38B*	transmembrane protein 38B
213202_at	0.0034	−0.0962	*SETD1A*	SET domain containing 1A
202926_at	0.0035	−0.1057	*NBAS*	neuroblastoma amplified sequence
1557828_a_at	0.0035	−0.1401	*C5orf28*	chromosome 5 open reading frame 28
210053_at	0.0036	−0.1611	*TAF5*	TAF5 RNA polymerase II, TATA box binding protein (TBP)-associated factor, 100kDa
1553993_s_at	0.0037	−0.0988	*MED25*	mediator complex subunit 25
225696_at	0.0037	−0.1134	*COPS7B*	COP9 signalosome subunit 7B
200712_s_at	0.0037	−0.0960	*MAPRE1*	microtubule-associated protein, RP/EB family, member 1
209003_at	0.0038	−0.1569	*SLC25A11*	solute carrier family 25 (mitochondrial carrier; oxoglutarate carrier), member 11
213409_s_at	0.0039	−0.1295	*RHEB*	Ras homolog enriched in brain
238027_at	0.0039	−0.1312	*SPATA24*	spermatogenesis associated 24
226785_at	0.0039	−0.1261	*ATP11C*	ATPase, class VI, type 11C
34406_at	0.0039	−0.1145	*PACS2*	phosphofurin acidic cluster sorting protein 2
224501_at	0.0039	−0.1481	*PERM1*	PPARGC1 and ESRR induced regulator, muscle 1
216693_x_at	0.0039	−0.1383	*HDGFRP3*	hepatoma-derived growth factor, related protein 3
230283_at	0.0040	−0.1139	*NEURL2*	neuralized E3 ubiquitin protein ligase 2
235517_at	0.0040	−0.1164	*PACRGL*	PARK2 co-regulated-like
237473_at	0.0042	−0.1227	*PPIEL*	peptidylprolyl isomerase E-like pseudogene
217753_s_at	0.0042	−0.1403	*RPS26*	ribosomal protein S26
219012_s_at	0.0044	−0.1415	*C11orf30*	chromosome 11 open reading frame 30
220089_at	0.0044	−0.0820	*L2HGDH*	L-2-hydroxyglutarate dehydrogenase
237523_at	0.0049	−0.1199	*LOC100507371*	hypothetical LOC100507371
233935_at	0.0049	−0.1173	*TNFSF14*	tumor necrosis factor (ligand) superfamily, member 14
240590_at	0.0049	−0.0840	*SPATA3-AS1*	SPATA3 antisense RNA 1 (head to head)
1558976_x_at	0.0050	−0.1020	*UBALD1*	UBA-like domain containing 1
243606_at	0.0050	−0.1563	*NXPE3*	neurexophilin and PC-esterase domain family, member 3
225088_at	0.0050	−0.1463	*FOPNL*	FGFR1OP N-terminal like
230852_at	0.0050	−0.1216	*STAC3*	SH3 and cysteine rich domain 3
201266_at	0.0051	−0.0881	*TXNRD1*	thioredoxin reductase 1
212466_at	0.0052	−0.1070	*SPRED2*	sprouty-related, EVH1 domain containing 2
1558334_a_at	0.0052	−0.1092	*C22orf15*	chromosome 22 open reading frame 15
210716_s_at	0.0053	−0.1076	*CLIP1*	CAP-GLY domain containing linker protein 1

238813_at	0.0054	−0.1209	*LOC100507005///APEX2*	hypothetical LOC100507005///APEX nuclease (apurinic/apyrimidinic endonuclease) 2
211605_s_at	0.0054	−0.0978	*RARA*	retinoic acid receptor, alpha
218582_at	0.0054	−0.1212	*MARCH5*	membrane-associated ring finger (C3HC4) 5
208794_s_at	0.0056	−0.1488	*SMARCA4*	SWI/SNF related, matrix associated, actin dependent regulator of chromatin, subfamily a, member 4
225185_at	0.0056	−0.1569	*MRAS*	muscle RAS oncogene homolog
1557720_s_at	0.0056	−0.1305	*MYO16*	myosin XVI
225731_at	0.0057	−0.1546	*ANKRD50*	ankyrin repeat domain 50
212453_at	0.0057	−0.1318	*KIAA1279*	KIAA1279
208787_at	0.0057	−0.0756	*MRPL3*	mitochondrial ribosomal protein L3
238006_at	0.0058	−0.1230	*SIN3A*	SIN3 transcription regulator family member A
229405_at	0.0058	−0.1029	*KIF7*	kinesin family member 7
219470_x_at	0.0059	−0.0942	*CCNJ*	cyclin J
208658_at	0.0060	−0.0913	*PDIA4*	protein disulfide isomerase family A, member 4
204141_at	0.0061	−0.1459	*TUBB2A*	tubulin, beta 2A class IIa
225172_at	0.0061	−0.1021	*CRAMP1L*	Crm, cramped-like (Drosophila)
218385_at	0.0062	−0.0855	*MRPS18A*	mitochondrial ribosomal protein S18A
236594_at	0.0062	−0.1057	*LLGL1*	lethal giant larvae homolog 1 (Drosophila)
227393_at	0.0063	−0.1222	*ANO9*	anoctamin 9
205325_at	0.0063	−0.1151	*PHYHIP*	phytanoyl-CoA 2-hydroxylase interacting protein
218372_at	0.0063	−0.1195	*MED9*	mediator complex subunit 9
241503_at	0.0063	−0.1126	*FAM81A*	family with sequence similarity 81, member A
205780_at	0.0063	−0.1610	*BIK*	BCL2-interacting killer (apoptosis-inducing)
226025_at	0.0064	−0.1399	*ANKRD28*	ankyrin repeat domain 28
231650_s_at	0.0064	−0.0858	*SEZ6L*	seizure related 6 homolog (mouse)-like
210080_x_at	0.0065	−0.0905	*CELA3A*	chymotrypsin-like elastase family, member 3A
217090_at	0.0066	−0.0937	*ADAM3A*	ADAM metallopeptidase domain 3A (pseudogene)
204953_at	0.0066	−0.0869	*SNAP91*	synaptosomal-associated protein, 91kDa
225684_at	0.0066	−0.0947	*SKA2*	spindle and kinetochore associated complex subunit 2
219726_at	0.0067	−0.1052	*NLGN3*	neuroligin 3
230960_at	0.0067	−0.0898	*IGDCC3*	immunoglobulin superfamily, DCC subclass, member 3
219061_s_at	0.0067	−0.0948	*LAGE3*	L antigen family, member 3
212048_s_at	0.0069	−0.0887	*YARS*	tyrosyl-tRNA synthetase
216601_at	0.0070	−0.1016	*AOC4P*	amine oxidase, copper containing 4, pseudogene
216349_at	0.0070	−0.0955	*FH*	fumarate hydratase
229450_at	0.0070	−0.1206	*IFIT3*	interferon-induced protein with tetratricopeptide repeats 3
231088_at	0.0071	−0.1118	*MORF4L2-AS1*	MORF4L2 antisense RNA 1
225720_at	0.0072	−0.1606	*SYNPO2*	synaptopodin 2
202825_at	0.0073	−0.0957	*SLC25A4*	solute carrier family 25 (mitochondrial carrier; adenine nucleotide translocator), member 4
203240_at	0.0074	−0.1148	*FCGBP*	Fc fragment of IgG binding protein
216919_at	0.0074	−0.1130	*TP53I11*	tumor protein p53 inducible protein 11
1555841_at	0.0074	−0.1093	*MSANTD3*	Myb/SANT-like DNA-binding domain containing 3
225082_at	0.0074	−0.0876	*CPSF3*	cleavage and polyadenylation specific factor 3, 73kDa
1554203_at	0.0076	−0.1184	*GRIK1-AS1*	GRIK1 antisense RNA 1
1558775_s_at	0.0076	−0.0896	*NSMAF*	neutral sphingomyelinase (N-SMase) activation associated factor
229058_at	0.0076	−0.0986	*ANKRD16*	ankyrin repeat domain 16
233954_at	0.0076	−0.1052	*HIATL1*	hippocampus abundant transcript-like 1
204338_s_at	0.0077	−0.1069	*RGS4*	regulator of G-protein signaling 4
1569032_at	0.0077	−0.0794	*LOC642852*	uncharacterized LOC642852
222692_s_at	0.0077	−0.1987	*FNDC3B*	fibronectin type III domain containing 3B
207621_s_at	0.0077	−0.1136	*PEMT*	phosphatidylethanolamine N-methyltransferase
203035_s_at	0.0078	−0.1114	*PIAS3*	protein inhibitor of activated STAT, 3

1561502_x_at	0.0078	−0.0844	*CTU2*	cytosolic thiouridylase subunit 2 homolog (S. pombe)
208848_at	0.0078	−0.1132	*ADH5*	alcohol dehydrogenase 5 (class III), chi polypeptide
219802_at	0.0078	−0.1153	*PYROXD1*	pyridine nucleotide-disulphide oxidoreductase domain 1
218835_at	0.0079	−0.0913	*SFTPA2*	surfactant protein A2
239929_at	0.0079	−0.2040	*PM20D1*	peptidase M20 domain containing 1
224390_s_at	0.0080	−0.1192	*RGS8*	regulator of G-protein signaling 8
221524_s_at	0.0081	−0.1430	*RRAGD*	Ras-related GTP binding D
228595_at	0.0081	−0.1148	*HSD17B1*	hydroxysteroid (17-beta) dehydrogenase 1
212141_at	0.0081	−0.1289	*MCM4*	minichromosome maintenance complex component 4
37462_i_at	0.0082	−0.1017	*SF3A2*	splicing factor 3a, subunit 2, 66kDa
228049_x_at	0.0082	−0.1415	*SNHG19*	small nucleolar RNA host gene 19 (non-protein coding)
216228_s_at	0.0082	−0.1129	*WDHD1*	WD repeat and HMG-box DNA binding protein 1
231772_x_at	0.0083	−0.1022	*CENPH*	centromere protein H
236249_at	0.0083	−0.0991	*IKBIP*	IKBKB interacting protein
228563_at	0.0083	−0.1377	*GJC1*	gap junction protein, gamma 1, 45kDa
210052_s_at	0.0084	−0.0956	*TPX2*	TPX2, microtubule-associated
203282_at	0.0085	−0.0907	*GBE1*	glucan (1,4-alpha-), branching enzyme 1
235527_at	0.0086	−0.1680	*DLGAP1*	discs, large (Drosophila) homolog-associated protein 1
1555100_at	0.0086	−0.0899	*APLF*	aprataxin and PNKP like factor
210290_at	0.0086	−0.1407	*ZNF174*	zinc finger protein 174
225449_at	0.0087	−0.0981	*RDH13*	retinol dehydrogenase 13 (all-trans/9-cis)
1556988_s_at	0.0087	−0.1305	*CHD1L*	chromodomain helicase DNA binding protein 1-like
216479_at	0.0087	−0.1260	*RPL21P28*	ribosomal protein L21 pseudogene 28
212475_at	0.0088	−0.1048	*AVL9*	AVL9 homolog (S. cerevisiase)
1553992_s_at	0.0088	−0.0872	*NBR2*	neighbor of BRCA1 gene 2 (non-protein coding)
234465_at	0.0089	−0.1398	*EME1*	essential meiotic structure-specific endonuclease 1
205359_at	0.0090	−0.1056	*AKAP6*	A kinase (PRKA) anchor protein 6
232103_at	0.0090	−0.1163	*BPNT1*	3’(2’), 5’-bisphosphate nucleotidase 1
241669_x_at	0.0090	−0.0928	*PRKD2*	protein kinase D2
1560370_x_at	0.0091	−0.1593	*ANKH*	ANKH inorganic pyrophosphate transport regulator
202095_s_at	0.0092	−0.1269	*BIRC5*	baculoviral IAP repeat containing 5
216729_at	0.0092	−0.1086	*KLF17P1*	Kruppel-like factor 17 pseudogene 1
219119_at	0.0094	−0.1441	*LSM8*	LSM8 homolog, U6 small nuclear RNA associated (S. cerevisiae)
205929_at	0.0094	−0.0799	*GPA33*	glycoprotein A33 (transmembrane)
228868_x_at	0.0095	−0.1141	*CDT1*	chromatin licensing and DNA replication factor 1
219060_at	0.0095	−0.1252	*WDYHV1*	WDYHV motif containing 1
229306_at	0.0095	−0.1805	*C2CD4B*	C2 calcium-dependent domain containing 4B
213404_s_at	0.0095	−0.1019	*RHEB*	Ras homolog enriched in brain
202348_s_at	0.0096	−0.1124	*TOR1A*	torsin family 1, member A (torsin A)
233955_x_at	0.0096	−0.1267	*CXXC5*	CXXC finger protein 5
231971_at	0.0097	−0.1184	*FANCM*	Fanconi anemia, complementation group M
243927_x_at	0.0097	−0.0899	*KIAA1429*	KIAA1429
236384_at	0.0097	−0.1224	*C17orf85*	chromosome 17 open reading frame 85
229624_at	0.0097	−0.0667	*OPA3*	optic atrophy 3 (autosomal recessive, with chorea and spastic paraplegia)
212627_s_at	0.0098	−0.1316	*EXOSC7*	exosome component 7
229105_at	0.0098	−0.0991	*GPR39*	G protein-coupled receptor 39
221152_at	0.0099	−0.1082	*COL8A1*	collagen, type VIII, alpha 1
220296_at	0.0099	−0.1220	*SAP30L-AS1*	SAP30L antisense RNA 1 (head to head)
212927_at	0.0099	−0.1356	*SMC5*	structural maintenance of chromosomes 5
213718_at	0.0099	−0.1073	*RBM4*	RNA binding motif protein 4
210813_s_at	0.0099	−0.1195	*XRCC4*	X-ray repair complementing defective repair in Chinese hamster cells 4

Upregulated genes ($P < 0.01$) in FOXP2-positive ABC-DLCBL cases ($n = 15$)

Probe ID	*P*-value	Log (FC)	Gene symbol	Gene name
1555516_at	3.51E-05	0.1807	*FOXP2*	forkhead box P2
230914_at	5.61E-05	0.1478	*HNF4A*	hepatocyte nuclear factor 4, alpha
230425_at	0.0001	0.2927	*EPHB1*	EPH receptor B1
1564707_x_at	0.0001	0.1823	*GLS2*	glutaminase 2 (liver, mitochondrial)
1557128_at	0.0001	0.2725	*FAM111B*	family with sequence similarity 111, member B
230802_at	0.0001	0.2525	*ARHGAP24*	Rho GTPase activating protein 24
227595_at	0.0002	0.2196	*ZMYM6*	zinc finger, MYM-type 6
220721_at	0.0002	0.1929	*ZNF614*	zinc finger protein 614
213319_s_at	0.0002	0.1697	*YBX3*	Y box binding protein 3
1553810_a_at	0.0003	0.1830	*KIAA1524*	KIAA1524
206413_s_at	0.0003	0.3103	*TCL1B*	T-cell leukemia/lymphoma 1B
222163_s_at	0.0003	0.1944	*SPATA5L1*	spermatogenesis associated 5-like 1
213320_at	0.0004	0.2306	*PRMT3*	protein arginine methyltransferase 3
210715_s_at	0.0005	0.1356	*SPINT2*	serine peptidase inhibitor, Kunitz type, 2
1556316_s_at	0.0005	0.1326	*MIF-AS1*	MIF antisense RNA 1
206520_x_at	0.0006	0.2640	*SIGLEC6*	sialic acid binding Ig-like lectin 6
235647_at	0.0006	0.1594	*AP4S1*	adaptor-related protein complex 4, sigma 1 subunit
207366_at	0.0007	0.1512	*KCNS1*	potassium voltage-gated channel, delayed-rectifier, subfamily S, member 1
209237_s_at	0.0007	0.1529	*SLC23A2*	solute carrier family 23 (ascorbic acid transporter), member 2
222053_at	0.0007	0.1398	*TAF6L*	TAF6-like RNA polymerase II, p300/CBP-associated factor (PCAF)-associated factor, 65kDa
240048_at	0.0007	0.1178	*STRC*	stereocilin
213959_s_at	0.0007	0.1308	*RPGRIP1L*	RPGRIP1-like
1568864_at	0.0007	0.1622	*MZF1-AS1*	MZF1 antisense RNA 1
240770_at	0.0007	0.1868	*TMEM171*	transmembrane protein 171
240053_x_at	0.0008	0.1542	*PEX5L*	peroxisomal biogenesis factor 5-like
227506_at	0.0009	0.1198	*SLC16A9*	solute carrier family 16, member 9
208927_at	0.0009	0.1282	*SPOP*	speckle-type POZ protein
220025_at	0.0009	0.1317	*TBR1*	T-box, brain, 1
235122_at	0.0009	0.2160	*HIVEP3*	human immunodeficiency virus type I enhancer binding protein 3
1564253_at	0.0009	0.2403	*LOC285766*	uncharacterized LOC285766
231870_s_at	0.0009	0.1211	*NMD3*	NMD3 ribosome export adaptor
1559227_s_at	0.0009	0.1840	*VHL*	von Hippel-Lindau tumor suppressor, E3 ubiquitin protein ligase
222825_at	0.0010	0.1308	*OTUD6B*	OTU domain containing 6B
202541_at	0.0010	0.1933	*AIMP1*	aminoacyl tRNA synthetase complex-interacting multifunctional protein 1
221908_at	0.0010	0.2344	*RNFT2*	ring finger protein, transmembrane 2
231570_at	0.0010	0.1183	*FAM154A*	family with sequence similarity 154, member A
207746_at	0.0010	0.1576	*POLQ*	polymerase (DNA directed), theta
223422_s_at	0.0010	0.1689	*ARHGAP24*	Rho GTPase activating protein 24
235106_at	0.0011	0.1774	*MAML2*	mastermind-like 2 (Drosophila)
204720_s_at	0.0011	0.1905	*DNAJC6*	DnaJ (Hsp40) homolog, subfamily C, member 6
205660_at	0.0011	0.1190	*OASL*	2'-5'-oligoadenylate synthetase-like
204998_s_at	0.0011	0.1339	*ATF5*	activating transcription factor 5
223824_at	0.0012	0.1330	*RNLS*	renalase, FAD-dependent amine oxidase
233156_at	0.0012	0.1645	*RNASEH2B*	ribonuclease H2, subunit B
217901_at	0.0013	0.2165	*DSG2*	desmoglein 2
233633_at	0.0014	0.1690	*TBL1XR1*	transducin (beta)-like 1 X-linked receptor 1
244191_at	0.0014	0.1766	*RPLP1*	ribosomal protein, large, P1
217508_s_at	0.0015	0.1917	*C18orf25*	chromosome 18 open reading frame 25
238547_at	0.0015	0.1309	*HEXIM2*	hexamethylene bis-acetamide inducible 2

240441_at	0.0015	0.1128	*FAM99B*	family with sequence similarity 99, member B (non-protein coding)
203894_at	0.0015	0.1529	*TUBG2*	tubulin, gamma 2
1560527_at	0.0015	0.1288	*NFE4*	nuclear factor, erythroid 4
236835_at	0.0015	0.1717	*FUT8-AS1*	FUT8 antisense RNA 1
232140_at	0.0016	0.1234	*LOC100132352*	FSHD region gene 1 pseudogene
213447_at	0.0016	0.1601	*IPW*	imprinted in Prader-Willi syndrome (non-protein coding)
1556389_at	0.0016	0.2062	*CNPY3*	canopy FGF signaling regulator 3
202606_s_at	0.0016	0.1604	*TLK1*	tousled-like kinase 1
227176_at	0.0016	0.2024	*SLC2A13*	solute carrier family 2 (facilitated glucose transporter), member 13
204120_s_at	0.0016	0.1823	*ADK*	adenosine kinase
204159_at	0.0016	0.1748	*CDKN2C*	cyclin-dependent kinase inhibitor 2C (p18, inhibits CDK4)
1569555_at	0.0016	0.1251	*GDA*	guanine deaminase
229082_at	0.0017	0.1307	*CCDC125*	coiled-coil domain containing 125
235545_at	0.0017	0.1507	*DEPDC1*	DEP domain containing 1
202962_at	0.0017	0.1981	*KIF13B*	kinesin family member 13B
205606_at	0.0018	0.1447	*LRP6*	low density lipoprotein receptor-related protein 6
212654_at	0.0018	0.1815	*TPM2*	tropomyosin 2 (beta)
228280_at	0.0019	0.1474	*ZC3HAV1L*	zinc finger CCCH-type, antiviral 1-like
221123_x_at	0.0019	0.1183	*ZNF395*	zinc finger protein 395
229072_at	0.0019	0.2171	*RAB30*	RAB30, member RAS oncogene family
1554839_at	0.0019	0.1327	*CIDEC*	cell death-inducing DFFA-like effector c
216905_s_at	0.0019	0.2525	*ST14*	suppression of tumorigenicity 14 (colon carcinoma)
1563796_s_at	0.0019	0.1629	*EARS2*	glutamyl-tRNA synthetase 2, mitochondrial
236265_at	0.0019	0.1033	*SP4*	Sp4 transcription factor
222134_at	0.0019	0.1400	*DDO*	D-aspartate oxidase
202144_s_at	0.0019	0.1621	*ADSL*	adenylosuccinate lyase
210614_at	0.0020	0.1165	*TTPA*	tocopherol (alpha) transfer protein
219192_at	0.0020	0.1292	*UBAP2*	ubiquitin associated protein 2
218826_at	0.0020	0.1341	*SLC35F2*	solute carrier family 35, member F2
1552648_a_at	0.0020	0.1390	*TNFRSF10A*	tumor necrosis factor receptor superfamily, member 10a
206519_x_at	0.0020	0.2878	*SIGLEC6*	sialic acid binding Ig-like lectin 6
205704_s_at	0.0021	0.1177	*ATP6V0A2*	ATPase, H+ transporting, lysosomal V0 subunit a2
1558626_at	0.0021	0.1528	*LOC100287696*	similar to ring finger protein 181
203820_s_at	0.0021	0.1987	*IGF2BP3*	insulin-like growth factor 2 mRNA binding protein 3
230325_at	0.0021	0.1148	*LOC100133985*	uncharacterized LOC100133985
212714_at	0.0021	0.1582	*LARP4*	La ribonucleoprotein domain family, member 4
226887_at	0.0021	0.1526	*HSPA14*	heat shock 70kDa protein 14
204146_at	0.0021	0.1917	*RAD51AP1*	RAD51 associated protein 1
1556204_a_at	0.0022	0.2217	*ZNF814*	zinc finger protein 814
209838_at	0.0022	0.1217	*COPS2*	COP9 signalosome subunit 2
228661_s_at	0.0022	0.1596	*LOC102606465*	uncharterized LOC102606465
213482_at	0.0022	0.1416	*DOCK3*	dedicator of cytokinesis 3
207052_at	0.0023	0.1516	*HAVCR1*	hepatitis A virus cellular receptor 1
213877_x_at	0.0023	0.1037	*TCEB2*	transcription elongation factor B (SIII), polypeptide 2 (18kDa, elongin B)
221386_at	0.0023	0.1411	*OR3A2*	olfactory receptor, family 3, subfamily A, member 2
244623_at	0.0023	0.2040	*KCNQ5*	potassium voltage-gated channel, KQT-like subfamily, member 5
222705_s_at	0.0024	0.1735	*SLC25A15*	solute carrier family 25 (mitochondrial carrier; ornithine transporter) member 15
229181_s_at	0.0024	0.1962	*HAUS2*	HAUS augmin-like complex, subunit 2
222889_at	0.0025	0.1322	*DCLRE1B*	DNA cross-link repair 1B
214597_at	0.0025	0.1447	*SSTR2*	somatostatin receptor 2
205780_at	0.0025	0.2173	*BIK*	BCL2-interacting killer (apoptosis-inducing)
222623_s_at	0.0026	0.1162	*ZNF639*	zinc finger protein 639

214718_at	0.0026	0.1246	*GATAD1*	GATA zinc finger domain containing 1
239157_at	0.0026	0.1355	*ZSCAN12P1*	zinc finger and SCAN domain containing 12 pseudogene 1
1552695_a_at	0.0027	0.1733	*SLC2A13*	solute carrier family 2 (facilitated glucose transporter), member 13
219588_s_at	0.0027	0.1671	*NCAPG2*	non-SMC condensin II complex, subunit G2
1564360_a_at	0.0027	0.2820	*LOC339260*	uncharacterized LOC339260
223198_x_at	0.0027	0.1095	*COMMD5*	COMM domain containing 5
221030_s_at	0.0028	0.1450	*ARHGAP24*	Rho GTPase activating protein 24
242711_x_at	0.0028	0.1353	*FANCM*	Fanconi anemia, complementation group M
202843_at	0.0028	0.1698	*DNAJB9*	DnaJ (Hsp40) homolog, subfamily B, member 9
243194_at	0.0029	0.1206	*ZNF551*	zinc finger protein 551
204812_at	0.0029	0.0974	*ZW10*	zw10 kinetochore protein
242372_s_at	0.0029	0.1488	*MFSD4*	major facilitator superfamily domain containing 4
203017_s_at	0.0029	0.1637	*SSX2IP*	synovial sarcoma, X breakpoint 2 interacting protein
217389_s_at	0.0030	0.1959	*ATF5*	activating transcription factor 5
208595_s_at	0.0031	0.1239	*MBD1*	methyl-CpG binding domain protein 1
231213_at	0.0031	0.1328	*PDE1A*	phosphodiesterase 1A, calmodulin-dependent
221820_s_at	0.0031	0.1295	*KAT8*	K(lysine) acetyltransferase 8
241833_at	0.0031	0.1384	*PEX5L*	peroxisomal biogenesis factor 5-like
216233_at	0.0032	0.1463	*CD163*	CD163 molecule
1566720_at	0.0032	0.1036	*RPS10P7*	ribosomal protein S10 pseudogene 7
214376_at	0.0032	0.1343	*MAPK10*	mitogen-activated protein kinase 10
233952_s_at	0.0032	0.1424	*ZBTB21*	zinc finger and BTB domain containing 21
202413_s_at	0.0032	0.1206	*USP1*	ubiquitin specific peptidase 1
226769_at	0.0032	0.2058	*FIBIN*	fin bud initiation factor homolog (zebrafish)
204407_at	0.0032	0.1455	*TTF2*	transcription termination factor, RNA polymerase II
231820_x_at	0.0032	0.1728	*ZNF587*	zinc finger protein 587
228323_at	0.0033	0.1568	*CASC5*	cancer susceptibility candidate 5
205865_at	0.0033	0.1489	*ARID3A*	AT rich interactive domain 3A (BRIGHT-like)
215156_at	0.0033	0.1418	*WDR61*	WD repeat domain 61
221568_s_at	0.0033	0.1495	*LIN7C*	lin-7 homolog C (C. elegans)
211603_s_at	0.0033	0.1288	*ETV4*	ets variant 4
238990_x_at	0.0033	0.1421	*TRIM61*	tripartite motif containing 61
1555910_at	0.0034	0.1392	*PTCD2*	pentatricopeptide repeat domain 2
223514_at	0.0034	0.1398	*CARD11*	caspase recruitment domain family, member 11
205548_s_at	0.0034	0.1399	*BTG3*	BTG family, member 3
230986_at	0.0035	0.1604	*KLF8*	Kruppel-like factor 8
1554901_at	0.0035	0.1586	*GAFA1*	FGF2-associated protein GAFA1
215081_at	0.0036	0.1458	*KIAA1024*	KIAA1024
1553726_s_at	0.0036	0.1141	*TBC1D32*	TBC1 domain family, member 32
211212_s_at	0.0036	0.1470	*ORC5*	origin recognition complex, subunit 5
213610_s_at	0.0037	0.2121	*KLHL23*	kelch-like family member 23
212691_at	0.0038	0.1228	*NUP188*	nucleoporin 188kDa
222958_s_at	0.0038	0.1795	*DEPDC1*	DEP domain containing 1
1553089_a_at	0.0039	0.1042	*WFDC2*	WAP four-disulfide core domain 2
224993_at	0.0039	0.1174	*MLLT1*	myeloid/lymphoid or mixed-lineage leukemia (trithorax homolog, Drosophila); translocated to, 1
230388_s_at	0.0040	0.1701	*KANSL1-AS1*	KANSL1 antisense RNA 1
226092_at	0.0040	0.1696	*MPP5*	membrane protein, palmitoylated 5 (MAGUK p55 subfamily member 5)
229506_at	0.0040	0.1171	*PPM1L*	protein phosphatase, Mg2+/Mn2+ dependent, 1L
233946_at	0.0040	0.1151	*SMU1*	smu-1 suppressor of mec-8 and unc-52 homolog (C. elegans)
220520_s_at	0.0041	0.1432	*NUP62CL*	nucleoporin 62kDa C-terminal like
220651_s_at	0.0041	0.1806	*MCM10*	minichromosome maintenance complex component 10

228988_at	0.0041	0.1895	*ZNF711*	zinc finger protein 711
225526_at	0.0041	0.1586	*MKLN1*	muskelin 1, intracellular mediator containing kelch motifs
217971_at	0.0041	0.1362	*LAMTOR3*	late endosomal/lysosomal adaptor, MAPK and MTOR activator 3
243683_at	0.0041	0.1778	*MORF4L2*	mortality factor 4 like 2
1556477_a_at	0.0042	0.1091	*LOC283485*	uncharacterized LOC283485
1565162_s_at	0.0042	0.1300	*MGST1*	microsomal glutathione S-transferase 1
1567244_at	0.0042	0.0992	*OR5J2*	olfactory receptor, family 5, subfamily J, member 2
243048_at	0.0042	0.2032	*CECR7*	cat eye syndrome chromosome region, candidate 7 (non-protein coding)
217612_at	0.0042	0.1713	*TIMM50*	translocase of inner mitochondrial membrane 50 homolog (S. cerevisiae)
202502_at	0.0042	0.1012	*ACADM*	acyl-CoA dehydrogenase, C-4 to C-12 straight chain
226686_at	0.0042	0.1149	*CISD2*	CDGSH iron sulfur domain 2
204005_s_at	0.0043	0.1210	*PAWR*	PRKC, apoptosis, WT1, regulator
232132_at	0.0043	0.1171	*PARD6G*	par-6 family cell polarity regulator gamma
223389_s_at	0.0043	0.1405	*ZNF581*	zinc finger protein 581
219735_s_at	0.0043	0.1285	*TFCP2L1*	transcription factor CP2-like 1
1557836_at	0.0044	0.2112	*ELMOD2*	ELMO/CED-12 domain containing 2
1555421_at	0.0044	0.1324	*AP1S3*	adaptor-related protein complex 1, sigma 3 subunit
219942_at	0.0044	0.1221	*MYL7*	myosin, light chain 7, regulatory
228565_at	0.0044	0.2133	*KIAA1804*	mixed lineage kinase 4
236939_at	0.0045	0.1359	*PTPLAD2*	protein tyrosine phosphatase-like A domain containing 2
235953_at	0.0045	0.1710	*ZNF610*	zinc finger protein 610
230844_at	0.0045	0.1511	*LOC440934*	uncharacterized LOC440934
231694_at	0.0046	0.1320	*APOA1*	apolipoprotein A-I
231399_at	0.0047	0.1051	*RAB3IP*	RAB3A interacting protein
207106_s_at	0.0047	0.1472	*LTK*	leukocyte receptor tyrosine kinase
204983_s_at	0.0048	0.1061	*GPC4*	glypican 4
241426_at	0.0048	0.1657	*CEP44*	centrosomal protein 44kDa
37943_at	0.0049	0.1101	*ZFYVE26*	zinc finger, FYVE domain containing 26
220334_at	0.0049	0.1252	*RGS17*	regulator of G-protein signaling 17
234968_at	0.0049	0.1401	*DENND4C*	DENN/MADD domain containing 4C
240235_at	0.0050	0.0929	*C10orf62*	chromosome 10 open reading frame 62
200832_s_at	0.0050	0.1627	*SCD*	stearoyl-CoA desaturase (delta-9-desaturase)
214210_at	0.0050	0.1470	*SLC25A17*	solute carrier family 25 (mitochondrial carrier; peroxisomal membrane protein, 34kDa), member 17
226630_at	0.0051	0.1190	*MIS18BP1*	MIS18 binding protein 1
219073_s_at	0.0051	0.1481	*OSBPL10*	oxysterol binding protein-like 10
202314_at	0.0051	0.1398	*CYP51A1*	cytochrome P450, family 51, subfamily A, polypeptide 1
205063_at	0.0052	0.1693	*GEMIN2*	gem (nuclear organelle) associated protein 2
1554242_a_at	0.0052	0.1396	*COCH*	cochlin
221940_at	0.0052	0.1373	*RPUSD2*	RNA pseudouridylate synthase domain containing 2
201626_at	0.0053	0.1128	*INSIG1*	insulin induced gene 1
1555391_a_at	0.0053	0.1006	*CARF*	calcium responsive transcription factor
219276_x_at	0.0054	0.1458	*CAAP1*	caspase activity and apoptosis inhibitor 1
242727_at	0.0055	0.1398	*ARL5B*	ADP-ribosylation factor-like 5B
217539_at	0.0055	0.1352	*C18orf25*	chromosome 18 open reading frame 25
227220_at	0.0056	0.1788	*NFXL1*	nuclear transcription factor, X-box binding-like 1
219178_at	0.0056	0.1043	*QTRTD1*	queuine tRNA-ribosyltransferase domain containing 1
222685_at	0.0056	0.1847	*HAUS6*	HAUS augmin-like complex, subunit 6
210437_at	0.0056	0.3113	*MAGEA9B///MAGEA9*	melanoma antigen family A, 9B///melanoma antigen family A, 9
213223_at	0.0057	0.1301	*RPL28*	ribosomal protein L28
204674_at	0.0057	0.1482	*LRMP*	lymphoid-restricted membrane protein

239337_at	0.0057	0.1695	*LOC400768*	uncharacterized LOC400768
242668_x_at	0.0058	0.1190	*SUN5*	Sad1 and UNC84 domain containing 5
230803_s_at	0.0058	0.1659	*ARHGAP24*	Rho GTPase activating protein 24
218979_at	0.0058	0.2197	*RMI1*	RecQ mediated genome instability 1
1566101_at	0.0058	0.1403	*TTLL5*	tubulin tyrosine ligase-like family, member 5
1557303_at	0.0058	0.1140	*NT5C*	5’, 3’-nucleotidase, cytosolic
228250_at	0.0058	0.1464	*FNIP1*	folliculin interacting protein 1
202146_at	0.0058	0.1079	*IFRD1*	interferon-related developmental regulator 1
201385_at	0.0059	0.0994	*DHX15*	DEAH (Asp-Glu-Ala-His) box helicase 15
222517_at	0.0059	0.1634	*AP3M1*	adaptor-related protein complex 3, mu 1 subunit
1569911_at	0.0059	0.1149	*LINC00824*	long intergenic non-protein coding RNA 824
227377_at	0.0060	0.2130	*IGF2BP1*	insulin-like growth factor 2 mRNA binding protein 1
215017_s_at	0.0060	0.1060	*FNBP1L*	formin binding protein 1-like
214601_at	0.0060	0.1356	*TPH1*	tryptophan hydroxylase 1
231520_at	0.0061	0.1360	*SLC35F3*	solute carrier family 35, member F3
219087_at	0.0061	0.1232	*ASPN*	asporin
213638_at	0.0061	0.1630	*PHACTR1*	phosphatase and actin regulator 1
1560300_a_at	0.0061	0.1103	*DMRTC1B///DMRTC1*	DMRT-like family C1B///DMRT-like family C1
233910_at	0.0062	0.1115	*TMEFF2*	transmembrane protein with EGF-like and two follistatin-like domains 2
1568777_at	0.0062	0.1235	*EML5*	echinoderm microtubule associated protein like 5
230521_at	0.0062	0.1391	*ARHGEF39*	Rho guanine nucleotide exchange factor (GEF) 39
213312_at	0.0062	0.1576	*SMIM8*	small integral membrane protein 8
228652_at	0.0062	0.1461	*ZNF776*	zinc finger protein 776
233314_at	0.0063	0.1394	*PTEN*	phosphatase and tensin homolog
237725_x_at	0.0063	0.0980	*SMC5*	structural maintenance of chromosomes 5
218430_s_at	0.0064	0.1287	*RFX7*	regulatory factor X, 7
1564909_at	0.0064	0.0967	*LOC101928495*	uncharacterized LOC101928495
229232_at	0.0064	0.1707	*LRRC57*	leucine rich repeat containing 57
235736_at	0.0064	0.1833	*SMKR1*	small lysine-rich protein 1
217431_x_at	0.0064	0.1138	*CYBB*	cytochrome b-245, beta polypeptide
222576_s_at	0.0065	0.1291	*AGO1*	argonaute RISC catalytic component 1
221245_s_at	0.0065	0.1190	*FZD5*	frizzled class receptor 5
206845_s_at	0.0065	0.1551	*RNF40*	ring finger protein 40, E3 ubiquitin protein ligase
238506_at	0.0065	0.1373	*LRRC58*	leucine rich repeat containing 58
219355_at	0.0065	0.1420	*CXorf57*	chromosome X open reading frame 57
215575_at	0.0065	0.1177	*PDE4DIP*	phosphodiesterase 4D interacting protein
1564705_at	0.0066	0.1000	*GLS2*	glutaminase 2 (liver, mitochondrial)
244255_at	0.0066	0.1105	*LOC286114*	uncharacterized LOC286114
207493_x_at	0.0066	0.1102	*SSX2B///SSX2*	synovial sarcoma, X breakpoint 2B///synovial sarcoma, X breakpoint 2
220941_s_at	0.0067	0.1285	*C21orf91*	chromosome 21 open reading frame 91
219493_at	0.0067	0.1313	*SHCBP1*	SHC SH2-domain binding protein 1
228759_at	0.0068	0.1447	*CREB3L2*	cAMP responsive element binding protein 3-like 2
211184_s_at	0.0068	0.1026	*USH1C*	Usher syndrome 1C (autosomal recessive, severe)
224716_at	0.0068	0.1342	*SLC35B2*	solute carrier family 35 (adenosine 3’-phospho 5’-phosphosulfate transporter), member B2
210023_s_at	0.0069	0.1636	*PCGF1*	polycomb group ring finger 1
221900_at	0.0069	0.1290	*COL8A2*	collagen, type VIII, alpha 2
239143_x_at	0.0069	0.1126	*RNF138*	ring finger protein 138, E3 ubiquitin protein ligase
215297_at	0.0069	0.1273	*LOC441204*	uncharacterized LOC441204
225097_at	0.0069	0.1322	*HIPK2*	homeodomain interacting protein kinase 2
1557136_at	0.0070	0.1304	*ATP13A4*	ATPase type 13A4
1557314_at	0.0070	0.1100	*DPY19L2P3*	DPY19L2 pseudogene 3

210191_s_at	0.0070	0.1454	*PHTF1*	putative homeodomain transcription factor 1
1556579_s_at	0.0070	0.1777	*IGSF10*	immunoglobulin superfamily, member 10
242517_at	0.0071	0.2191	*KISS1R*	KISS1 receptor
226261_at	0.0071	0.1274	*ZNRF2*	zinc and ring finger 2
201035_s_at	0.0071	0.1737	*HADH*	hydroxyacyl-CoA dehydrogenase
223381_at	0.0072	0.1172	*NUF2*	NUF2, NDC80 kinetochore complex component
235045_at	0.0072	0.1552	*RBM7*	RNA binding motif protein 7
226859_at	0.0072	0.1247	*DNAJC25*	DnaJ (Hsp40) homolog, subfamily C , member 25
236223_s_at	0.0073	0.1397	*RIT1*	Ras-like without CAAX 1
214987_at	0.0073	0.1400	*GAB1*	GRB2-associated binding protein 1
217738_at	0.0074	0.1493	*NAMPT*	nicotinamide phosphoribosyltransferase
227181_at	0.0074	0.1610	*LNP1*	leukemia NUP98 fusion partner 1
219217_at	0.0074	0.1239	*NARS2*	asparaginyl-tRNA synthetase 2, mitochondrial (putative)
229147_at	0.0075	0.3016	*RASSF6*	Ras association (RalGDS/AF-6) domain family member 6
213321_at	0.0075	0.1270	*BCKDHB*	branched chain keto acid dehydrogenase E1, beta polypeptide
204825_at	0.0076	0.1195	*MELK*	maternal embryonic leucine zipper kinase
203067_at	0.0076	0.0882	*PDHX*	pyruvate dehydrogenase complex, component X
231081_at	0.0076	0.1267	*C2orf73*	chromosome 2 open reading frame 73
220654_at	0.0076	0.1083	*PPY2*	pancreatic polypeptide 2
223301_s_at	0.0076	0.1295	*CCDC82*	coiled-coil domain containing 82
208733_at	0.0077	0.0990	*RAB2A*	RAB2A, member RAS oncogene family
204886_at	0.0077	0.2039	*PLK4*	polo-like kinase 4
232256_s_at	0.0078	0.1385	*LINC00997*	long intergenic non-protein coding RNA 997
229742_at	0.0078	0.1011	*C15orf61*	chromosome 15 open reading frame 61
240429_at	0.0078	0.1244	*ZNF546*	zinc finger protein 546
204004_at	0.0079	0.1234	*PAWR*	PRKC, apoptosis, WT1, regulator
223197_s_at	0.0080	0.0903	*SMARCAD1*	SWI/SNF-related, matrix-associated actin-dependent regulator of chromatin, subfamily a, containing DEAD/H box 1
243501_at	0.0081	0.1105	*ATP5F1*	ATP synthase, H+ transporting, mitochondrial Fo complex, subunit B1
213251_at	0.0082	0.0991	*SMARCA5*	SWI/SNF related, matrix associated, actin dependent regulator of chromatin, subfamily a, member 5
207124_s_at	0.0082	0.1330	*GNB5*	guanine nucleotide binding protein (G protein), beta 5
218256_s_at	0.0082	0.1636	*NUP54*	nucleoporin 54kDa
236250_at	0.0082	0.1424	*AFG3L1P*	AFG3-like AAA ATPase 1, pseudogene
223491_at	0.0082	0.1159	*COMMD2*	COMM domain containing 2
225260_s_at	0.0083	0.1038	*MRPL32*	mitochondrial ribosomal protein L32
237210_at	0.0083	0.1041	*NFRKB*	nuclear factor related to kappaB binding protein
238593_at	0.0083	0.1341	*C11orf80*	chromosome 11 open reading frame 80
205215_at	0.0083	0.1391	*RNF2*	ring finger protein 2
203222_s_at	0.0083	0.1524	*TLE1*	transducin-like enhancer of split 1 (E(sp1) homolog, Drosophila)
206680_at	0.0084	0.2376	*CD5L*	CD5 molecule-like
238078_at	0.0085	0.1382	*SEC22A*	SEC22 vesicle trafficking protein homolog A (S. cerevisiae)
1557248_at	0.0085	0.1848	*ZNF587*	zinc finger protein 587
218701_at	0.0085	0.1659	*LACTB2*	lactamase, beta 2
222765_x_at	0.0085	0.1320	*ESF1*	ESF1, nucleolar pre-rRNA processing protein, homolog (S. cerevisiae)
225153_at	0.0086	0.1202	*GFM1*	G elongation factor, mitochondrial 1
1554121_at	0.0086	0.1326	*HSD17B12*	hydroxysteroid (17-beta) dehydrogenase 12
227200_at	0.0087	0.1587	*ETV3*	ets variant 3
235584_at	0.0087	0.1104	*STARD7-AS1*	STARD7 antisense RNA 1
201963_at	0.0087	0.1107	*ACSL1*	acyl-CoA synthetase long-chain family member 1
226464_at	0.0087	0.1662	*C3orf58*	chromosome 3 open reading frame 58
1565638_at	0.0088	0.1291	*PMP22*	peripheral myelin protein 22
231482_at	0.0088	0.1025	*LINC01206*	long intergenic non-protein coding RNA 1206

205356_at	0.0088	0.1358	*USP13*	ubiquitin specific peptidase 13 (isopeptidase T-3)
227883_at	0.0089	0.0816	*CCDC71L*	coiled-coil domain containing 71-like
224380_s_at	0.0089	0.0888	*TAF7L*	TAF7-like RNA polymerase II, TATA box binding protein (TBP)-associated factor, 50kDa
225492_at	0.0089	0.1082	*TMEM33*	transmembrane protein 33
223267_at	0.0090	0.1187	*TRMT10C*	tRNA methyltransferase 10 homolog C (S. cerevisiae)
227012_at	0.0091	0.1812	*SLC25A40*	solute carrier family 25, member 40
214743_at	0.0091	0.1185	*CUX1*	cut-like homeobox 1
221063_x_at	0.0092	0.1067	*RNF123*	ring finger protein 123
229143_at	0.0092	0.0897	*CNOT3*	CCR4-NOT transcription complex, subunit 3
221505_at	0.0092	0.0839	*ANP32E*	acidic (leucine-rich) nuclear phosphoprotein 32 family, member E
222608_s_at	0.0092	0.1315	*ANLN*	anillin, actin binding protein
1554800_at	0.0092	0.1049	*RAB39A*	RAB39A, member RAS oncogene family
212920_at	0.0092	0.1110	*REST*	RE1-silencing transcription factor
242821_at	0.0092	0.1061	*CCDC171*	coiled-coil domain containing 171
234237_s_at	0.0093	0.0896	*AMBRA1*	autophagy/beclin-1 regulator 1
243337_at	0.0093	0.1189	*FREM3*	FRAS1 related extracellular matrix 3
35974_at	0.0094	0.1240	*LRMP*	lymphoid-restricted membrane protein
1555610_at	0.0094	0.1140	*AGK*	acylglycerol kinase
228449_at	0.0094	0.0878	*MORC2-AS1*	MORC2 antisense RNA 1
226570_at	0.0094	0.1061	*LOC100507375*	uncharacterized LOC100507375
241574_s_at	0.0095	0.1505	*IGF2BP1*	insulin-like growth factor 2 mRNA binding protein 1
1552632_a_at	0.0095	0.1094	*ARSG*	arylsulfatase G
218371_s_at	0.0095	0.1112	*PSPC1*	paraspeckle component 1
237741_at	0.0095	0.0848	*SLC25A36*	solute carrier family 25 (pyrimidine nucleotide carrier), member 36
235638_at	0.0095	0.3741	*RASSF6*	Ras association (RalGDS/AF-6) domain family member 6
206386_at	0.0096	0.1003	*SERPINA7*	serpin peptidase inhibitor, clade A (alpha-1 antiproteinase, antitrypsin), member 7
1554655_a_at	0.0096	0.0966	*RPRML*	reprimo-like
222579_at	0.0097	0.1032	*UBA5*	ubiquitin-like modifier activating enzyme 5
241950_at	0.0098	0.1018	*WWC1*	WW and C2 domain containing 1
209507_at	0.0099	0.1312	*RPA3*	replication protein A3, 14kDa
214086_s_at	0.0099	0.1530	*PARP2*	poly (ADP-ribose) polymerase 2
218460_at	0.0099	0.1103	*HEATR2*	HEAT repeat containing 2
1569745_at	0.0099	0.0927	*OSER1-AS1*	OSER1 antisense RNA 1 (head to head)
228454_at	0.0099	0.1104	*LCOR*	ligand dependent nuclear receptor corepressor
235890_at	0.0099	0.1705	*TBL1XR1*	transducin (beta)-like 1 X-linked receptor 1
202088_at	0.0099	0.0815	*SLC39A6*	solute carrier family 39 (zinc transporter), member 6

Downregulated genes (*P* < 0.01) in FOXP2-positive ABC-DLCBL cases (*n* = 15)

Probe ID	*P*-value	Log (FC)	Gene symbol	Gene name
206099_at	4.66E–06	−0.2961	*PRKCH*	protein kinase C, eta
204037_at	2.34E–05	−0.1934	*LPAR1*	lysophosphatidic acid receptor 1
206388_at	2.4E–05	−0.1760	*PDE3A*	phosphodiesterase 3A, cGMP-inhibited
204497_at	2.75E–05	−0.1760	*ADCY9*	adenylate cyclase 9
226704_at	3E–05	−0.1970	*UBE2J2*	ubiquitin-conjugating enzyme E2, J2
211499_s_at	5.86E–05	−0.2814	*MAPK11*	mitogen-activated protein kinase 11
220567_at	6.11E–05	−0.2278	*IKZF2*	IKAROS family zinc finger 2 (Helios)
217767_at	7.45E–05	−0.2422	*C3*	complement component 3
229910_at	9.58E–05	−0.2147	*SHE*	Src homology 2 domain containing E
205456_at	9.79E–05	−0.2576	*CD3E*	CD3e molecule, epsilon (CD3-TCR complex)
222895_s_at	0.0001	−0.2027	*BCL11B*	B-cell CLL/lymphoma 11B (zinc finger protein)
204834_at	0.0001	−0.2455	*FGL2*	fibrinogen-like 2
243099_at	0.0001	−0.1930	*NFAM1*	NFAT activating protein with ITAM motif 1

219013_at	0.0002	−0.1963	*GALNT11*	polypeptide N-acetylgalactosaminyltransferase 11
212201_at	0.0002	−0.1770	*ANKLE2*	ankyrin repeat and LEM domain containing 2
208349_at	0.0002	−0.2455	*TRPA1*	transient receptor potential cation channel, subfamily A, member 1
1564868_a_at	0.0002	−0.1902	*FAM117B*	family with sequence similarity 117, member B
226950_at	0.0002	−0.2267	*ACVRL1*	activin A receptor type II-like 1
219289_at	0.0002	−0.1449	*HEATR3*	HEAT repeat containing 3
229041_s_at	0.0002	−0.2069	*ITGB2-AS1*	ITGB2 antisense RNA 1
228109_at	0.0002	−0.2172	*RASGRF2*	Ras protein-specific guanine nucleotide-releasing factor 2
227330_x_at	0.0003	−0.1934	*LOC100233156///TEKT4P2///MAFIP*	tektin 4 pseudogene///tektin 4 pseudogene 2///MAFF interacting protein (pseudogene)
205285_s_at	0.0003	−0.1705	*FYB*	FYN binding protein
228719_at	0.0003	−0.1727	*ZSWIM7*	zinc finger, SWIM-type containing 7
206116_s_at	0.0003	−0.1813	*TPM1*	tropomyosin 1 (alpha)
201876_at	0.0003	−0.1991	*PON2*	paraoxonase 2
222450_at	0.0004	−0.1778	*PMEPA1*	prostate transmembrane protein, androgen induced 1
214761_at	0.0004	−0.1663	*ZNF423*	zinc finger protein 423
201580_s_at	0.0005	−0.1719	*TMX4*	thioredoxin-related transmembrane protein 4
221499_s_at	0.0005	−0.1752	*STX16*	syntaxin 16
210038_at	0.0005	−0.1536	*PRKCQ*	protein kinase C, theta
210571_s_at	0.0005	−0.1662	*CMAHP*	cytidine monophospho-N-acetylneuraminic acid hydroxylase, pseudogene
236485_at	0.0005	−0.1428	*LOC100507111*	uncharacterized LOC100507111
202789_at	0.0005	−0.1252	*PLCG1*	phospholipase C, gamma 1
239704_at	0.0006	−0.1557	*RNF144B*	ring finger protein 144B
207330_at	0.0006	−0.1816	*PZP*	pregnancy-zone protein
241624_at	0.0006	−0.1719	*LOC389834*	ankyrin repeat domain 57 pseudogene
206060_s_at	0.0006	−0.1766	*PTPN22*	protein tyrosine phosphatase, non-receptor type 22 (lymphoid)
206804_at	0.0006	−0.1931	*CD3G*	CD3g molecule, gamma (CD3-TCR complex)
222846_at	0.0006	−0.1463	*RAB8B*	RAB8B, member RAS oncogene family
228926_s_at	0.0006	−0.1527	*SMARCA2*	SWI/SNF related, matrix associated, actin dependent regulator of chromatin, subfamily a, member 2
205790_at	0.0006	−0.1224	*SKAP1*	src kinase associated phosphoprotein 1
209487_at	0.0007	−0.1526	*RBPMS*	RNA binding protein with multiple splicing
1554240_a_at	0.0007	−0.2254	*ITGAL*	integrin, alpha L (antigen CD11A (p180), lymphocyte function-associated antigen 1; alpha polypeptide)
228085_at	0.0010	−0.1455	*LOC100507419*	uncharacterized LOC100507419
222912_at	0.0010	−0.1282	*ARRB1*	arrestin, beta 1
205792_at	0.0010	−0.1368	*WISP2*	WNT1 inducible signaling pathway protein 2
217890_s_at	0.0011	−0.2263	*PARVA*	parvin, alpha
230291_s_at	0.0011	−0.2232	*NFIB*	nuclear factor I/B
201301_s_at	0.0011	−0.1132	*ANXA4*	annexin A4
210130_s_at	0.0011	−0.1212	*TM7SF2*	transmembrane 7 superfamily member 2
233072_at	0.0011	−0.1467	*NTNG2*	netrin G2
1552277_a_at	0.0011	−0.1448	*MSANTD3*	Myb/SANT-like DNA-binding domain containing 3
224901_at	0.0011	−0.1918	*SCD5*	stearoyl-CoA desaturase 5
211794_at	0.0012	−0.1332	*FYB*	FYN binding protein
1552395_at	0.0012	−0.1340	*TSSK3*	testis-specific serine kinase 3
224173_s_at	0.0012	−0.1745	*MRPL30*	mitochondrial ribosomal protein L30
223695_s_at	0.0012	−0.1009	*ARSD*	arylsulfatase D
205066_s_at	0.0013	−0.1938	*ENPP1*	ectonucleotide pyrophosphatase/phosphodiesterase 1
207525_s_at	0.0013	−0.1126	*GIPC1*	GIPC PDZ domain containing family, member 1
244519_at	0.0014	−0.1665	*ASXL1*	additional sex combs like transcriptional regulator 1
228428_at	0.0014	−0.1228	*FAM102A*	family with sequence similarity 102, member A
221757_at	0.0015	−0.1477	*PIK3IP1*	phosphoinositide-3-kinase interacting protein 1

211893_x_at	0.0015	−0.1531	*CD6*	CD6 molecule
224359_s_at	0.0015	−0.1315	*HOOK3*	hook microtubule-tethering protein 3
203114_at	0.0015	−0.1533	*SSSCA1*	Sjogren syndrome/scleroderma autoantigen 1
212099_at	0.0015	−0.1384	*RHOB*	ras homolog family member B
32625_at	0.0016	−0.1683	*NPR1*	natriuretic peptide receptor 1
225373_at	0.0016	−0.1094	*C10orf54*	chromosome 10 open reading frame 54
206453_s_at	0.0018	−0.1455	*NDRG2*	NDRG family member 2
228568_at	0.0018	−0.1221	*GCOM1*	GRINL1A complex locus 1
226984_at	0.0018	−0.1697	*FGD5*	FYVE, RhoGEF and PH domain containing 5
212062_at	0.0019	−0.1637	*ATP9A*	ATPase, class II, type 9A
230458_at	0.0019	−0.1525	*SLC45A1*	solute carrier family 45, member 1
227131_at	0.0019	−0.1058	*MAP3K3*	mitogen-activated protein kinase kinase kinase 3
205991_s_at	0.0019	−0.1618	*PRRX1*	paired related homeobox 1
207191_s_at	0.0020	−0.1261	*ISLR*	immunoglobulin superfamily containing leucine-rich repeat
232160_s_at	0.0020	−0.1471	*TNIP2*	TNFAIP3 interacting protein 2
226705_at	0.0020	−0.1506	*FGFR1*	fibroblast growth factor receptor 1
225060_at	0.0021	−0.1939	*LRP11*	low density lipoprotein receptor-related protein 11
210915_x_at	0.0021	−0.1649	*TRBC1*	T cell receptor beta constant 1
202967_at	0.0021	−0.1322	*GSTA4*	glutathione S-transferase alpha 4
215130_s_at	0.0021	−0.1210	*IQCK*	IQ motif containing K
226806_s_at	0.0022	−0.1771	*NFIA*	nuclear factor I/A
206584_at	0.0022	−0.1379	*LY96*	lymphocyte antigen 96
241208_at	0.0023	−0.1243	*PDLIM5*	PDZ and LIM domain 5
1569090_x_at	0.0023	−0.1255	*LINC00957*	long intergenic non-protein coding RNA 957
239525_at	0.0023	−0.1392	*CTTNBP2NL*	CTTNBP2 N-terminal like
1563369_at	0.0023	−0.1761	*LINC00173*	long intergenic non-protein coding RNA 173
202668_at	0.0023	−0.1394	*EFNB2*	ephrin-B2
229697_at	0.0024	−0.1136	*HIRIP3*	HIRA interacting protein 3
1555579_s_at	0.0024	−0.2144	*PTPRM*	protein tyrosine phosphatase, receptor type, M
209597_s_at	0.0024	−0.1820	*PNMA2*	paraneoplastic Ma antigen 2
216919_at	0.0024	−0.1101	*TP53I11*	tumor protein p53 inducible protein 11
1560316_s_at	0.0024	−0.1323	*GLCCI1*	glucocorticoid induced transcript 1
202011_at	0.0024	−0.1421	*TJP1*	tight junction protein 1
216033_s_at	0.0024	−0.1989	*FYN*	FYN proto-oncogene, Src family tyrosine kinase
217683_at	0.0025	−0.1188	*HBE1*	hemoglobin, epsilon 1
228396_at	0.0025	−0.1439	*PRKG1*	protein kinase, cGMP-dependent, type I
203408_s_at	0.0025	−0.1230	*SATB1*	SATB homeobox 1
202944_at	0.0026	−0.1869	*NAGA*	N-acetylgalactosaminidase, alpha-
218223_s_at	0.0026	−0.1706	*PLEKHO1*	pleckstrin homology domain containing, family O member 1
1557628_s_at	0.0026	−0.1637	*LOC283745*	uncharacterized LOC283745
219250_s_at	0.0027	−0.1366	*FLRT3*	fibronectin leucine rich transmembrane protein 3
214023_x_at	0.0027	−0.1826	*TUBB2B*	tubulin, beta 2B class IIb
234541_s_at	0.0027	−0.1258	*ARHGEF12*	Rho guanine nucleotide exchange factor (GEF) 12
221248_s_at	0.0027	−0.1184	*WHSC1L1*	Wolf-Hirschhorn syndrome candidate 1-like 1
203216_s_at	0.0028	−0.1592	*MYO6*	myosin VI
213541_s_at	0.0028	−0.1641	*ERG*	v-ets avian erythroblastosis virus E26 oncogene homolog
207351_s_at	0.0030	−0.1414	*SH2D2A*	SH2 domain containing 2A
1562019_at	0.0030	−0.2464	*NT5DC4*	5’-nucleotidase domain containing 4
1560001_at	0.0030	−0.1625	*LOC100131581*	uncharacterized LOC100131581
218523_at	0.0031	−0.1358	*LHPP*	phospholysine phosphohistidine inorganic pyrophosphate phosphatase
1553530_a_at	0.0031	−0.1835	*ITGB1*	integrin, beta 1 (fibronectin receptor, beta polypeptide, antigen CD29 includes MDF2, MSK12)
219136_s_at	0.0031	−0.1434	*LMF1*	lipase maturation factor 1

1569872_a_at	0.0032	−0.1860	*LMF1*	lipase maturation factor 1
207643_s_at	0.0032	−0.1172	*TNFRSF1A*	tumor necrosis factor receptor superfamily, member 1A
229748_x_at	0.0032	−0.1817	*TEKT4P2*	tektin 4 pseudogene 2
240646_at	0.0032	−0.1469	*GIMAP8*	GTPase, IMAP family member 8
225133_at	0.0032	−0.2101	*KLF3*	Kruppel-like factor 3 (basic)
201059_at	0.0032	−0.1356	*CTTN*	cortactin
228113_at	0.0033	−0.1543	*RAB37*	RAB37, member RAS oncogene family
224457_at	0.0033	−0.2053	*FOXD2-AS1*	FOXD2 antisense RNA 1 (head to head)
240618_at	0.0034	−0.1371	*PTPRA*	protein tyrosine phosphatase, receptor type, A
203128_at	0.0034	−0.0900	*SPTLC2*	serine palmitoyltransferase, long chain base subunit 2
207287_at	0.0035	−0.0981	*BIN3-IT1*	BIN3 intronic transcript 1 (non-protein coding)
222759_at	0.0035	−0.1321	*SUV420H1*	suppressor of variegation 4-20 homolog 1 (Drosophila)
212486_s_at	0.0035	−0.1638	*FYN*	FYN proto-oncogene, Src family tyrosine kinase
216024_at	0.0036	−0.1210	*DNM2*	dynamin 2
215268_at	0.0036	−0.1605	*KIAA0754*	KIAA0754
232981_s_at	0.0036	−0.1545	*SYNRG*	synergin, gamma
216288_at	0.0036	−0.1454	*CYSLTR1*	cysteinyl leukotriene receptor 1
212761_at	0.0037	−0.1949	*TCF7L2*	transcription factor 7-like 2 (T-cell specific, HMG-box)
211902_x_at	0.0037	−0.1497	*TRD*	T cell receptor delta locus
216588_at	0.0037	−0.0960	*RPL7*	ribosomal protein L7
203489_at	0.0037	−0.1106	*SIVA1*	SIVA1, apoptosis-inducing factor
209574_s_at	0.0038	−0.1809	*LDLRAD4*	low density lipoprotein receptor class A domain containing 4
217118_s_at	0.0038	−0.1339	*KIAA0930*	KIAA0930
206142_at	0.0038	−0.1814	*ZNF135*	zinc finger protein 135
210072_at	0.0039	−0.1462	*CCL19*	chemokine (C-C motif) ligand 19
203172_at	0.0041	−0.1105	*FXR2*	fragile X mental retardation, autosomal homolog 2
1552343_s_at	0.0041	−0.1207	*PDE7A*	phosphodiesterase 7A
209722_s_at	0.0041	−0.1019	*SERPINB9*	serpin peptidase inhibitor, clade B (ovalbumin), member 9
1560520_at	0.0041	−0.1707	*LOC401312*	uncharacterized LOC401312
223182_s_at	0.0041	−0.1142	*AGPAT3*	1-acylglycerol-3-phosphate O-acyltransferase 3
208252_s_at	0.0041	−0.0968	*CHST3*	carbohydrate (chondroitin 6) sulfotransferase 3
209083_at	0.0041	−0.1207	*CORO1A*	coronin, actin binding protein, 1A
201341_at	0.0042	−0.1796	*ENC1*	ectodermal-neural cortex 1 (with BTB domain)
210986_s_at	0.0042	−0.1528	*TPM1*	tropomyosin 1 (alpha)
223633_s_at	0.0042	−0.0921	*BCAN*	brevican
224514_x_at	0.0042	−0.1074	*IL17RC*	interleukin 17 receptor C
238461_at	0.0043	−0.1445	*EIF4E3*	eukaryotic translation initiation factor 4E family member 3
213068_at	0.0043	−0.2687	*DPT*	dermatopontin
203528_at	0.0044	−0.1516	*SEMA4D*	sema domain, immunoglobulin domain (Ig), transmembrane domain (TM) and short cytoplasmic domain, (semaphorin) 4D
1556695_a_at	0.0044	−0.0892	*NR2F1-AS1*	NR2F1 antisense RNA 1
205522_at	0.0044	−0.1864	*MIR10B///HOXD4///HOXD3*	microRNA 10b///homeobox D4///homeobox D3
218983_at	0.0044	−0.1614	*C1RL*	complement component 1, r subcomponent-like
235252_at	0.0045	−0.1425	*KSR1*	kinase suppressor of ras 1
205890_s_at	0.0045	−0.0989	*UBD///GABBR1*	ubiquitin D///gamma-aminobutyric acid (GABA) B receptor, 1
214841_at	0.0045	−0.1253	*CNIH3*	cornichon family AMPA receptor auxiliary protein 3
215873_x_at	0.0045	−0.1124	*ABCC10*	ATP-binding cassette, sub-family C (CFTR/MRP), member 10
214908_s_at	0.0046	−0.1620	*TRRAP*	transformation/transcription domain-associated protein
219424_at	0.0046	−0.1651	*EBI3*	Epstein-Barr virus induced 3
1556472_s_at	0.0047	−0.1910	*SCML4*	sex comb on midleg-like 4 (Drosophila)
226571_s_at	0.0047	−0.1376	*PTPRS*	protein tyrosine phosphatase, receptor type, S
206284_x_at	0.0047	−0.1419	*CLTB*	clathrin, light chain B

241045_at	0.0047	−0.1481	*KDM8*	lysine (K)-specific demethylase 8
1569566_at	0.0047	−0.1484	*TBC1D1*	TBC1 (tre-2/USP6, BUB2, cdc16) domain family, member 1
222457_s_at	0.0047	−0.1066	*LIMA1*	LIM domain and actin binding 1
49111_at	0.0047	−0.1224	*ARRB1*	arrestin, beta 1
204261_s_at	0.0048	−0.1509	*PSEN2*	presenilin 2
225562_at	0.0048	−0.1452	*RASA3*	RAS p21 protein activator 3
202191_s_at	0.0049	−0.1566	*GAS7*	growth arrest-specific 7
207081_s_at	0.0049	−0.1504	*PI4KA*	phosphatidylinositol 4-kinase, catalytic, alpha
201131_s_at	0.0049	−0.1421	*CDH1*	cadherin 1, type 1, E-cadherin (epithelial)
210972_x_at	0.0049	−0.1778	*TRAC///TRAJ17///TRAV20*	T cell receptor alpha constant///T cell receptor alpha joining 17///T cell receptor alpha variable 20
1554193_s_at	0.0050	−0.1199	*MANEA*	mannosidase, endo-alpha
208604_s_at	0.0050	−0.1217	*HOXA3*	homeobox A3
1560176_s_at	0.0050	−0.1245	*PPP4R1L*	protein phosphatase 4, regulatory subunit 1-like
224920_x_at	0.0051	−0.1410	*MYADM*	myeloid-associated differentiation marker
235150_at	0.0051	−0.1473	*SESN3*	sestrin 3
204894_s_at	0.0051	−0.1420	*AOC3*	amine oxidase, copper containing 3
204066_s_at	0.0052	−0.1123	*AGAP1*	ArfGAP with GTPase domain, ankyrin repeat and PH domain 1
227550_at	0.0052	−0.1251	*GFRA1*	GDNF family receptor alpha 1
244581_at	0.0053	−0.1671	*ZBTB20*	zinc finger and BTB domain containing 20
1569895_at	0.0053	−0.1365	*LINC01134*	long intergenic non-protein coding RNA 1134
230509_at	0.0053	−0.1405	*SNX22*	sorting nexin 22
1552425_a_at	0.0053	−0.1141	*KLHL10*	kelch-like family member 10
208092_s_at	0.0054	−0.1438	*FAM49A*	family with sequence similarity 49, member A
64064_at	0.0055	−0.0996	*GIMAP5*	GTPase, IMAP family member 5
213316_at	0.0055	−0.1692	*KIAA1462*	KIAA1462
230132_at	0.0055	−0.0985	*PCAT19*	prostate cancer associated transcript 19 (non-protein coding)
233545_at	0.0055	−0.1496	*INPP5D*	inositol polyphosphate-5-phosphatase, 145kDa
214720_x_at	0.0055	−0.1604	*SEPT10*	septin 10
209050_s_at	0.0056	−0.1476	*RALGDS*	ral guanine nucleotide dissociation stimulator
214546_s_at	0.0056	−0.1149	*PPAN-P2RY11///P2RY11*	PPAN-P2RY11 readthrough///purinergic receptor P2Y, G-protein coupled, 11
210210_at	0.0056	−0.1403	*MPZL1*	myelin protein zero-like 1
224588_at	0.0056	−0.4645	*XIST*	X inactive specific transcript (non-protein coding)
208711_s_at	0.0057	−0.1637	*CCND1*	cyclin D1
212912_at	0.0057	−0.1612	*RPS6KA2*	ribosomal protein S6 kinase, 90kDa, polypeptide 2
227662_at	0.0058	−0.2330	*SYNPO2*	synaptopodin 2
212998_x_at	0.0058	−0.1965	*LOC100133583///HLA-DQB1*	HLA class II histocompatibility antigen, DQ beta 1 chain-like///major histocompatibility complex, class II, DQ beta 1
211981_at	0.0058	−0.1777	*COL4A1*	collagen, type IV, alpha 1
242081_at	0.0059	−0.1291	*ACAP1*	ArfGAP with coiled-coil, ankyrin repeat and PH domains 1
226638_at	0.0059	−0.1240	*ARHGAP23*	Rho GTPase activating protein 23
215618_at	0.0059	−0.1123	*RSU1*	Ras suppressor protein 1
209117_at	0.0059	−0.1521	*WBP2*	WW domain binding protein 2
1554445_at	0.0060	−0.1639	*ZNF85*	zinc finger protein 85
1552677_a_at	0.0060	−0.1087	*DIP2A*	DIP2 disco-interacting protein 2 homolog A (Drosophila)
211582_x_at	0.0061	−0.1184	*LST1*	leukocyte specific transcript 1
219563_at	0.0061	−0.1269	*LINC00341*	long intergenic non-protein coding RNA 341
203329_at	0.0061	−0.1788	*PTPRM*	protein tyrosine phosphatase, receptor type, M
216966_at	0.0062	−0.1550	*ITGA2B*	integrin, alpha 2b (platelet glycoprotein IIb of IIb/IIIa complex, antigen CD41)
1555349_a_at	0.0062	−0.1567	*ITGB2*	integrin, beta 2 (complement component 3 receptor 3 and 4 subunit)
222218_s_at	0.0063	−0.1331	*PILRA*	paired immunoglobin-like type 2 receptor alpha

212428_at	0.0063	−0.1212	*KIAA0368*	KIAA0368
225996_at	0.0063	−0.1433	*LONRF2*	LON peptidase N-terminal domain and ring finger 2
228834_at	0.0063	−0.1275	*TOB1*	transducer of ERBB2, 1
232006_at	0.0064	−0.1273	*STK35*	serine/threonine kinase 35
203934_at	0.0064	−0.1303	*KDR*	kinase insert domain receptor (a type III receptor tyrosine kinase)
224189_x_at	0.0064	−0.2142	*EHF*	ets homologous factor
221814_at	0.0064	−0.1465	*GPR124*	G protein-coupled receptor 124
205168_at	0.0065	−0.1281	*DDR2*	discoidin domain receptor tyrosine kinase 2
202598_at	0.0065	−0.1607	*S100A13*	S100 calcium binding protein A13
228367_at	0.0066	−0.1909	*ALPK2*	alpha-kinase 2
218805_at	0.0066	−0.1224	*GIMAP5*	GTPase, IMAP family member 5
236555_at	0.0066	−0.1177	*TRAF3IP2-AS1*	TRAF3IP2 antisense RNA 1
235221_at	0.0066	−0.1738	*CBLN3*	cerebellin 3 precursor
218113_at	0.0067	−0.1746	*TMEM2*	transmembrane protein 2
220705_s_at	0.0067	−0.0969	*ADAMTS7*	ADAM metallopeptidase with thrombospondin type 1 motif, 7
218419_s_at	0.0067	−0.1212	*TMUB2*	transmembrane and ubiquitin-like domain containing 2
244689_at	0.0067	−0.1185	*PPARA*	peroxisome proliferator-activated receptor alpha
216072_at	0.0067	−0.1296	*PRMT2*	protein arginine methyltransferase 2
203183_s_at	0.0068	−0.1503	*SMARCD1*	SWI/SNF related, matrix associated, actin dependent regulator of chromatin, subfamily d, member 1
201384_s_at	0.0068	−0.1020	*NBR1*	neighbor of BRCA1 gene 1
226168_at	0.0068	−0.1593	*ZFAND2B*	zinc finger, AN1-type domain 2B
222449_at	0.0069	−0.1835	*PMEPA1*	prostate transmembrane protein, androgen induced 1
229633_at	0.0070	−0.1614	*INTS10*	integrator complex subunit 10
221646_s_at	0.0070	−0.1753	*ZDHHC11*	zinc finger, DHHC-type containing 11
211192_s_at	0.0071	−0.1314	*CD84*	CD84 molecule
213832_at	0.0071	−0.1325	*KCND3*	potassium voltage-gated channel, Shal-related subfamily, member 3
235116_at	0.0071	−0.1418	*TRAF1*	TNF receptor-associated factor 1
226198_at	0.0071	−0.1057	*TOM1L2*	target of myb1-like 2 (chicken)
227067_x_at	0.0071	−0.0927	*NOTCH2NL*	notch 2 N-terminal like
222055_at	0.0071	−0.0917	*FAHD2CP*	fumarylacetoacetate hydrolase domain containing 2C, pseudogene
1560019_at	0.0071	−0.1246	*DLGAP1-AS2*	DLGAP1 antisense RNA 2
205903_s_at	0.0072	−0.1120	*KCNN3*	potassium intermediate/small conductance calcium-activated channel, subfamily N, member 3
208981_at	0.0072	−0.1097	*PECAM1*	platelet/endothelial cell adhesion molecule 1
207644_at	0.0072	−0.1462	*FOXH1*	forkhead box H1
1559952_x_at	0.0072	−0.2069	*FAM66D///FAM66C*	family with sequence similarity 66, member D///family with sequence similarity 66, member C
223833_at	0.0073	−0.1150	*WDR55*	WD repeat domain 55
201815_s_at	0.0073	−0.1004	*TBC1D5*	TBC1 domain family, member 5
215433_at	0.0073	−0.1353	*DPY19L1*	dpy-19-like 1 (C. elegans)
211920_at	0.0073	−0.1081	*CFB*	complement factor B
1562028_at	0.0074	−0.2001	*CCND3*	cyclin D3
210001_s_at	0.0075	−0.1385	*SOCS1*	suppressor of cytokine signaling 1
216268_s_at	0.0075	−0.1128	*JAG1*	jagged 1
209255_at	0.0075	−0.1608	*KLHDC10*	kelch domain containing 10
210102_at	0.0075	−0.1397	*VWA5A*	von Willebrand factor A domain containing 5A
207129_at	0.0076	−0.1438	*CA5B*	carbonic anhydrase VB, mitochondrial
206994_at	0.0076	−0.1744	*CST4*	cystatin S
213193_x_at	0.0076	−0.1524	*TRBC1*	T cell receptor beta constant 1
1554534_at	0.0076	−0.1159	*DPYD*	dihydropyrimidine dehydrogenase
212325_at	0.0077	−0.1062	*LIMCH1*	LIM and calponin homology domains 1
223108_s_at	0.0078	−0.1204	*ZCCHC17*	zinc finger, CCHC domain containing 17

208890_s_at	0.0078	−0.1242	*PLXNB2*	plexin B2
232531_at	0.0078	−0.1229	*EMX2OS*	EMX2 opposite strand/antisense RNA
224589_at	0.0078	−0.3874	*XIST*	X inactive specific transcript (non-protein coding)
220731_s_at	0.0079	−0.0804	*NECAP2*	NECAP endocytosis associated 2
206666_at	0.0079	−0.1627	*GZMK*	granzyme K (granzyme 3; tryptase II)
209290_s_at	0.0079	−0.1174	*NFIB*	nuclear factor I/B
238730_at	0.0080	−0.1648	*ARHGEF11*	Rho guanine nucleotide exchange factor (GEF) 11
213377_x_at	0.0081	−0.0839	*RPS12*	ribosomal protein S12
233057_at	0.0081	−0.1094	*HSPB8*	heat shock 22kDa protein 8
209500_x_at	0.0081	−0.1344	*TNFSF12-TNFSF13///TNFSF13*	TNFSF12-TNFSF13 readthrough///tumor necrosis factor (ligand) superfamily, member 13
226028_at	0.0082	−0.1065	*ROBO4*	roundabout, axon guidance receptor, homolog 4 (Drosophila)
209522_s_at	0.0082	−0.1189	*CRAT*	carnitine O-acetyltransferase
202746_at	0.0082	−0.1590	*ITM2A*	integral membrane protein 2A
212993_at	0.0083	−0.1688	*NACC2*	NACC family member 2, BEN and BTB (POZ) domain containing
217024_x_at	0.0083	−0.1086	*SIRPA*	signal-regulatory protein alpha
233936_s_at	0.0083	−0.1110	*GGNBP2*	gametogenetin binding protein 2
226403_at	0.0083	−0.1094	*TMC4*	transmembrane channel-like 4
211210_x_at	0.0084	−0.1163	*SH2D1A*	SH2 domain containing 1A
230866_at	0.0084	−0.1536	*CYSLTR1*	cysteinyl leukotriene receptor 1
215332_s_at	0.0084	−0.1435	*CD8B*	CD8b molecule
208664_s_at	0.0084	−0.1154	*TTC3*	tetratricopeptide repeat domain 3
220362_at	0.0085	−0.0812	*PSORS1C1*	psoriasis susceptibility 1 candidate 1
1558972_s_at	0.0085	−0.1513	*THEMIS*	thymocyte selection associated
210815_s_at	0.0085	−0.0987	*CALCRL*	calcitonin receptor-like
227265_at	0.0085	−0.1057	*FGL2*	fibrinogen-like 2
201278_at	0.0086	−0.1266	*DAB2*	Dab, mitogen-responsive phosphoprotein, homolog 2 (Drosophila)
239421_at	0.0086	−0.1328	*DLGAP1-AS1*	DLGAP1 antisense RNA 1
223492_s_at	0.0086	−0.0983	*LRRFIP1*	leucine rich repeat (in FLII) interacting protein 1
213438_at	0.0086	−0.1550	*NFASC*	neurofascin
229626_at	0.0086	−0.1142	*CCDC184*	coiled-coil domain containing 184
217867_x_at	0.0087	−0.1525	*BACE2*	beta-site APP-cleaving enzyme 2
235471_at	0.0087	−0.1260	*VSTM4*	V-set and transmembrane domain containing 4
212130_x_at	0.0087	−0.1070	*EIF1*	eukaryotic translation initiation factor 1
227645_at	0.0088	−0.1677	*PIK3R5*	phosphoinositide-3-kinase, regulatory subunit 5
203904_x_at	0.0088	−0.1315	*CD82*	CD82 molecule
219427_at	0.0088	−0.1157	*FAT4*	FAT atypical cadherin 4
227771_at	0.0088	−0.1738	*LIFR*	leukemia inhibitory factor receptor alpha
243212_at	0.0088	−0.1112	*LOC100505820*	uncharacterized LOC100505820
1558136_s_at	0.0089	−0.0925	*TAF11*	TAF11 RNA polymerase II, TATA box binding protein (TBP)-associated factor, 28kDa
202192_s_at	0.0089	−0.1522	*GAS7*	growth arrest-specific 7
202013_s_at	0.0089	−0.1366	*EXT2*	exostosin glycosyltransferase 2
201211_s_at	0.0090	−0.0907	*DDX3X*	DEAD (Asp-Glu-Ala-Asp) box helicase 3, X-linked
220097_s_at	0.0090	−0.1424	*TMEM104*	transmembrane protein 104
233510_s_at	0.0090	−0.1200	*PARVG*	parvin, gamma
1553397_at	0.0091	−0.1204	*CCDC13*	coiled-coil domain containing 13
204517_at	0.0091	−0.1280	*PPIC*	peptidylprolyl isomerase C (cyclophilin C)
207187_at	0.0091	−0.1296	*JAK3*	Janus kinase 3
227439_at	0.0091	−0.1074	*ANKS1B*	ankyrin repeat and sterile alpha motif domain containing 1B
222057_at	0.0093	−0.1906	*NOL12*	nucleolar protein 12
227494_at	0.0093	−0.1096	*LOC253842///NR6A1*	uncharacterized LOC253842///nuclear receptor subfamily 6, group A, member 1

229215_at	0.0094	−0.1672	*ASCL2*	achaete-scute family bHLH transcription factor 2
221260_s_at	0.0094	−0.1445	*CSRNP2*	cysteine-serine-rich nuclear protein 2
229290_at	0.0094	−0.1028	*DAPL1*	death associated protein-like 1
1554736_at	0.0094	−0.1036	*ARHGAP29*	Rho GTPase activating protein 29
220532_s_at	0.0096	−0.1165	*TMEM176B*	transmembrane protein 176B
237045_at	0.0096	−0.1084	*FAM91A1*	family with sequence similarity 91, member A1
38340_at	0.0096	−0.1365	*HIP1R*	huntingtin interacting protein 1 related
211622_s_at	0.0097	−0.0825	*ARF3*	ADP-ribosylation factor 3
238575_at	0.0097	−0.1428	*OSBPL6*	oxysterol binding protein-like 6
1559883_s_at	0.0097	−0.1204	*SAMHD1*	SAM domain and HD domain 1
225185_at	0.0097	−0.1129	*MRAS*	muscle RAS oncogene homolog
212667_at	0.0098	−0.1881	*SPARC*	secreted protein, acidic, cysteine-rich (osteonectin)
49452_at	0.0098	−0.1354	*ACACB*	acetyl-CoA carboxylase beta
212713_at	0.0098	−0.2159	*MFAP4*	microfibrillar-associated protein 4
1557116_at	0.0099	−0.1211	*APOL6*	apolipoprotein L, 6
214428_x_at	0.0099	−0.1534	*C4B///C4A*	complement component 4B (Chido blood group)///complement component 4A (Rodgers blood group)
219777_at	0.0099	−0.1081	*GIMAP6*	GTPase, IMAP family member 6
234332_at	0.0099	−0.0964	*NUB1*	negative regulator of ubiquitin-like proteins 1
232898_at	0.0099	−0.1770	*DAB2*	Dab, mitogen-responsive phosphoprotein, homolog 2 (Drosophila)
242851_at	0.0099	−0.1638	*KIAA1919*	KIAA1919
209505_at	0.0099	−0.1298	*NR2F1*	nuclear receptor subfamily 2, group F, member 1
233893_s_at	0.0099	−0.0902	*UVSSA*	UV-stimulated scaffold protein A

Supplementary Table S2: Gene Ontology (GO) terms and pathways enriched within top 100 genes negatively-associated with FOXP2 protein expression in DLBCL cases irrespective of COO subtype (*n* = 39) or ABC-DLBCL (*n* = 15) cases

Gene Ontology (GO) terms and pathways enriched within top 100 genes negatively-associated with FOXP2 protein expression in DLBCL cases irrespective of COO subtype (*n* = 39)

ID	Signature	Name	Category	*P*-value	*q*-value FDR B & H	[-log10 (FDR)]	Hit Count in Query List	Hit Count in Genome	Hit in Query List
GO:0051128	Cellular component & clathrin coat assembly	regulation of cellular component organization	GO: Biological Process	1.79E–05	0.0341	1.4678	22	1733	WBP2, NPR1, CDC42EP4,SYNJ2BP,MAPT,DAB2, EPB41L5, CLIC4, SIN3A, BTBD9, ITGB1, GFA, STRIP2, NOX1, SNAP91, ARHGEF10, SMARCA4, FGD5, TRIM40, DNAJB2, DCBLD2, ZMYM3
GO:0048268		clathrin coat assembly	GO: Biological Process	4.71E–05	0.0449	1.3477	3	15	DAB2, SNAP91, PIK3C2A
GO:0032989		cellular component morphogenesis	GO: Biological Process	7.79E–05	0.0495	1.3054	17	1242	FNDC3B, CDC42EP4, MAPT, KIAA0586, DAB2, EPB41L5, PTPN23, CLIC4, OPA3, ITGB1, STRIP2, NEO1, SNAP91, NFIB, SMARCA4, FGD5, ZMYM3

Gene Ontology (GO) terms and pathways enriched within top 100 genes negatively-associated with FOXP2 protein expression in ABC-DLBCL cases (*n* = 15)

ID	Signature	Name	Category	*P*–value	*q*–value FDR B&H	[-log10 (FDR)]	Hit Count in Query List	Hit Count in Genome	Hit in Query List
GO:0050851	Immune response	antigen receptor-mediated signaling pathway	GO: Biological Process	1.52E–10	3.265E–07	6.4861	11	147	PTPN22,NFAM1,CD3E,CD3G,SKAP1,PRKCH,PRKCQ,PLCG1,TRBC1,FYB,FYN
GO:0002757		immune response-activating signal transduction	GO: Biological Process	5.00E–09	2.37E–06	5.6253	14	381	TNIP2,PTPN22,LY96,NFAM1,CD3E,CD3G, SKAP1,PRKCH,PRKCQ,PLCG1,TRBC1,MAPK11,FYB,FYN
GO:0002253		activation of immune response	GO: Biological Process	2.98E–09	2.37E–06	5.6253	15	433	TNIP2,PTPN22,LY96,NFAM1,CD3E,CD3G, SKAP1,C3,PRKCH,PRKCQ,PLCG1,TRBC1, MAPK11,FYB,FYN
GO:0002764		immune response-regulating signaling pathway	GO: Biological Process	1.57E–08	5.624E–06	5.2500	15	490	TNIP2,PTPN22,LY96,NFAM1,CD3E,CD3G,SKAP1,PRKCH,PRKCQ,FGFR1,PLCG1,TRBC1,MAPK11,FYB,FYN
GO:0002429		immune response-activating cell surface receptor signaling pathway	GO: Biological Process	2.06E–08	6.334E–06	5.1983	11	234	PTPN22,NFAM1,CD3E,CD3G,SKAP1,PRKCH,PRKCQ,PLCG1,TRBC1,FYB,FYN
GO:0050778		positive regulation of immune response	GO: Biological Process	3.81E–08	1.024E–05	4.9897	15	524	TNIP2,PTPN22,LY96,NFAM1,CD3E,CD3G, SKAP1,C3,PRKCH,PRKCQ,PLCG1,TRBC1, MAPK11,FYB,FYN
GO:0050776		regulation of immune response	GO: Biological Process	5.78E–08	1.383E–05	4.8592	18	801	TNIP2,PTPN22,LY96,ITGAL,NFAM1,C10orf54,CD3E,CD3G,SKAP1,C3,PRKCH,PRKCQ,FGFR1,PLCG1,TRBC1,MAPK11,FYB,FYN
GO:0002768		immune response-regulating cell surface receptor signaling pathway	GO: Biological Process	1.75E–07	3.417E–05	4.4664	12	356	PTPN22,NFAM1,CD3E,CD3G,SKAP1,PRKCH,PRKCQ,FGFR1,PLCG1,TRBC1,FYB,FYN
GO:0002684		positive regulation of immune system process	GO: Biological Process	5.06E–07	7.268E–05	4.1386	16	732	TNIP2,PTPN22,LY96,ITGAL,NFAM1,CD3E,CD3G,SKAP1,C3,PRKCH,PRKCQ,PLCG1,TRBC1,MAPK11,FYB,FYN
GO:0006955		immune response	GO: Biological Process	1.43E–05	0.0015	2.8119	20	1416	ENPP1,TNIP2,PTPN22,LY96,ITGAL,NFAM1,ADCY9,C10orf54,CD3E,CD3G,SKAP1,C3,PRKCH,PRKCQ,FGFR1,PLCG1,TRBC1,MAPK11,FYB,FYN
GO:0002682		regulation of immune system process	GO: Biological Process	2.15E–05	0.0021	2.6774	18	1212	TNIP2,PTPN22,LY96,ITGAL,NFAM1,C10orf54,CD3E,CD3G,SKAP1,C3,PRKCH,PRKCQ,FGFR1,PLCG1,TRBC1,MAPK11,FYB,FYN
366160		Adaptive Immune System	BioSystems: REACTOME	4.52E–05	0.0033	2.4803	13	648	RNF144B,ITGAL,CD3E,CD3G,UBE2J2,C3,PRKCQ,FGFR1,PLCG1,PRKG1,TRBC1, FYB,FYN

GO:0050852	TCR signaling	T cell receptor signaling pathway	GO: Biological Process	4.82E–09	2.37E–06	5.6253	9	114	PTPN22,CD3E,CD3G,SKAP1,PRKCQ,PLCG1,TRBC1,FYB,FYN
137998		TCR signaling in naive CD4+ T cells	BioSystems: Pathway Interaction Database	1.02E–06	0.0002	3.6055	6	61	CD3E,CD3G,PRKCQ,PLCG1,FYB,FYN
106414		TCR signaling	BioSystems: REACTOME	1.36E–06	0.0002	3.6055	6	64	CD3E,CD3G,PRKCQ,PLCG1,TRBC1,FYB
198862		TCR Signaling Pathway	BioSystems: WikiPathways	6.59E–07	0.0002	3.6055	7	91	CD3E,CD3G,SKAP1,PRKCQ,PLCG1,FYB,FYN
138055		TCR signaling in naive CD8+ T cells	BioSystems: Pathway Interaction Database	7.21E–06	0.0011	2.9788	5	49	CD3E,CD3G,PRKCQ,PLCG1,FYN
83080		T cell receptor signaling pathway	BioSystems: KEGG	2.32E–05	0.0021	2.6753	6	104	CD3E,CD3G,PRKCQ,PLCG1,MAPK11,FYN
GO:0042608		T cell receptor binding	GO: Molecular Function	6.30E–06	0.0024	2.6265	3	8	CD3E,CD3G,FYN
M10765		Lck and Fyn tyrosine kinases in initiation of TCR Activation	MSigDB C2: BioCarta	4.55E–05	0.0033	2.4803	3	13	CD3E,CD3G,FYN
GO:0042110		T cell activation	GO: Biological Process	4.82E–05	0.0035	2.4612	10	428	PTPN22,ITGAL,C10orf54,CD3E,CD3G,BCL11B,SATB1,PRKCQ,TRBC1,FYN
M13247		T Cytotoxic Cell Surface Molecules	MSigDB C2: BioCarta	5.76E–05	0.0035	2.4566	3	14	ITGAL,CD3E,CD3G
M6427		T Helper Cell Surface Molecules	MSigDB C2: BioCarta	5.76E–05	0.0035	2.4566	3	14	ITGAL,CD3E,CD3G
GO:0050863		regulation of T cell activation	GO: Biological Process	5.58E–05	0.0038	2.4241	8	270	PTPN22,ITGAL,C10orf54,CD3E,CD3G,PRKCQ,TRBC1,FYN
M1462		CTL mediated immune response against target cells	MSigDB C2: BioCarta	7.17E–05	0.0040	2.3961	3	15	ITGAL,CD3E,CD3G
GO:0048584	Stimulus & signal transduction	positive regulation of response to stimulus	GO: Biological Process	5.50E–09	2.37E–06	5.6253	27	1584	GIPC1,ASXL1,PRRX1,RBPMS,ZNF423,TNIP2,PTPN22,LY96,ACVRL1,ITGAL,NFAM1,LPAR1,C10orf54,MAP3K3,CD3E,CD3G,ARRB1,SKAP1,C3,PRKCH,PRKCQ,FGFR1,PLCG1,TRBC1,MAPK11,FYB,FYN
GO:0009967		positive regulation of signal transduction	GO: Biological Process	8.19E–08	1.762E–05	4.7540	21	1118	GIPC1,ASXL1,PRRX1,RBPMS,ZNF423,TNIP2,LY96,ACVRL1,ITGAL,NFAM1,LPAR1,C10orf54,MAP3K3,CD3E,ARRB1,SKAP1,C3,PRKCH,FGFR1,MAPK11,FYN
GO:0023056		positive regulation of signaling	GO: Biological Process	2.27E–07	4.072E–05	4.3902	21	1188	GIPC1,ASXL1,PRRX1,RBPMS,ZNF423,TNIP2,LY96,ACVRL1,ITGAL,NFAM1,LPAR1,C10orf54,MAP3K3,CD3E,ARRB1,SKAP1,C3,PRKCH,FGFR1,MAPK11,FYN
GO:0010647		positive regulation of cell communication	GO: Biological Process	2.54E–07	4.203E–05	4.3764	21	1196	GIPC1,ASXL1,PRRX1,RBPMS,ZNF423,TNIP2,LY96,ACVRL1,ITGAL,NFAM1,LPAR1,C10orf54,MAP3K3,CD3E,ARRB1,SKAP1,C3,PRKCH,FGFR1,MAPK11,FYN
GO:0007167		enzyme linked receptor protein signaling pathway	GO: Biological Process	9.50E–06	0.0012	2.9126	17	1028	GIPC1,NPR1,RBPMS,RASGRF2,ENPP1,ZNF423,ACVRL1,PMEPA1,ADCY9,C10orf54,CD3E,EFNB2,PRKCQ,FGFR1,PLCG1,MAPK11,FYN
GO:0051249	Lymphocyte activation	regulation of lymphocyte activation	GO: Biological Process	1.02E–05	0.0012	2.9126	10	357	TNIP2,PTPN22,ITGAL,NFAM1,C10orf54,CD3E,CD3G,PRKCQ,TRBC1,FYN
GO:0002694		regulation of leukocyte activation	GO: Biological Process	3.02E–05	0.0025	2.5950	10	405	TNIP2,PTPN22,ITGAL,NFAM1,C10orf54,CD3E,CD3G,PRKCQ,TRBC1,FYN
GO:0046649		lymphocyte activation	GO: Biological Process	3.12E–05	0.0025	2.5950	12	589	TNIP2,PTPN22,ITGAL,NFAM1,C10orf54,CD3E,CD3G,BCL11B,SATB1,PRKCQ,TRBC1,FYN
GO:0045321		leukocyte activation	GO: Biological Process	3.46E–05	0.0027	2.5753	13	695	TNIP2,PTPN22,ITGAL,NFAM1,C10orf54,CD3E,CD3G,BCL11B,SATB1,PRKCQ,TRBC1,FYB,FYN

GO:0001775	Miscellaneous	cell activation	GO: Biological Process	4.24E–07	6.523E–05	4.1856	18	916	TNIP2,PTPN22,ACVRL1,ITGAL,NFAM1,C10orf54,RHOB,CD3E,CD3G,BCL11B,ARRB1,SATB1,PRKCH,PRKCQ,PRKG1,TRBC1,FYB,FYN
106417		Generation of second messenger molecules	BioSystems: REACTOME	1.12E–06	0.0002	3.6055	5	34	CD3E,CD3G,PLCG1,TRBC1,FYB
GO:0050865		regulation of cell activation	GO: Biological Process	9.85E–06	0.0012	2.9126	11	437	TNIP2,PTPN22,ITGAL,NFAM1,C10orf54,CD3E,CD3G,PRKCQ,PRKG1,TRBC1,FYN
GO:0050793		regulation of developmental process	GO: Biological Process	1.21E–05	0.0014	2.8630	24	1912	ASXL1,NPR1,PDE3A,ENPP1,ACVRL1,NFAM1,LPAR1,C10orf54,PDLIM5,RHOB,CD3E,BCL11B,HOOK3,EFNB2,PTPRM,NFIB, PARVA,C3,PRKCH,FGD5,FGFR1,PLCG1,MAPK11,FYN
GO:0030030		cell projection organization	GO: Biological Process	1.90E–05	0.0019	2.7102	18	1201	TPM1,RAB8B,LPAR1,PDLIM5,RHOB,NTNG2,BCL11B,EFNB2,PTPRM,GALNT11,NFIB,PARVA,FGD5,PRKCQ,FGFR1,PLCG1, PRKG1,FYN
P00021		FGF signaling pathway	PantherDB	2.08E–05	0.0021	2.6753	6	102	MAP3K3,PRKCH,PRKCQ,FGFR1,PLCG1, MAPK11
868086		Rap1 signaling pathway	BioSystems: KEGG	2.19E–05	0.0021	2.6753	8	213	ITGAL,LPAR1,ADCY9,SKAP1,FGFR1,PLCG1,MAPK11,FYB
GO:0006468		protein phosphorylation	GO: Biological Process	2.32E–05	0.0022	2.6623	20	1464	NPR1,RBPMS,ENPP1,ACVRL1,LPAR1,ADCY9,MAP3K3,CD3E,ARRB1,CD6,C3,PRKCH,PRKCQ,FGFR1,PLCG1,PRKG1,TSSK3,MAPK11,FYB,FYN
GO:0042060		wound healing	GO: Biological Process	2.80E–05	0.0025	2.6000	13	681	TPM1,PDE3A,ACVRL1,ITGAL,RHOB,ARRB1,C3,PRKCH,PRKCQ,PLCG1,PRKG1, FYN,HBE1
GO:0050855		regulation of B cell receptor signaling pathway	GO: Biological Process	3.19E–05	0.0025	2.5950	3	13	PTPN22,NFAM1,PRKCH
GO:0019900		kinase binding	GO: Molecular Function	1.60E–05	0.0030	2.5236	12	552	NPR1,TNIP2,PTPN22,ACVRL1,PDLIM5,PIK3IP1,CD3E,ARRB1,CD6,SKAP1,PLCG1,PRKG1
GO:0090100		positive regulation of transmembrane receptor protein serine/threonine kinase signaling pathway	GO: Biological Process	4.38E–05	0.0033	2.4878	5	79	GIPC1,RBPMS,ZNF423,ACVRL1,C10orf54
GO:0007596		blood coagulation	GO: Biological Process	5.69E–05	0.0038	2.4241	11	529	PDE3A,ITGAL,RHOB,ARRB1,C3,PRKCH,PRKCQ,PLCG1,PRKG1,FYN,HBE1
GO:0016310		phosphorylation	GO: Biological Process	5.77E–05	0.0038	2.4241	23	1963	NPR1,RBPMS,ENPP1,ACVRL1,ANKLE2,NDRG2,LPAR1,ADCY9,MAP3K3,PIK3IP1,CD3E,ARRB1,CD6,C3,PRKCH,PRKCQ, FGFR1,PLCG1,PRKG1,TSSK3,MAPK11,FYB,FYN
GO:0050817		coagulation	GO: Biological Process	5.99E–05	0.0038	2.4209	11	532	PDE3A,ITGAL,RHOB,ARRB1,C3,PRKCH,PRKCQ,PLCG1,PRKG1,FYN,HBE1
GO:0007599		hemostasis	GO: Biological Process	6.20E–05	0.0038	2.4190	11	534	PDE3A,ITGAL,RHOB,ARRB1,C3,PRKCH,PRKCQ,PLCG1,PRKG1,FYN,HBE1
